# A Study on ML-Based Software Defect Detection for Security Traceability in Smart Healthcare Applications

**DOI:** 10.3390/s23073470

**Published:** 2023-03-26

**Authors:** Samuel Mcmurray, Ali Hassan Sodhro

**Affiliations:** 1 Department of Computer Science, Kristianstad University, SE-29188 Kristianstad, Sweden; 2 School of Engineering, Jönköping University, SE-55318 Jönköping, Sweden

**Keywords:** machine learning, feature extraction, feature selection, ensemble learning, software defects prediction, software development life-cycle

## Abstract

Software Defect Prediction (SDP) is an integral aspect of the Software Development Life-Cycle (SDLC). As the prevalence of software systems increases and becomes more integrated into our daily lives, so the complexity of these systems increases the risks of widespread defects. With reliance on these systems increasing, the ability to accurately identify a defective model using Machine Learning (ML) has been overlooked and less addressed. Thus, this article contributes an investigation of various ML techniques for SDP. An investigation, comparative analysis and recommendation of appropriate Feature Extraction (FE) techniques, Principal Component Analysis (PCA), Partial Least Squares Regression (PLS), Feature Selection (FS) techniques, Fisher score, Recursive Feature Elimination (RFE), and Elastic Net are presented. Validation of the following techniques, both separately and in combination with ML algorithms, is performed: Support Vector Machine (SVM), Logistic Regression (LR), Naïve Bayes (NB), K-Nearest Neighbour (KNN), Multilayer Perceptron (MLP), Decision Tree (DT), and ensemble learning methods Bootstrap Aggregation (Bagging), Adaptive Boosting (AdaBoost), Extreme Gradient Boosting (XGBoost), Random Forest(RF), and Generalized Stacking (Stacking). Extensive experimental setup was built and the results of the experiments revealed that FE and FS can both positively and negatively affect performance over the base model or Baseline. PLS, both separately and in combination with FS techniques, provides impressive, and the most consistent, improvements, while PCA, in combination with Elastic-Net, shows acceptable improvement.

## 1. Introduction

The subject of how software defects can be investigated, predicted, and reduced has been in contention with Computer Scientists for decades. Kadadevaramath et al. [1] revealed that, along with software delivery cost and schedule, the quality of the delivered software is also important to the growth and survival of a software organization. Lowering the software defect density is a multistage process throughout the development life-cycle. Boehm et al. [2] stated that accelerated development time affects the scheduling and complexity of software production and makes it difficult to avoid defects. The results of their research showed that analysis tools and testing are two different classes of approach to defects at different stages. Resolving defects early in the development cycle creates better and more cost effective workflow throughout the entire process.

Defects can be reduced within the Software Development Life-Cycle (SDLC) via software management practices; in particular, the use of the Agile Methodology. Collaboration between developers and operators in adopting agile methodology via short cycle iterations is described by Leite et al. [3]. Development Operation (DevOps) is an extension of Agile Battina [4], and describes the DevOps’ strategic goal of fulfilling the demands of the client with the help of technologies and processes. Through proper software management within DevOps defects can be reduced to improve product quality, service quality and client satisfaction, as explained by Leite et al. [3]. Wang et al. [5] presented the correlation between software systems and defects, and they further pointed out that, as software systems grow, both in size and complexity, the more challenges there are in identifying defects.

Saharudin et al. [6] found that defects can occur at any stage during the development process, possibly remaining hidden and only becoming active at deployment. This has many real-world consequences or drawbacks, as ever-evolving software becomes more integrated into many aspects of our daily lives. According to Boehm et al. [2], on average, roughly 80% of defects are observed within 20% of modules.

Looking at predictions independently, via classification or frequency of possible software defects, the goal is to ensure the quality of software being deployed matches the expectations of organizations/clients, while reducing costs, by improving the quality, maintainability, and deliverability of the software. The use of Machine Learning (ML) techniques within Software Defect Prediction (SDP) has the potential to further minimize cost, improve performance, quality and consumer experience by producing a better product in a faster development cycle.

According to Mrinal et al., identifying defects improves the software system and provides better insight to project managers, resulting in their making informed decisions about planning and execution of development cycles [7]. SDP can limit the number of defects, resulting in reduced development time, increased reliability, reduced rework, and improved client/stakeholder satisfaction. Thus, it can be said that the development of reliable SDP models is integral to SDLC and DevOps, to identify patterns or anti-patterns that can reduce defects that may have been missed in other processes.

### 1.1. Background

According to the IEEE 729–1983 Standard Glossary of Software Engineering Terminology [8], a defect or bug is synonymous with a fault. A software defect is described as the result of an error that, if left uncorrected, produces incorrect/inconsistent values in the software as a result of human action, or results in functional units being unable to perform the required task. A software defect can result in varying types of problems, which, according to Kalaivani et al., need to be diagnosed and acted on at an early phase within the SDLC, so as to manage and monitor software defects [9]. To handle defects, it is necessary to do the following: first, identify the defects; second, categorize the defects; third, analyze the defects; finally, predict and remove remaining defects.

Agile development methodology was released as a manifesto in 2001, and developed by software community industry leaders through their personal experiences and expertise, based on accepted practices, according to Dingsøyr et al. [10]. Agile principles emphasize collaborative development to allow for an open and shared process pipeline. One of the principles of lean software development is reducing work to its core aspects by, for instance, reducing extensive documentation. The end goal is to deliver working software to clients. Agile has made it possible for development teams to deliver high quality code in a timely manner that responds to stakeholder requirements and can embrace changes.

**Figure 1:**
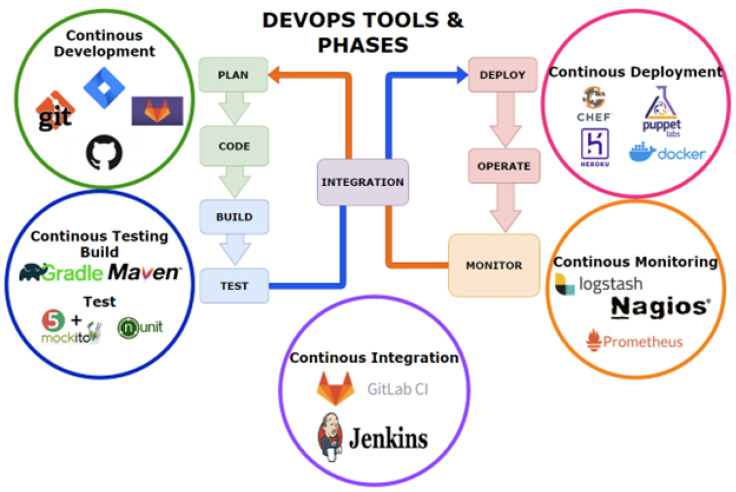
DevOps Tools and Phases.

DevOps, another software methodology, as discussed by Leite et al. [3], is a culture of collaboration, of both knowledge and tools, which aims to establish a relationship between processes and practices. As stated by Ruf et al., DevOps emerged from the agile methodology, by means of extending a more streamlined process through the build, test, deploy, and delivery stages [11]. The change in the agile methodology was the focus on project management for software engineers and developers, ’Devs’, who are responsible for translating ideas, directly received from clients/stakeholders, into code. Including operational Engineers and IT specialists, responsible for the deployment and monitoring of a responsive system, is useful. Additionally, Continuous Integration/Continuous Delivery (CI/CD), where developers frequently merge codes into a primary repository, makes it possible to deliver the latest features to end users. Three key goals within DevOps are discussed by Leite et al. [3]: first, assisting in collaboration between departments; second, providing continuous delivery; lastly, maintaining software reliability. Figure 1 depicts the phases and tools within DevOps.

Boehm et al. [2] presented a number of software rework reduction strategies. Some challenges, such as breakage of code, architecture, and design, were observed, due to avoidance of rework improvements to the architecture, software process maturity, and risk management. The use of data analyzing and testing tools detects defects at different levels in the SDLC. For instance, DevOps has reduced many of these defects through incorporation of automated and well-defined management structures.

Prasad et al. [12] proposed an SDP prediction model, with metrics as independent collected variables, for SDLC. The prediction of software defects is primarily carried out through the use of software metrics extracted from the Open Static Analyzer program. This open source analyzer measures the static source code, based on the Size Metrics found in Table 1, in addition to Complexity Metrics, Coupling Metrics, Documentation Metrics, Cohesion Metrics, Inheritance Metrics, and code duplication metrics, known as Clone Metrics, and found in Table 2. As stated by Kalaivani et al. [9] and Prasad et al. [12] these metrics are known as product metrics. In addition to the above metrics, there are also other metrics, such as process metrics, which are used to improve software development and maintenance, and project metrics, including cost, schedule, productivity, and developer information over the life-cycle.

Mehta et al. [13] presented four types of product metrics for SDP. First, object-oriented metrics, in which software properties, such as cohesion, inheritance, and the coupling of classes, are measured. Second, traditional metrics, as seen in Table 3, which present the product metrics found in the NASA Metrics Data Program data set for SDP, using Halstead and McCabe indicators to measure the complexity and size of reported software systems i.e., line count of code. According to the theory put forward by McCabe [14], known as graph–theoretic complexity, in a strongly connected graph the maximum number of linearly independent circuits has a direct correlation to complexity. Halstead argued that the more complex and harder a code is in reading, the more likely it is to have defects/faults. Third, hybrid metrics, which are a combination of object-oriented and traditional metrics. Finally, the fourth type of product metrics is called miscellaneous metrics, in which the metrics fall under no specific category.

Once the data from the source code is extracted, it can be labeled and used in the SDP process and split into two categories, as stated by Akimova et al. [15]. The first is the manual approach, which is the result of manual testing and code review.

The second automatic approach, which improves productivity by lowering overall cost, has made the latter a more promising and desirable choice. As presented by Miñón et al. [16], recent advances have been observed in both hardware, i.e., graphical processing units (GPUs) of modern computers and reduction in their cost, and in ML algorithms to identify hidden patterns. The impressive ability of ML algorithms to process a large amount of data into more meaningful information for end-users has attracted many industries; for example, healthcare for early disease prediction, as stated by Khan et al. [17]. Predictions of software defects can be further broken down in classification, i.e., whether the module is defective or not, and defect density, which is presented by Kadadevaramath et al. [1], in the domain of the financial industry.

In any case, the first essential requirement for the implementation of SDP is data or software metrics, the collection, validation, and storage of which is an on-going project. Ideally, the project should use well-defined SDLC to collect data during the continuous testing phase of the DevOps process.

**Figure 2:**
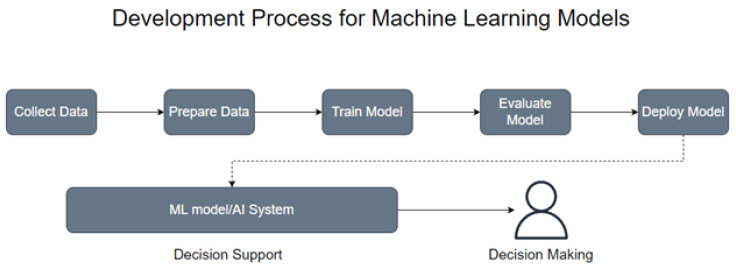
The Model Building Process in Machine Learning.

After various instances of project analysis at each stage, the data can be validated and stored in the database, after which, model building and selection processes begin. The ML model building process is depicted in Figure 2, where the data is collected, stored, and cleaned, by removing unwanted features and by the proper handling of missing values. Then, the model is trained on a portion of the data, known as the training set, by applying scaling and feature reduction models to properly train the ML algorithm on the resulting data.

In the model evaluation, the remaining portion, or test set, is tested on the learned model, where the results can be evaluated. Once the models have been built and evaluated through numerous tests, then the chosen model can be implemented and deployed within the SDLC. The organization would continue to collect instances, developing updated or new databases to build new, or improve on existing, algorithms for their SDP.

**Figure 3:**
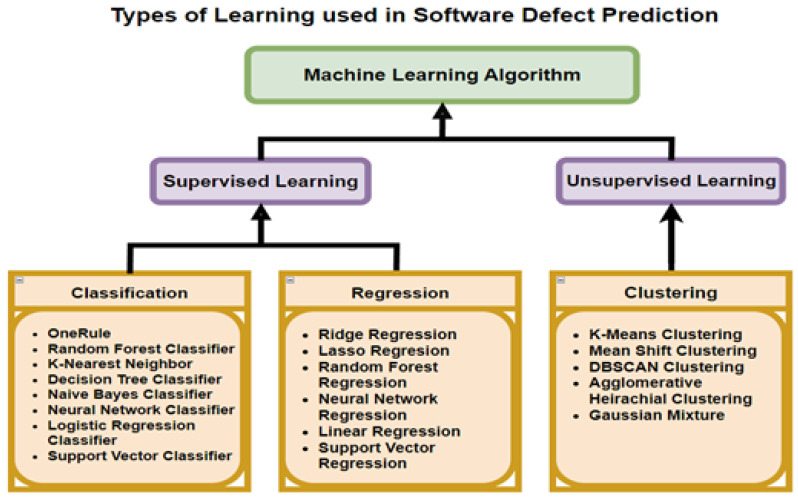
Types of Machine Learning Algorithms used in Software Defect Prediction Kalaivani et al. [9].

There are four commonly-used learning types associated with SDP. First, Supervised Learning (SL), during which the data sets being used contain labels to the results of the problem for each of the rows. Second, Unsupervised Learning (UL), in which the results are unknown. Khurma et al. [18] and Kumar et al. [19] found that the most popular types of learning for SDP involve SL with binary classification, whereby the input from the module is classified by the output as either being defect-free or containing defects. Figure 3 presents the types of learning algorithms used in SDP with UL and SL. The third type is semi-supervised, in which both unlabeled and labeled instances are present in the data. The fourth is reinforcement learning, in which the model is penalized or rewarded, based on the prediction made.

Dhaya Battina [4] found significant synergy between ML and DevOps to deliver software more expediently and cost-effectively. The automation of processes allows developers and operators to spend time on more important matters. The improvement of SDP, and the implementation of reliable quality assurance can greatly improve the quality of the software in a faster development cycle.

The identification of SD has been a crucial aspect in ensuring the quality and reliability of software being released for both public and private use. The increase in the complexity of software systems has made it more difficult to identify SD, which has increased the need for automation within the SDLC to handle and manage the complexity.

According to Regan et al. [20], software development in medical devices is both complex and difficult, and serious injury, or even death, can result from a defective medical device. The use of software-based medical devices within healthcare plays an important role, but managing the complexity of the devices is hard and challenging in the healthcare industry. Medical device software traceability is a critical aspect in the operational safety of the system; for example, the FDA and the European Council require a degree of traceability throughout the SDLC.

As there are stringent requirements and oversights related to the traceability of SDLC in the healthcare industry, Yarlagadda et al. [21] argued that other more typical SDLC would hinder the healthcare industry, by increasing costs and decreasing user satisfaction, being, in large part, due to the massive amounts of both data and data sources relating to medical devices, patient records, laboratory tests, and other business operations data. A solution based on DevOps, in conjunction with cloud computing, using teamwork, communication, automation, and innovative approaches, produces high reliability, faster delivery, improved collaboration among departments, and improved security and scale-ability. The use of an acceptance test suite is an integral aspect of the SDLC, especially in regulatory compliance, and development of SDP tools identifying defects is essential for quality assurance in SDLC. If SDP is successful in the identification and prediction of SD prior to releasing static software metrics, this could enable developers working within the healthcare industry to ensure quality and improve the product delivery pace.

Although there have been many ML techniques introduced and proposed for SDP, many of these models must be evaluated on different data sets and combined with other models to improve the quality of predictions. The latest research within SDP has primarily focused on the development of dimension reduction techniques in data sets and their features. These techniques reduce noise within the models, without the loss of relevant information, by considering automated Feature Selection (FS) and Feature Extraction (FE). As stated by Pandey et al. [22], over-fitting is one of the challenges that SDP attempts to overcome. Over-fitting occurs when the noise in data extensively affects the learning of the model, such that it deviates from accurately fitting with new data. In addition, there exists a gap between SDP research and its implementation. Although many researchers have presented theories and conducted experiments on model building, little effort has been expended on real-time implementation that can motivate organizations and companies to look deeper into SDLC. This inhibits a model’s advance from theory to practice, which is necessary to improve data sets, metrics, and algorithms.

### 1.2. Research Question 1 (RQ1)

What are the various ML techniques used for Software Defect Prediction? A review of recent prior research is an important aspect in limiting the scope of the experiments to be conducted, as well as in identifying ML models that perform well. A comparative analysis of different ML techniques for software defect prediction is conducted. This is covered extensively in the literature review in Section 2.

### 1.3. Research Question 2 (RQ2)

What are the appropriate ML techniques for software defect prediction based on performance indicators, such as accuracy, precision, recall, and F-measure? The identification of appropriate ML algorithms/models, or techniques, is essential to improve software defect prediction, while a literature review and experimental implementation are essential for there to be significant contributions in the research community.

### 1.4. Research Question 3 (RQ3)

What are the challenges and limitations of the implemented ML techniques while predicting software defects? Through extensive experimental results this research addressed the challenges, limitations and recommendations, associated with the tested ML techniques.

### 1.5. Research Question 4 (RQ4)

How can the prediction models be implemented into the SDLCs of healthcare applications with DevOps and how are the MLOps implemented to handle the SDLCs of models? It is important to include the means by which this research can be extracted from academia and implemented in real-time applications.

### 1.6. Our Contributions and Limitations

This research contributes in six-ways. First, we investigate and compare various ML techniques for SDP by conducting a literature review. The research was limited to studies presenting a basic understanding of the techniques. Due to there being numerous techniques reviewed, the mathematics behind each of the methods is referenced but not included. Limited insight into Deep Learning (DL) while selecting proper models, meant that implementation setup, and the additional time complexity in regard to implementation of FS and FE techniques, was previously overlooked.

Second, we analyze and recommend appropriate ML techniques for Binary Classification SDP, based on performance indicators, such as instance, accuracy, precision, recall, and F-measure, minimizing the time complexity of ML techniques due to their limited scope in the field of SDP.

Third, we investigate and compare different FE techniques, i.e., Principal Component Analysis (PCA) and Partial Least Squares Regression (PLS), in combination with FS techniques, i.e., Fisher score, Recursive Feature Elimination (RFE), and Elastic Net.

Fourth, we investigate the implementation of the SDP model in the SDLC in DevOps, as well as the implementation of models’ SDLCs in Machine Learning Operations (MLOps).

Fifth, we verify, with scikit-learn library, all the FE and FS techniques in ML algorithms in separate, and combined, fashions, i.e., Support Vector Machine, Logistic Regression, Naïve Bayes, K-Nearest Neighbor, Multilayer Perceptron, Decision Tree, and ensemble learning methods, Bootstrap Aggregation (Bagging), Adaptive Boosting (AdaBoost), Extreme Gradient Boosting, Random Forest, and Generalized Stacking.

Sixth, an extensive experimental setup was built by considering various large data set repositories, i.e., PROMISE and NASA MDP in Python with the PyCharm development environment and the scikit-learn library. Then, we classify, categorize, and recommend SDPs and present their limitations. Due to the fact that no open source SD data set related to healthcare industrial applications or devices could be found, the well-established SDP data sets, PROMISE and NASA MDP, were used. We believe it is reasonable to adopt the static metric features of the source code, extracted regardless of domain specifications, in the experiment for healthcare use.

The organization of the paper is as follows. Section 2 contains the materials and methods with a detailed literature review, and experimental setup. In Section 3 the results are presented from the experiment found in the prior section. Section 4 presents the discussion of the results. The challenges, limitations, and recommendations for future work can be found in Section 5. Within Section 6 a healthcare use case is presented. Finally, the paper is concluded in Section 7.

## 2. Materials and Methods

The methodology used in the research into SDP included a literature review and an experimental setup and most of the research ethics were followed.

### 2.1. Literature Review

To contribute meaningful research in SDP using ML techniques it is necessary to consider exclusion–inclusion criteria, by selecting peer-reviewed scientific articles from both journals and conference proceedings. The selected articles were published within the last three to four years. Research articles were considered based on fundamental knowledge in the field, regulations, insights, processes or importance of specific techniques.

The selected literature was obtained from Kristianstad University’s research portal, Google Scholar, IEEE Xplore, Springer database, Science Direct, and Research Gate. Due to the numerous ways of data processing available, as discussed by Akimova et al. [15], no specific benchmark was considered. Only the analysis of quantitative data was considered during our experimental setup. The quantitative analysis was performed in alignment with text, and evaluation of ML techniques and models. The intensive literature review provides better insight into previously used algorithms and techniques and broadens knowledge to answer our RQ1, RQ2 and RQ3, requiring both literature study and experimental results.

#### 2.1.1. Data Sets and Cleaning

As stated by Kumar et al. [19], data collection and data pre-processing are crucial aspects in the ML model building process. Data sets adopted for the development of the statistical models that had any data inconsistencies or errors observed during the process, were properly assessed during the data pre-processing operation. The model was then re-evaluated to assess its validity. Dhaya Battina [4] argued that it is necessary to properly validate data while working with ML models.

The most widely used data sets in SDP are the Predictor Models in Software Engineering (PROMISE), and NASA Metrics Data Program (MDP)m according to Saharudin et al. [6]. It was observed that 43.3% of each adopted data set was considered in research experiments, while in total usage, 86.6% was due to the open-source nature. Akimova et al. [15] pointed pout that the difficulties associated with SDP are class imbalances within the data sets from real-world projects, and the lack of context between closely-related classes.

Shepperd et al. [23] compared NASA MDP data sets and found that there were missing values, inconsistencies, implausible values, and conflicting feature values. The pre-processing of the data is a significant step and modifications must be made based on the needs of the model. The data pre-processing should handle missing information and inconsistencies, as well as having some sort of scaling and normalization. Mehta et al. [13] pointed out that if features are scaled to different measures, it can result in misrepresentation of the model.

#### 2.1.2. Quantification Metrics

Performance metrics are important indicators to measure and assess the quality of ML models. Saharudin et al. [6] found that, for SDP, the most widely included types of numerical quantification measurements are Area Under Curve (AUC), based on the results of the Receiver Operating Characteristic (ROC) curve, hqving 56.7%, Recall, with 46.7%, F-Measure/F1-Measure, with 36.7%, Precision, with 30%, Accuracy, with 26.7%, and Other numerical measurements with 76.7%.

#### 2.1.3. Data Reduction, Transformation, and Selection

Song et al. [24] and Mehta et al. [13] showed that FE reduces the dimensionality of features by transforming the data set through axis rotation into a new subset of components. The goal was to obtain new components without loss of relevant information during standardization and splitting of data into training and testing sets.

Song et al. [24] proposed a model that incorporated PCA, in combination with an optimization algorithm, known as Cuckoo Search, to increase prediction efficiency by optimizing weights and thresholds with the help of the Elman Neural Network (ENN). Making a comparison of the proposed model to traditional ENNs and to Back Propagation Neural Network (BPNN), an experiment was conducted, considering the PROMISE data set repository. They observed that ENN performed better than BPNN, while their proposed model increased the performance of the ENN.

A model proposed by Pandey et al. [22], to handle the class imbalance and over-fitting challenges within SDP, used an Extreme Learning Machine (ELM), a feed forward Neural Network that contains a single hidden layer that generates weights based on the analytical results of randomly chosen hidden units. The learning algorithm implemented Kernel–PCA (KPCA), a non-linear form of PCA that uses an orthogonal transformation for data dimension reduction. The two adopted data sets were PROMISE (Ant, Arc, Camel, Ivy, Jedit, Log4j, Poi, Prop, Redaktor, Synapse, Tomcat, Velocity, Xalan, and Xerces) and NASA MDP (CM1, JM1, KC1, KC2, KC3, MC1, MC2, and PC1). To handle the class imbalance of each of the data sets, the Synthetic Minority Oversampling Technique (SMOTE) was implemented. Logistic Regression (LR), Multilayer Perceptron (MLP), Naïve Bayes (NB), and Support Vector Machine (SVM) were compared.

Massoudi et al. [25] adopted CM1, JM1, KC1, KC2, and PC1 from the NASA MDP repository data sets, with Artificial Neural Network (ANN) and Decision Tree (DT) as the learning algorithms, and theoretically compared with PCA and KPCA. In addition, they found that each technique performed well on different data sets with PCA–DT performing better than KPCA-DT, but significant improvement was observed from PCA–ANN over KPCA–ANN with the PC1 data set.

**Figure 4:**
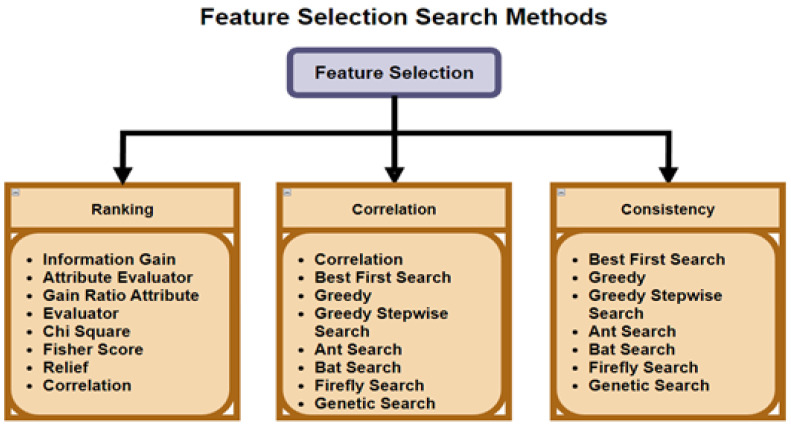
Feature Selection Search Methods Hà et al. [27].

FS reduces a data set into a subset of important features, as stated by Shamsuddeen et al. [26]. FS is used for high dimensional problems, wherein the model is over-fitting, due to noise within the feature-set, but has been used for the general purpose of eliminating less meaningful features. The selection process uses a predetermined measurement to assess the features by properly separating classes or analyzing classification performance. As stated by Mehta et al. [13], FS is a promising technique, which not only reduces the complexity of the ML algorithm, but also improves accuracy. Figure 4 shows the different search and selection methods that can be deployed within FS algorithms.

Shamsuddeen et al. [26] and Mehta et al. [13] presented three categories of FS techniques: Filter, Wrapper and embedded.

**Figure 5:**
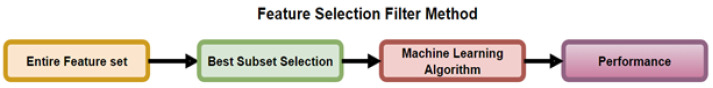
Process of the Feature Selection Filter Method Mehta et al. [13].

The Filter method is presented in Figure 5, where the subset selection is independent from the ML algorithm. This implementation creates a feature subset that is largely based on the output class (suitable for use in prediction with ML algorithms). The output of the subset selection is used in the ML algorithm to predict and evaluate performance.

**Figure 6:**
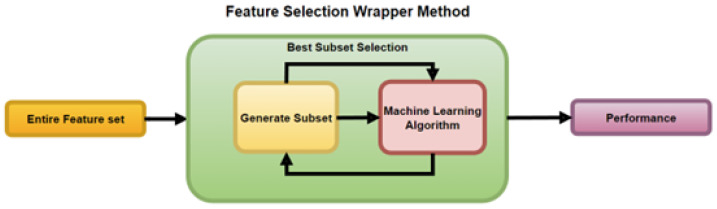
Process of the Feature Selection Wrapper Method Mehta et al. [13] and Shamsuddeen et al. [26].

The Wrapper method is depicted in Figure 6. It works by wrapping the ML algorithm, using it within its subset selection of the entire feature set for training the model. The selection algorithm goes through a continuous search using the learning algorithm, and its useful results lead to the addition and removal of features.

**Figure 7:**
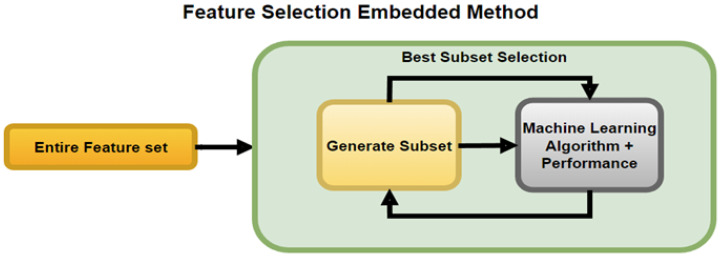
Process of the Feature Selection Embedded Method Shamsuddeen et al. [26].

The Embedded method is a hybrid of the filter method and wrapper method, as depicted in Figure 7. Embedded methods make a best initial subset to improve the performance of an internal learning algorithm and the prediction capability of models.

As stated by Khurma et al. [18], FS consists of searching and evaluating the sub-process. The search in the FS process can implement many different methods, such as brute force method (i.e., it traverses through subsets of all features with more time complexity), and the meta-heuristic method (i.e., swarm intelligence can give random solutions that produce good results in a shorter time).

Hà et al. [27] conducted an experiment on the CM1 and the MW1 MDP data sets by comparing filter methods in terms of Fisher score, Gain Ratio, Information Gain, Relief and Chi-Square. The algorithms used were K-Nearest Neighbor (KNN), DT, Random Forest (RF), NB, and MLP. The top three results for MLP (Fisher Score, Information Gain, and Chi-Square), NB (Fisher Score, Information Gain, and Chi-Square), RF (Fisher score, Gain Ratio, and Baseline), DT (Fisher Score, Gain Ratio, and Baseline) and KNN (Information Gain, Gain Ratio, and Baseline) were calculated with the CM1 data set.

Moth Flame Optimization (MFO) is an optimization algorithm that uses the swarm intelligence model based on a moth’s spiral flight path around a source of light in the FS process. The flame and the moth’s random flight path around the flame are considered to be potential solutions during the search process. Khurma et al. [18] proposed a model based on MFO, the Island Binary Moth Flame Optimization (IsBMFO), to produce many MFO models to conduct the same search process, called islands.

Each of these islands has a predetermined number of iterations, and the results of individual islands are shared among the others. An experiment was conducted on the NB, KNN and SVM ML algorithms. Their results showed good results from, and better improvements with, the FS method and the IsBMFO–FS method, respectively, in all the models. Analysis showed that SVM outperformed all the other methods, because the average feature reduction ratio for all the data sets was 62% and the average precision improved from 30% to 70%.

Least Absolute Shrinkage and Selection Operator (LASSO), according to Wang et al. [5], Mehta et al. [13] and Osman et al. [28], is an embedded method that uses Linear Regression with L1 regularization (in which the features are reduced by adding a penalty to the loss function) to calculate the minimum squared sum of coefficients. If the identified features have a value less than the threshold values then they can be considered as zero and discarded.

Wang et al. [5] addressed the fact that SVM adopts min–max for data normalization prior to FS and then the wrapped SVM algorithm (which uses an RBF kernel) with the Least Absolute Shrinkage and Selection Operator (LASSO). They performed experiments by using ten-fold cross-validation on the data sets against Fisher Linear Discriminant Analysis (LDA), Cluster Analysis (CA), Back Propagation Neural Network (BPNN), SVM and LR. The results of Baseline (original) and the LASSO method of SVM showed a minimum improvement of 6% in accuracy, precision, recall, and F-measure. This was carried-out with the remaining algorithms where accuracy increased by a minimum of 8%, precision slightly increased to 2% and F1-Measure or F-Measure revealed an improvement of 4% with the CM1 data set.

Osman et al. [28] conducted an experiment using embedded regularization techniques with the goal of increasing accuracy in SDP. The implemented methods were Ridge Regression, an embedded method which adopts a linear regression function, known as Least Squares Error (LSE), or L2 regularization (a modified loss function where the penalty is the square value of the coefficient). Elastic Net is another implemented embedded method (that adopts a quadratic expression in the penalty function), utilizing both L1 and L2 regularization. The results showed that both L1 and L2 regularization regression (as the FS techniques) improved the performance of the models (in terms of root mean squared error as the quantification metric) up to 50%.

Mehta et al. [13] used LASSO in the experimental setup with several other FS techniques in conjunction with PLS, Pearson’s Correlation (i.e., a filter method that takes the linear dependence measure between features and selects the features with low inter-correlation at input class and high correlation with the output class), Boruta (i.e., a wrapper method using a RF Classifier to train an extended data set with created shadow features, to evaluate their importance ), and RFE (i.e., a wrapper-based method that uses a greedy algorithm to rank the least relevant features). They conducted an experiment on the MDP repository data sets (CM1, PC1, KC1, and KC2). The results revealed that RFE performed better with all data sets when paired with RF, GB, and DT, while the best performance over all the data sets was observed when one data set was paired with Stacking, Adaptive Boosting (AdaBoost), and eXtreme Gradient Boosting (XGBoost). Lasso performed better when paired with the Kernel–SVM, except in regard to the KC2 data set. LR showed no conclusive evidence in terms of performance analysis and differentiation of one regression FS algorithm over another.

#### 2.1.4. Ensemble Learning Methods

Ensemble Learning methods increase the accuracy of the prediction model by combining the output of multiple classifiers, as presented in Mehta et al. [13]. The ensemble learning is implemented by either using one Baseline model several times on different subsets of data, or various different models on the same data set.

**Figure 8:**
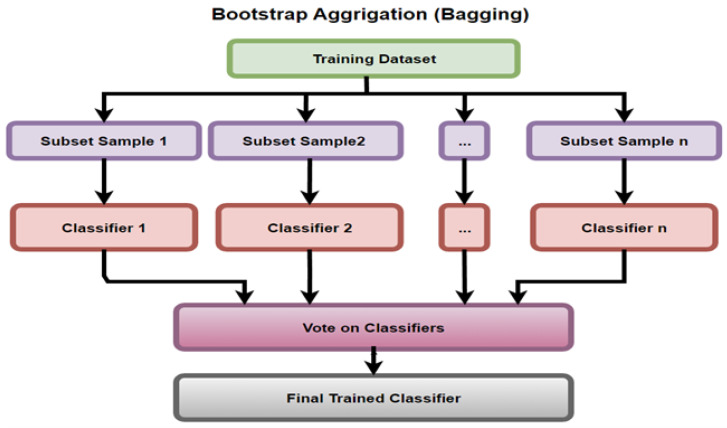
Bootstrap Aggregation A.K.A Bagging Diagram.

Kumar et al. [19] proposed Bagging to improve performance by reducing over-fitting of the model. The PROMISE data sets (i.e., Ant 1.7) were considered for three main models, RF (accuracy 89.4%), SVM (accuracy 94.9% ), and Bagging (accuracy 96.24%). For the Jedit 4.0 data set, the three models, from the aspect of accuracy were RF (91.5% ), SVM (95.1%) and Bagging (96.7%), respectively. For the Camel 1.4 data set, the top three models for accuracy measurement were DT (86.81%), SVM (95.87% ) and Bagging (95.98%). Bagging showed better performance in terms of accuracy, F-measure, AUC–ROC, and precision than the other models. Bagging is presented in Figure 8 and divides the training data set into n subsets of samples (which are trained on n classifiers) and are then selected by committee for the final trained classifier.

Yalçıner et al. [29] compared MLP, RBF, SVM, Bagging, RF, NB, and Multi-nomial NB on MDP data sets (i.e., PC1, CM1, KC1, and KC2). According to their analysis RF and Bagging performed the best, and the results of the models were validated using ten-Fold Cross Validation. The results divided by data set showed the best performing models on the PC1 data set were MLP (accuracy of 93%, precision of 92%, recall of 93%, and F-measure of 91%), and RF (accuracy of 93%, precision of 92%, recall of 93%, and F-measure of 92%), and the best performer was Bagging (accuracy of 94%, precision of 93%, recall of 94%, and F-measure of 92%). With the CM1 data set the best performing models were RBF (accuracy of 89%, precision of 81%, recall of 89%, and F-measure of 85%), and SVM (accuracy of 89%, precision of 81%, recall of 89%, and F-measure of 85%), and, again, the best performer was Bagging (accuracy of 89%, precision of 81%, recall of 89%, and F-measure of 85%).

The KC1 data set was tested with the three best performing models and the results were: MLP (accuracy of 86%, precision of 83%, recall of 86%, and F-measure of 82%), Bagging (accuracy of 86%, precision of 83%, recall of 86%, and F-measure of 83%), and RF (accuracy of 86%, precision of 84%, recall of 86%, and F-measure of 84%). Similarly, with the KC2 data set the results of the three best performing models were: RBF (accuracy of 83%, precision of 82%, recall of 83%, and F-measure of 82%) and and Bagging (accuracy of 84%, precision of 83%, recall of 84%, and F-measure of 83%).

Khan et al. [17] compared SVM, J48 DT, RF, KNN, NB, MLP, RBF, Hidden Markov Model, Credal DT, and Average One Dependency Estimator (A1DE). They adopted CM1, JM1, KC2, KC3, and MC1 datasets from the NASA MDP, and an additional two data sets, AR1 and AR3, from the AR repository were considered. They found that RF outperformed all models in terms of accuracy and recall performance metrics over all the data sets.

Mehta et al. [13] analyzed the best performing FS technique, RFE, from a prior experiment and then implemented Z-Score standardization for scaling, with PLS FE in combination. Their experimental setup also considered SMOTE on the data to handle the class imbalance problem. The adopted algorithms were MLP, LR, DT, SVM, and KNN, along with the ensemble learning methods i.e., Extra Trees (ET), RF, Bagging, AdaBoost, Gradient Boosting (GB), XGBoost and Stacked Generalization (Stacking). The results revealed that XGBoost, and the Stacking models performed better than other models, with consistent scores above 94% on all quantitative metrics for all the data sets.

#### 2.1.5. Machine Learning Operations

According to Dhaya Battina [4], the best practice for any organization to develop and deploy ML models into the SDLC is to build a Continuous Integration (CI)/ Continuous Development (CD) pipeline. This is to handle the ability to scale out the models as the infrastructure evolves, and, also, to handle the ever-changing ML model for accurate predictions. i.e., MLOps, as presented by Symeonidis et al. [30]. The MLOps uses a collection of tools and processes for the deployment of the ML models into production.

**Figure 9:**
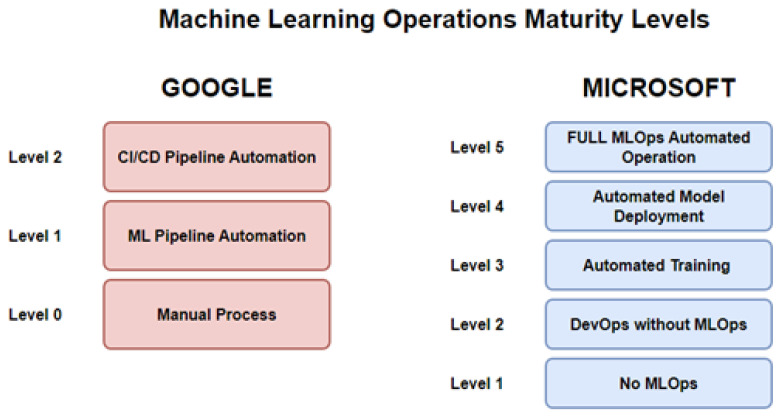
Machine Learning Operations Maturity Levels.

The building of a pipeline is a task often done sequentially, due to the strenuous nature of the task, as stated by Ruf et al. [11]. The goal of MLOps is to automate, manage, and speed up the ML model operation by integrating the DevOps process. The maturity level of MLOps implementation is classified into three and five categories by Google (GGL level 0: manual implementation, GGL level 1: an automated pipeline process of building and selecting models but deployment itself remains manual, GGL level 2: a full CI/CD pipeline) and Microsoft (MS level 1: No MLOps, MS level 2: implementation of DevOps but no MLOps, MS level 3: automated training of the model is implemented, MS level 4: the model is deployed autonomously, and MS level 5: the operations are fully through MLOps), respectively, as depicted in Figure 9 and described by Symeonidis et al. [30].

Ruf et al. [11] presented the MLOps workflow as consisting of different phases. First is the project requirement engineering phase where data-scientists, software engineers, and domain experts properly define the problem, and then determine the project requirements. Second, the data management phase, in which data the scientists and domain experts are responsible for validating usability and data quality, so that problems are discovered as early as possible in terms of completeness, accuracy, structures, and format of the data. Third, the ML preparation phase, in which the acquisition of the data, the cleaning, and labeling is conducted. As the inputs may change over time, this phase tracks the evolution of the data. Fourth, the ML training phase, which has been heavily covered, and the fifth being the deployment phase, where software engineers integrate the model into the application depending on the function of the model, either by embedding it or through a REST API using the model as a service, and operations deploy and monitor the model and application.

**Figure 10:**
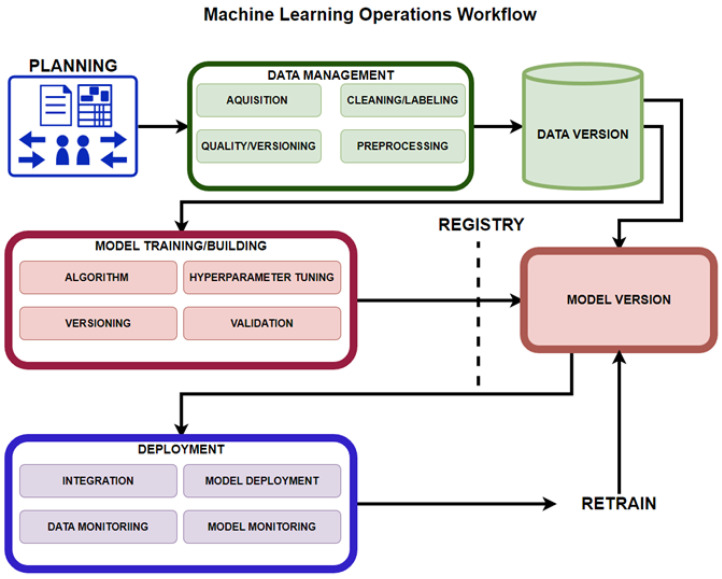
Machine Learning Operations Workflow.

Symeonidis et al. [30] presented three fundamental pipelines: the data manipulation pipeline, model creation pipeline, and the deployment pipeline. Figure 10 presents all these pipelines in the overall MLOps workflow, where planning takes place, followed by data manipulation or management, in which the data is validated, cleaned, labeled, versioned and pre-processed into a database.

Symeonidis et al. [30] concluded that a fully mature MLOps system is the most efficient way to incorporate ML models into production. The MLOps implementation can be challenging, due to the wide variety of tools, limitations, and use-cases for the model. Ruf et al. [11] argued that there is no single tool for fully-automated MLOps workflow implementation, and the availability of several tools showed overlapping features which increased redundancy.

#### 2.1.6. Lessons Learned from the Literature Study

In the literature review section, it was summarized that either FE or FS can improve the quantitative performance within a model, although it is difficult to say which provides the better performance with different algorithms, models and data sets. Prior research on both FE and FS, in general, reduced the data set to an arbitrary number while comparing models for noise reduction. In reality, changing the data sets changes the performance of FE, FS, learning algorithms and models.

Regarding the individual FE techniques, PLS is considered as more of a potential candidate, due to its supervised nature, than the PCA; although little is known about how each data set handles different techniques. In addition, the FE and FS techniques behave differently with different base ML algorithms, for instance, LASSO–SVM shows organized functionality. Regarding the individual FS techniques, RFE is a very effective wrapper method, although more testing should be performed to compare with other FS methods.

Ensemble techniques, such as XGBoost and Stacking, reveal stable, and similar, performances with little variation in results over a wide range of data sets. The Filter methods showed that Fisher score and Gain Ratio were consistent across most of the algorithms over CM1 data sets.

Other data sets, i.e., MW1, do not show any significant variation, even with all considered features, so more tests on other data sets may be needed.

### 2.2. Experimental Setup

This section compares different FE techniques, namely, PCA and PLS, in combination with FS techniques, i.e., Fisher Score, RFE, and Elastic Net. These techniques are then applied to the Base (i.e., Baseline or original) ML algorithms, such as SVM, LR, NB, KNN, MLP, and DT, and ensemble learning methods i.e., Bagging, AdaBoost, XGBoost, RF, and Stacking. These Base (i.e., Baseline or original) ML algorithms, when tested separately, act as a baseline that can be compared to application of the FE and FS techniques.

The experiment was conducted on a Windows 10 Operating System, with an AMD 5900x CPU 12 cores 24 threads 4300MHz clock, and a Nvidia RTX 3070 GPU. The programming language used in the development of the ML models was python 3.9, the development environment was PyCharm 2022.1, and the ML techniques were implemented using the scikit-learn 1.1.1 as well as the Pandas libraries.

**Figure 11:**
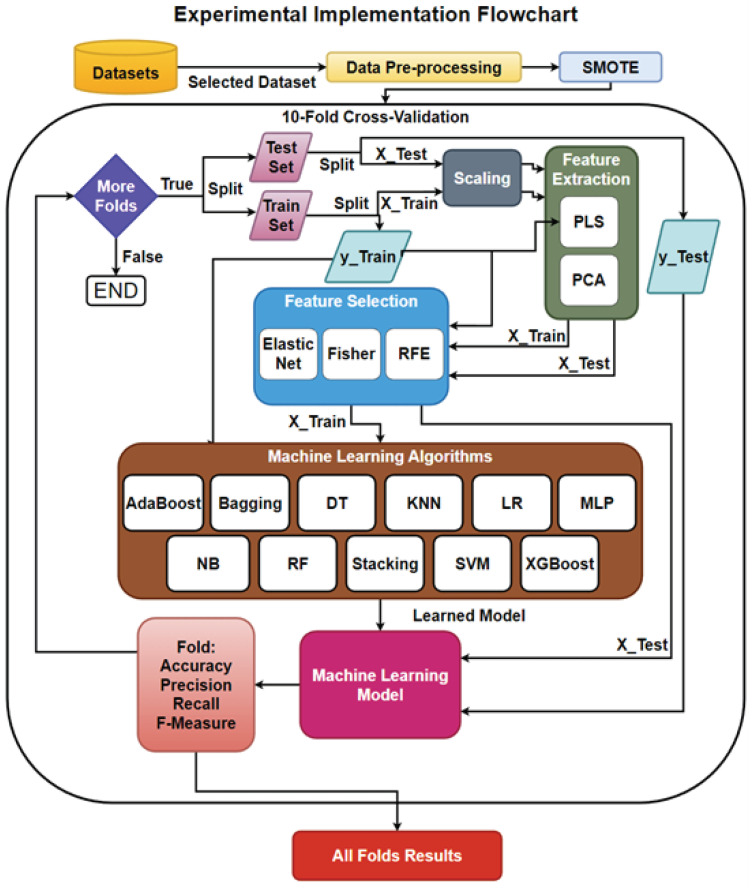
The Experimental Setup for the Model Building Process.

Figure 11 presents the implementation of the models. The data set was pre-processed by handling missing values, duplicates, and class imbalance (by conducting SMOTE). Then, that data was passed into ten-fold cross validation where, at each iteration, a new portion of the data was split into train/test sets (i.e., the sets themselves were split, based on X being the features of the set and y the target or labels). Then, FE and FS were applied to both the X-train and y-train in order to train these techniques to handle the data in the reduction and the X-test applied the trained technique to the set. The techniques returned a transformed X-train and X-test. The X-train and y-train were used to train the ML model. The learned model then took each instance of the X-test set for predictions to compare with the y-test, where the results were stored in a confusion matrix.

The accuracy, precision, recall, and f-measure were calculated for the fold and then stored. The final results were obtained after the last iteration of the cross validation, where the average of metrics was stored to evaluate the model.

The Algorithm 1 demonstrates how the implementation would handle working with the base model separately or in combination with the FE and FS methods (i.e., the base model being tested separately without FE or FS applied is a baseline for comparison).

In the beginning, if SMOTE was being used in a particular experiment then re-sampling took place using the X and y instances from the tested data set. If FE, FS or both were being utilized in the experiment, the best k value was found for each either in combination or separately. Next, the cross-validator at each iteration provided the index for both the tested and trained sets. At each iteration, standardization occurred on the splits to provide a better distribution of the features, and, then, depending on whether any feature reduction took place or not. Finally, the model was trained then tested to calculate the performance metrics to be added to the list during each iteration.
**Algorithm 1** Experiment Implementation1:Input X,y,model,cv,FE,FS // X instances and features, y instance targets, cv cross-validator, FE boolean, FS boolean2:Output Accuracy,Precision,F−Measure,Recall,kFE,kFS // List results for each fold3:Accuracy,Precision,F−Measure,Recall = [] // Each iteration append accuracy, precision, f-measure, recall to lists4:**if** smote **then**5:    X,y = SMOTE(X,y) // create balanced data6:**end if**7:**if** FE and FS **then**8:    kFE,kFS = BestKFE&KFS(X,y,model) // Get best K for both FE and FS9:**else if**10:    **if thenthen**FE11:        kFE = BestKFE(X,y,model) // Get best K for FE12:    **else if** FS **then**13:        kFS = BestKFS(X,y,model) // Get best K for FS14:    **end if**15:**end if**16:**for** $train_{i}$, $test_{i}$ in cv.split(X) **do**17:    XTrain,XTest,yTrain,yTest = Split($X,y,train_{i},test_{i}$)18:    XTrain,XTest = Standardization(XTrain,XTest)19:    **if** FE and FS **then**20:        XTrain,XTest = FEandFSMethods(XTrain,yTrain,XTest,kFE,kFS) // Train and transform21:    **else if**22:        **if thenthen**FE23:           XTrain,XTest = FEMethod(XTrain,yTrain,XTest,kFE) // Train and transform24:        **else if** FS **then**25:           XTrain,XTest = FSMethod(XTrain,ytrain,XTest,kFS) // Train and transform26:        **end if**27:    **end if**28:    accuracy,precision,f−Measure,recall = modelMethods(XTrain,yTrain,XTest,yTest) // Train and test29:**end for**

#### 2.2.1. Data Sets

The extensive experimental setup conducted considered NASA Metrics Data Program repository, consisting of data sets (i.e., CM1, a NASA spacecraft instrument system written in C, JM1 a program written in C that is a real-time predictive ground system, KC1 a C++ system that manages the storage system of ground data, and KC3, another part of the KC1 project that is in the Java language). Less investigation went into the documentation of the MC1 dataset (which was done in both the C and C++ language), and MC2 data set (written in the C language). There is less information about the MW1 project being written in C instead of other languages. The PC3, and PC4 languages are unknown, as well as the projects themselves, and are apart from PC1, PC2, and PC5 projects with C and functions for orbiting satellite flight software. PC5 is in the language of the same project. The MDP data set was constructed through NASA, with contributors on the specific data sets projected being Tim Menzies, Mike Chapman, and Pat Callis. The conducted extensive experimental setup considers NASA Metrics Data Program repository consisting of the data sets (i.e., CM1, a NASA spacecraft instrument system written in C, JM1 a program written in C that is a real-time predictive ground system, KC1 a C++ system that manages the storage system of ground data, and KC3 another part of the KC1 project that is in the Java language). Less was investigated on the documentation of the MC1 dataset (which was done in both the C and C++ language),and MC2 data set ( written in the C language). Also less information was presented that MW1 project is written in C instead of other languages. The PC3, and PC4 languages are unknown as well as the projects themselves they are apart from PC1, PC2 (written in C language) for orbiting satellite flight software, and PC5 project (with C++ functions). The MDP data set was constructed with the help of NASA contributors i.e., Tim Menzies, Mike Chapman, and Pat Callis.

In addition, the following PROMISE repository data sets, with open-source Java projects were used: Ant 1.7, a build tool; Camel 1.6, a Spring-based Enterprise Integration tool; Ivy 2.0, a dependency management tool; Jedit 4.3, a syntax tool; Log4j 1.2, a tool that logs changes of an application; Lucene 2.4, a search engine tool; Poi 3.0, an API that allows for the manipulation of Office files in Java; Synapse 1.2, an Enterprise Service Bus and mediation engine; Velocity 1.6, a template engine; Xalan 2.4, a XML to HTML parser, and Xerces 2.0.0, an XML parser written in C++.

The NASA MDP and PROMISE repositories are comprised of many other data sets, not only for software defect prediction, but also for effort prediction, text mining, and model-based software engineering, as presented by Cheikhi et al. [31]. The missing values were handled by inserting the mean of the features value. The data sets were selected on the basis of their prevalent use within SDP, and the features supplied from these data sets contained real-world data from different types of projects, different programming languages and different metrics. The data sets found in Table 4 show the number of features, instances, and instance makeup can be seen these data sets were chosen due to their availability and use in prior research. Although we did not find any data set on healthcare projects with a defect repository, we adopted some data sets with similar features, resembling the healthcare use case. Incorporating a larger array of data sets within the experimental setup helped to identify any shortcomings affecting the model, as well as showing whether it could be relevant to all programming languages, thus providing insight and inter-linking the models for Software Defect Prediction in healthcare applications.

#### 2.2.2. Scaling and Standardization

Relevant non-numerical features are not prevalent in both the PROMISE and the MDP data sets. Although encoding techniques, like One-Hot Encoding, were not required for the data set, the classifications of yes, no, true, and false of the output class were converted to 1 for true or 0 for false by using a label encoder. The standardization technique that was employed on the data sets was Z-Score or zero mean normalization, which converted the feature values to a common scale in which the mean was equal to 0 and the standard deviation was 1. If the values of the z-score were outside of the range of −3 and 3 then the score would be considered unusual or an outlier, and if the score resulted in a positive value that was below 3 then the z-score would be defined as above average, and with an average value of 0. Similarly, if the z-score was a negative value greater than −3 then it would be defined as below average.

#### 2.2.3. Feature Extraction

The FE techniques considered to offer better performance were implemented for the ML models, i.e., PCA and PLS. As the PLS technique also takes into consideration the label or output class to the time complexity, it should be compared with the PCA to evaluate efficiency. For each of the future algorithms and data sets, the required list of components was considered and compared with the brute force algorithm for performance evaluation to find the best features.

PCA is a widely adopted FE technique, and an unsupervised ML method for data dimensions reduction, also used for other FE techniques, such as PLS Hervé Abdi [32]. The main goal is to capture frequent variation in a smaller dimension by combining the features to a new data set of Eigen vectors or principal components, with the help of axis rotation. The process began as described in Song et al. [24] standardization, with mean used for scaling up the feature values to a common range during the implementation, unlike prior standardization with Z-Score. The covariance was found by calculating the variance of the features, and the covariance matrix was formed on the basis of number of features. The Eigen values were calculated using the covariance matrix with the identity matrix value to find the corresponding Eigen vectors as the principal components.

In PLS, as described in both Hervé Abdi [32] and Mehta et al. [13], the principal components developed from the PCA technique do not need to get relevant information for selection due to their autonomous nature. PLS can be described as a supervised version of PCA used to predict the classifier. In this experiment, the components were extracted and used with the learning algorithms being tested, instead of making a prediction from the regression algorithm. PLS used least squares regression as an additional step to PCA that decomposed the matrix to predict the classifier. Then, the maximizing of the covariance was done by creating a linear combination column of the features, resulting in a smaller set of non-correlated features.

#### 2.2.4. Feature Selection

The FS techniques, being a wrapper method, a filter method, and an embedded method, were adopted in the experimental setup and compared with the PLS, PCA and FE techniques.

The FS techniques were chosen, based on their performance during the comparative analysis. For instance, the RFE technique was suitable for the wrapper method. The filter method was used as the Fisher Score (because of its meaningful feature selection traits and more consistent performance across several different algorithms). The Elastic Net technique was chosen as an embedded method by adopting L1 and L2 regularization for better evaluation of the models. The FS techniques adopt the similar brute force algorithm in identifying the number of features for Fisher Score, and RFE. Fisher Score, as the name implies, implements the fisher score algorithm to rank the features independently and then selects several top features among the several input features to select a suitable algorithm, Hà et al. [27].

RFE as the FS technique uses a greedy algorithm for the ranking of the performance of the classification accuracy of the previous iteration. The features that are the least relevant are discarded from the model and the process continues till all the relevant features are separated as the reduced subset, as addressed by Mehta et al. [13].

Elastic Net, as described in Osman et al. [28] and Mehta et al. [13], uses Ridge or L2 regression to establish the initial coefficients then shrinks these using LASSO or L1 regression.

#### 2.2.5. Learning Algorithms

MLP, as described by Yalçıner et al. [29] and Khan et al. [17], is a neural network, consisting of an input layer, hidden layers, and one output layer. The hidden layers and output layers act as classifiers, passing the weights of the nodes to the others, and are updated with the help of the back-propagation training technique.

SVM can be used in both regression and classification problems, as addressed in Wang et al. [5] and Yalçıner et al. [29]. In a classification problem, a separation line is known as a hyper plane; which is defined by the support vectors to divide the classes. Many different kernels can be implemented to perform various mathematical functions in the SVM algorithm.

LR is an extension of linear regression which not only calculates a best fitting line, but adopts values from coefficients and calculates the output for a binary prediction with the help of a logistic function algorithm. Further details can be read in Mehta et al. [13].

Note that, according to both Khurma et al. [18] and Anjali Munde [33], an algorithm that is known as naïve assumes that every input variable is independent and creates a model directly from the data used by Bayes Theorem to predict new inputs.

KNN, as found by Khurma et al. [18] and Khan et al. [17], is an algorithm that follows the same strategy as used by NB to adopt the data set directly as a model. The KNN performs prediction directly from the k-inputs, which are the most similar instances represented in the model. Euclidian’s distance is one of the most widely used algorithms to identify the nearest k-instances, and, due to its ease of implementation, only the closest values are combined to the intended class label.

DT, as defined in Anjali Munde [33] and Mehta et al. [13], is another type of prediction algorithm that can be used in either classification or regression. Starting at the root node, the input is used to traverse down the tree. Decisions are made based on the values, and the class or value is determined after arrival at the leaf node.

Bagging, as defined by both Kumar et al. [19] and Yalçıner et al. [29], is an ensemble learning method that takes many samples of the data set and constructs models for each of the samples. The predictions from each of the models are considered, then suitable candidates are predicted on the basis of voting by a committee.

RF, as defined by Khan et al. [17] and Anjali Munde-ICTIS [33], is one of the most widely adopted techniques, as it performs well on both classification and regression problems. The algorithm builds a vast amount of DT models by randomly using samples of the chosen data set. The final prediction is made on the basis of a voting process in favor of the desired model.

**Figure 12:**
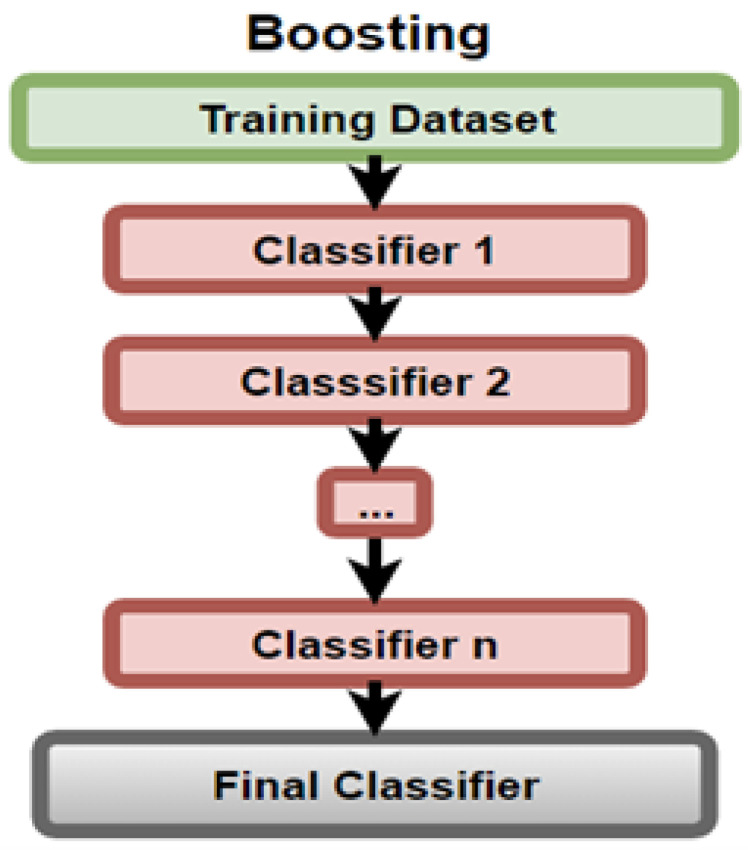
Boosting Diagram.

AdaBoost, as found by Mehta et al. [13], is a technique that uses weak DT classifiers to train strong ones by overcoming the weaknesses of the previous trees, as shown in Figure 12.

XGBoost, as found in Mehta et al. [13], uses the gradient boosting algorithm to avoid bias and over-fitting with the help of pruning and other processes unavailable to other boosting models. Gradient boosting, like other boosting methods. combines weak learners for output prediction, and, in addition, uses gradient descent to reduce the errors in the other models.

**Figure 13:**
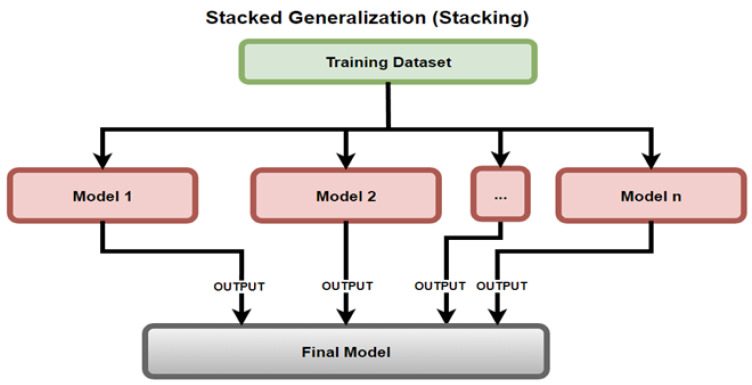
Stacked Generalization (Stacking).

Stacking, as defined by Mehta et al. [13], uses an ensemble of models by consolidating the predictions of previous models to train a new model, as depicted in Figure 13. The models used within this experimental set up were level 0 LR, KNN, DT, SVM, NB, RF, and level 1 LR.

#### 2.2.6. Quantification Metrics

Quantification metrics, such as Accuracy, Precision, Recall and the F-Measure were considered to analyze the results. In the SDP research the results are obtained and presented in percentage, rather than in ratio, so the calculations were modified accordingly. As AUC–ROC is generally used for a graphical representation it was not implemented for the analysis of the results. The calculation and use of these quantification metrics presented below followed from Anjali Munde-ICTIS [33] and Saharudin et al. [6].

The confusion matrix in Table 5 uses the terms reported vs. actual results. Each of the rows corresponds to the reported class as an outcome of the input with defective or non-defective classes, while the columns indicate the actual class outcome of the input in the experimental setup. Once the corresponding results are determined they can be recorded as True Positive (TP), where both actual and reported are true, False Positive(FP), where reported is true and actual is false, False Negative(FN), where the reported is false but the actual is true, and True Negative(TN), where both reported and actual are false.

Accuracy is measured and determined based on the percentage of correctly identified and classified defects in the testing of the model from an overall perspective as given in Equation (1).

(1) $$accuracy=100\times \frac{TN+TP}{TP+FP+TN+FN}$$

Precision is the measure of the percentage of correctly reported positives that are true positives, as shown in Equation (2).

(2) $$precision=100\times \frac{TP}{TP+FP}$$

Recall is the measure of the percentage of reported positives of all the true positives within the entire data set, as presented in Equation (3).

(3) $$recall=100\times \frac{TP}{FN+TP}$$

F1 or F-measure can be used to evaluate both recall and precision in a single measurement by calculating the mean between them, as revealed in Equation (4).

(4) $$f-measure=100\times \frac{precision\times recall\times 2}{precision+recall}$$

## 3. Results

**Figure 14:**
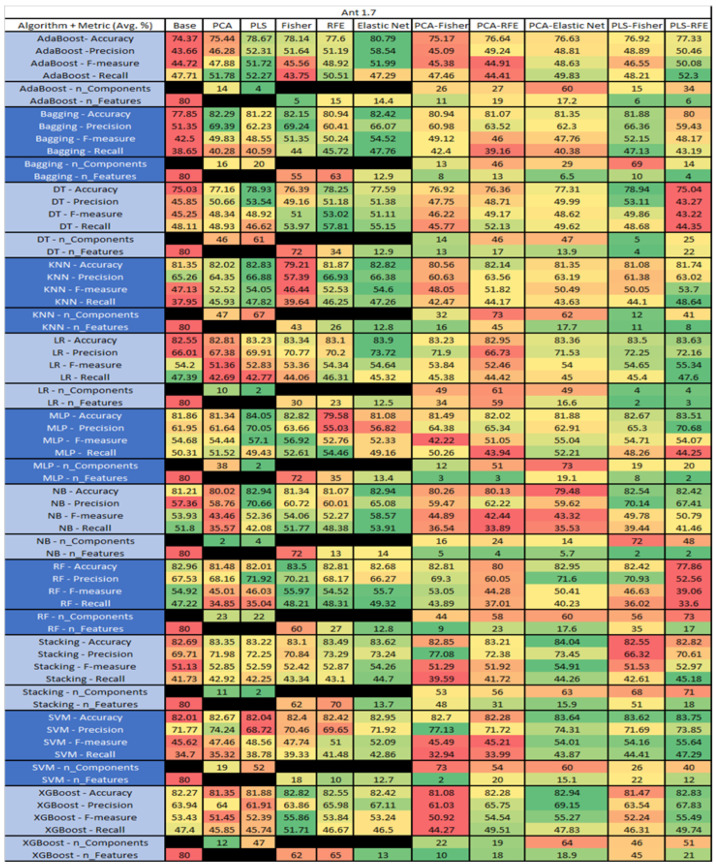
Ant 1.7 PROMISE Results Data Matrix.

**Figure 15:**
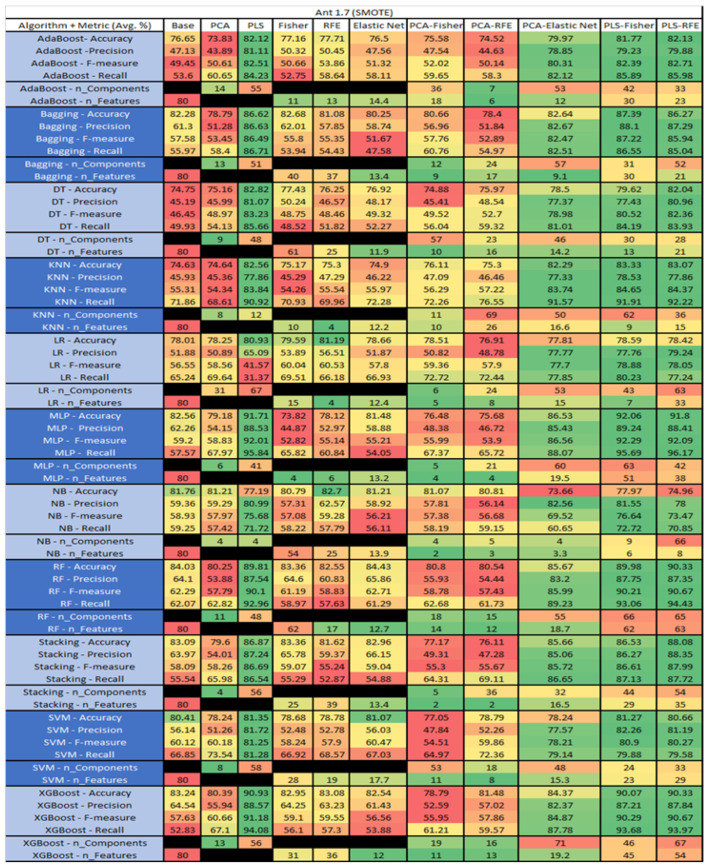
Ant 1.7 with SMOTE PROMISE Results Data Matrix.

**Figure 16:**
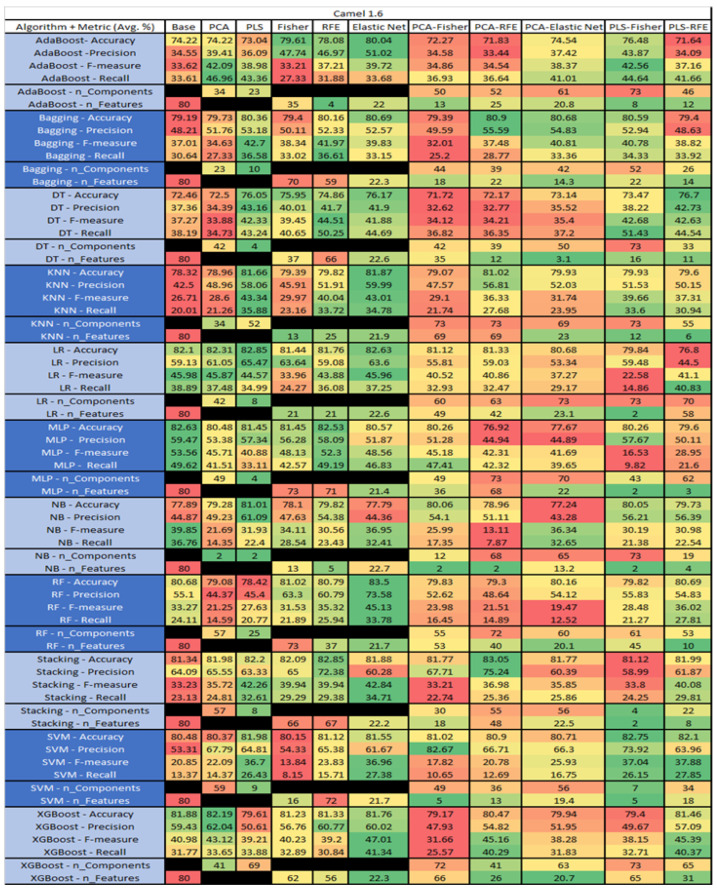
Camel 1.6 PROMISE Results Data Matrix.

**Figure 17:**
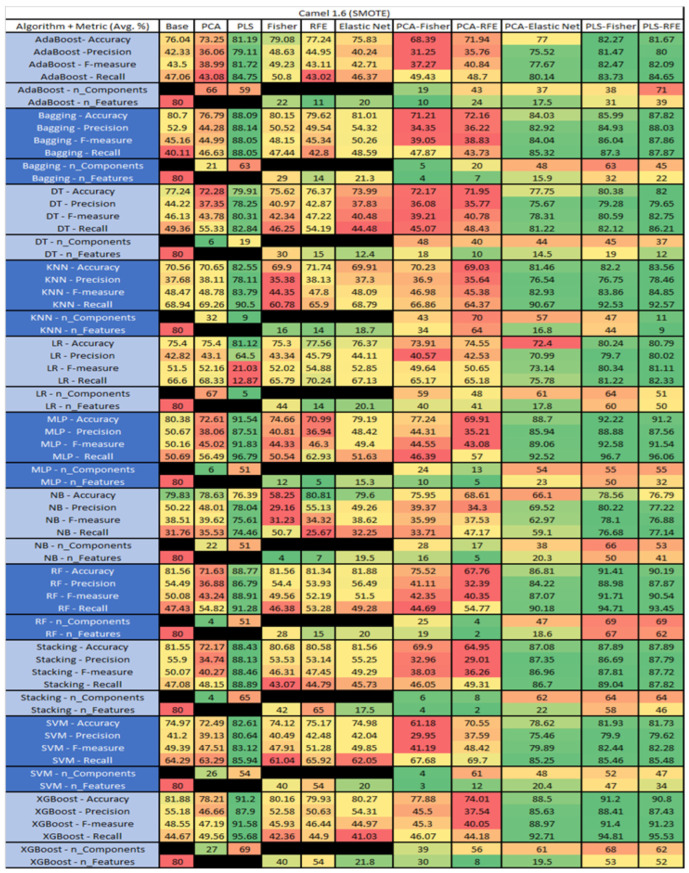
Camel 1.6 with SMOTE PROMISE Results Data Matrix.

**Figure 18:**
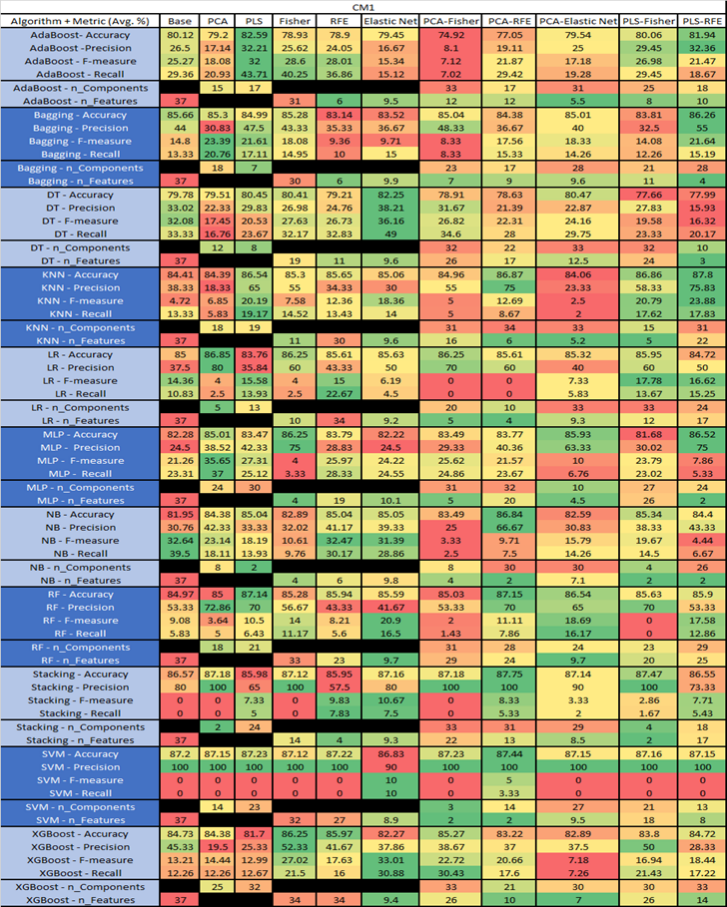
CM1 NASA MDP Results Data Matrix.

**Figure 19:**
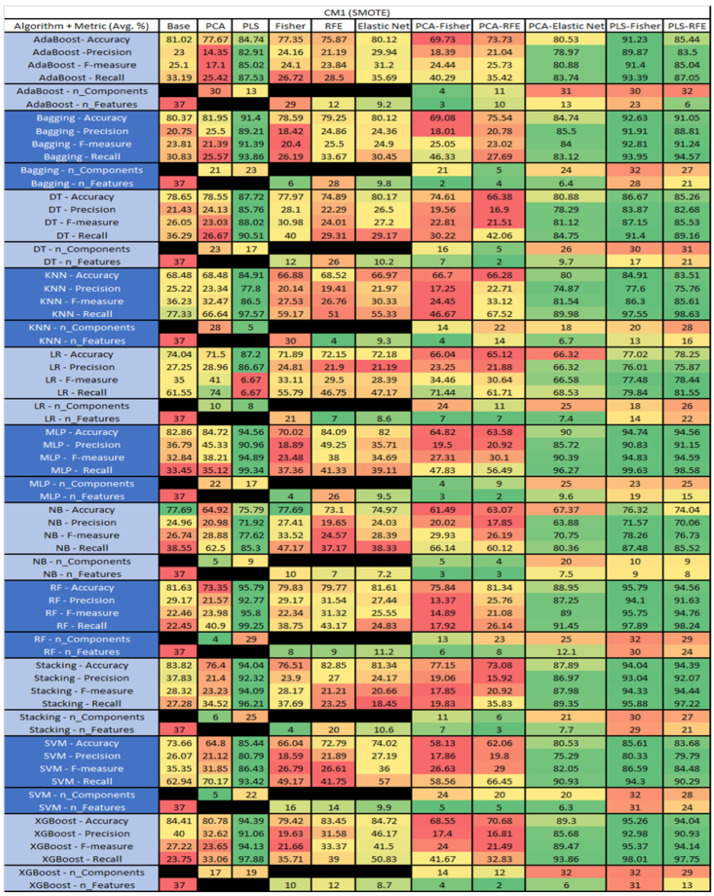
CM1 SMOTE NASA MDP Results Data Matrix.

**Figure 20:**
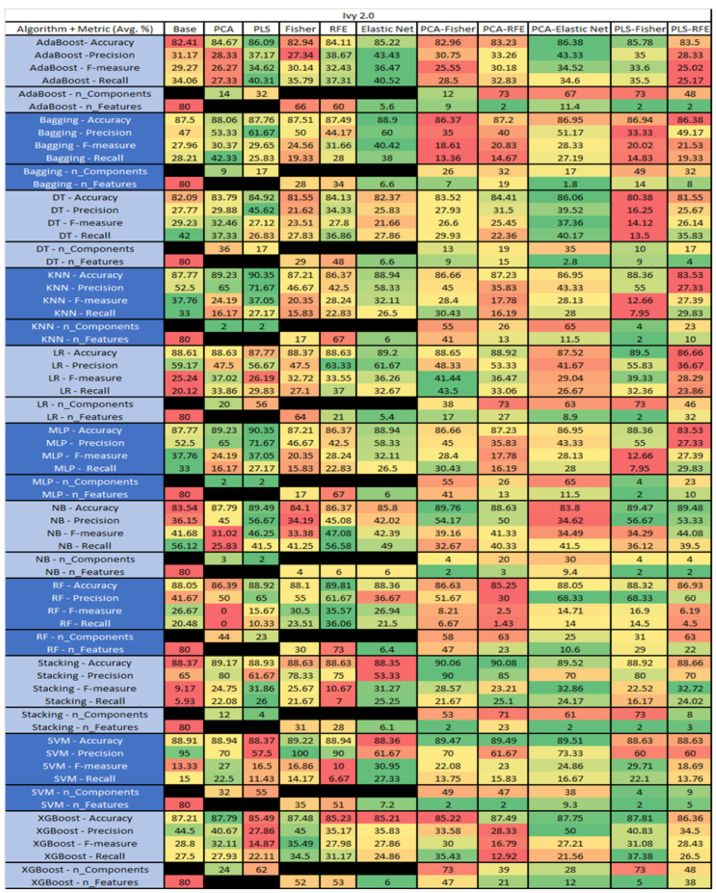
Ivy 2.0 PROMISE Results Data Matrix.

**Figure 21:**
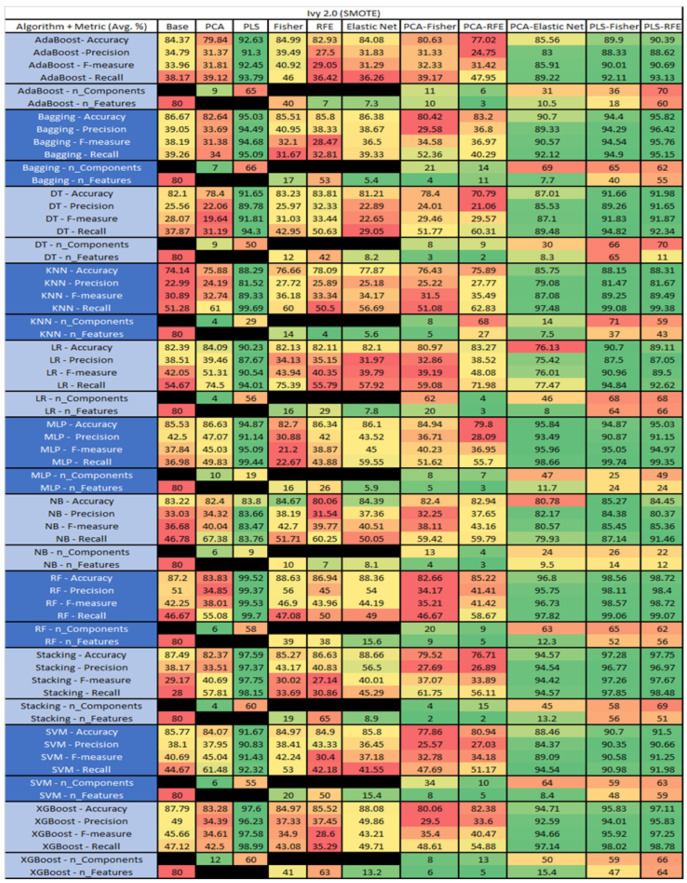
Ivy 2.0 SMOTE PROMISE Results Data Matrix.

**Figure 22:**
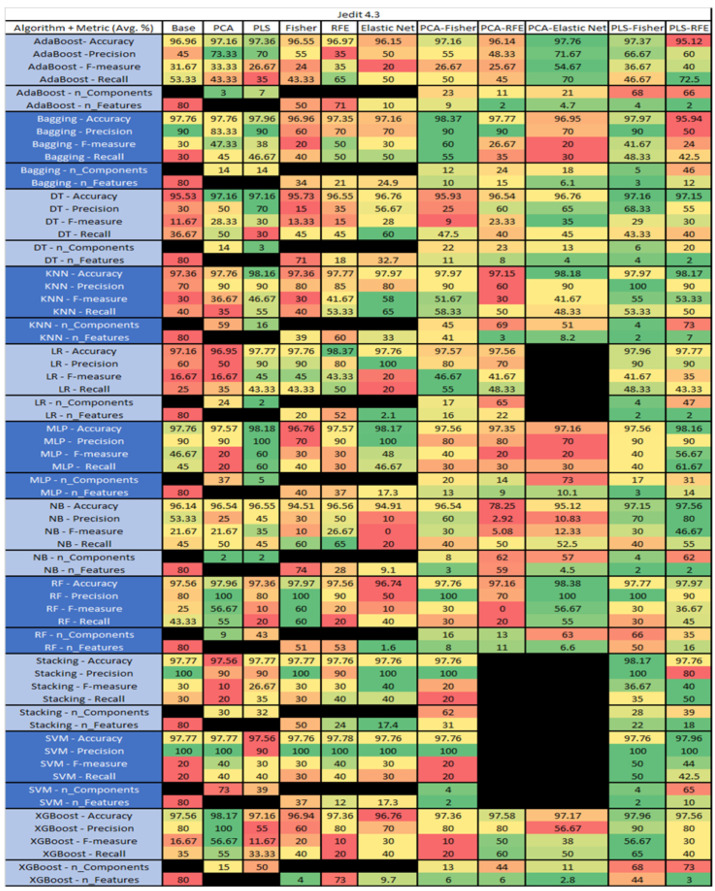
Jedit 4.3 PROMISE Results Data Matrix.

**Figure 23:**
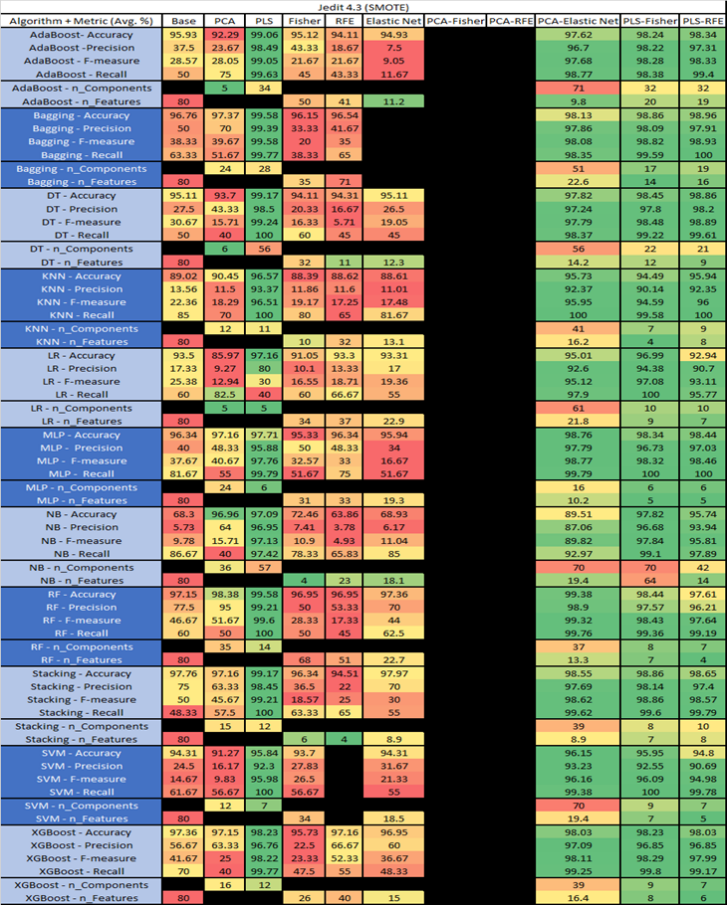
Jedit 4.3 SMOTE PROMISE Results Data Matrix.

**Figure 24:**
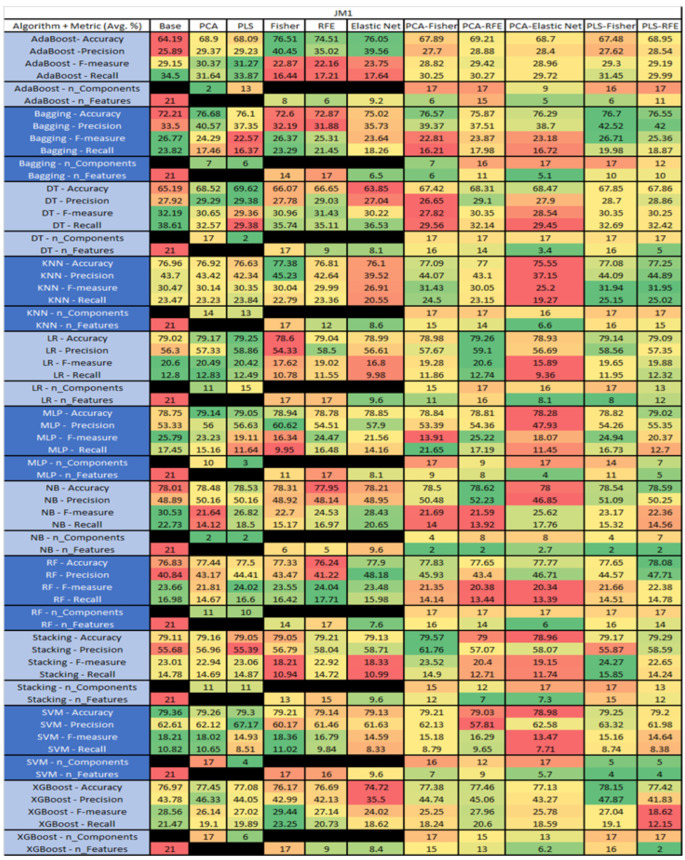
JM1 NASA MDP Results Data Matrix.

**Figure 25:**
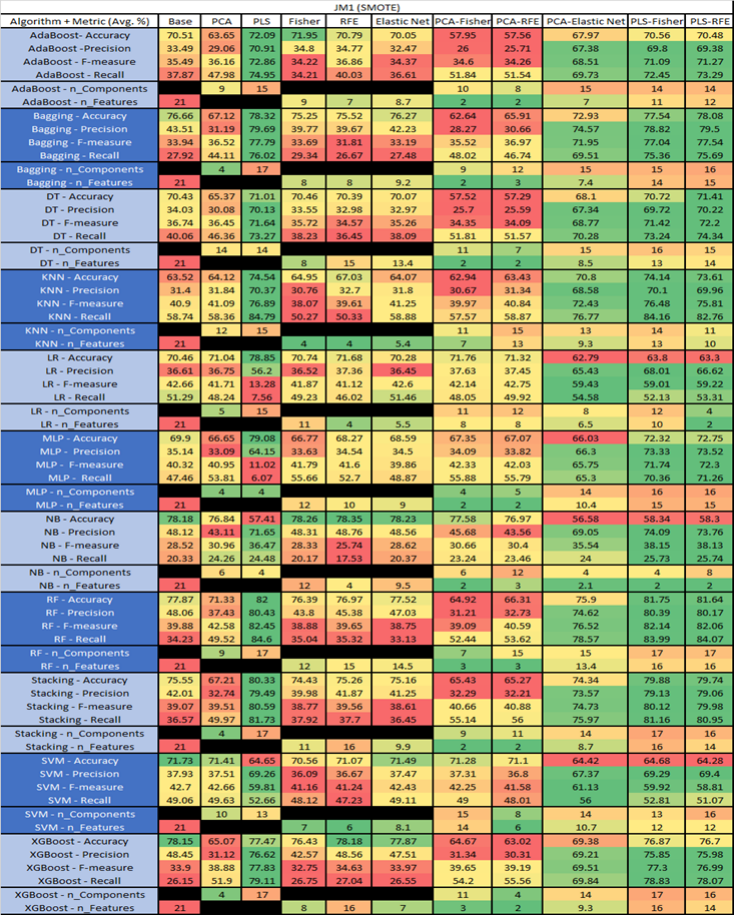
JM1 with SMOTE NASA MDP Results Data Matrix.

**Figure 26:**
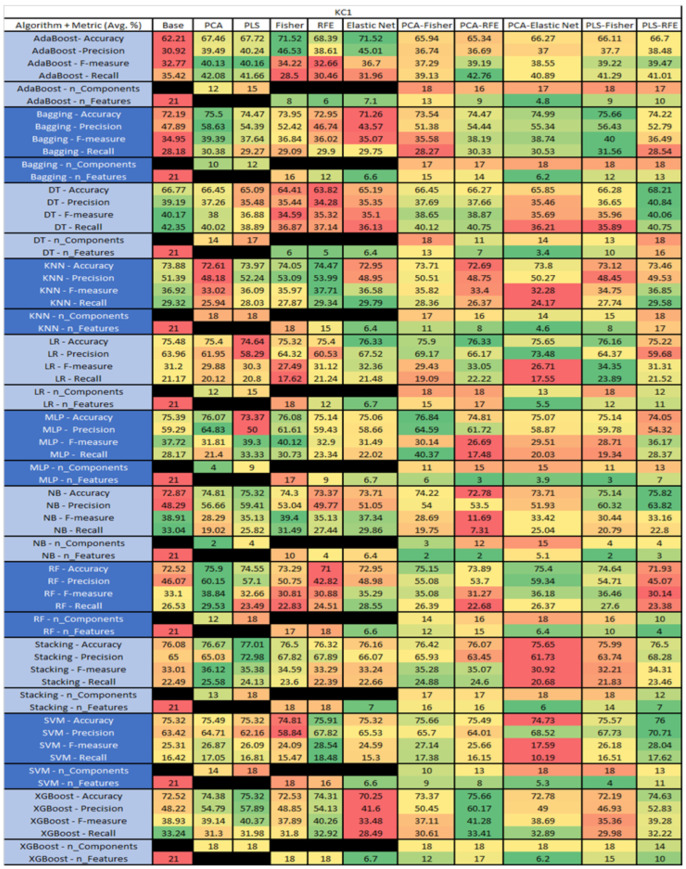
KC1 NASA MDP Results Data Matrix.

**Figure 27:**
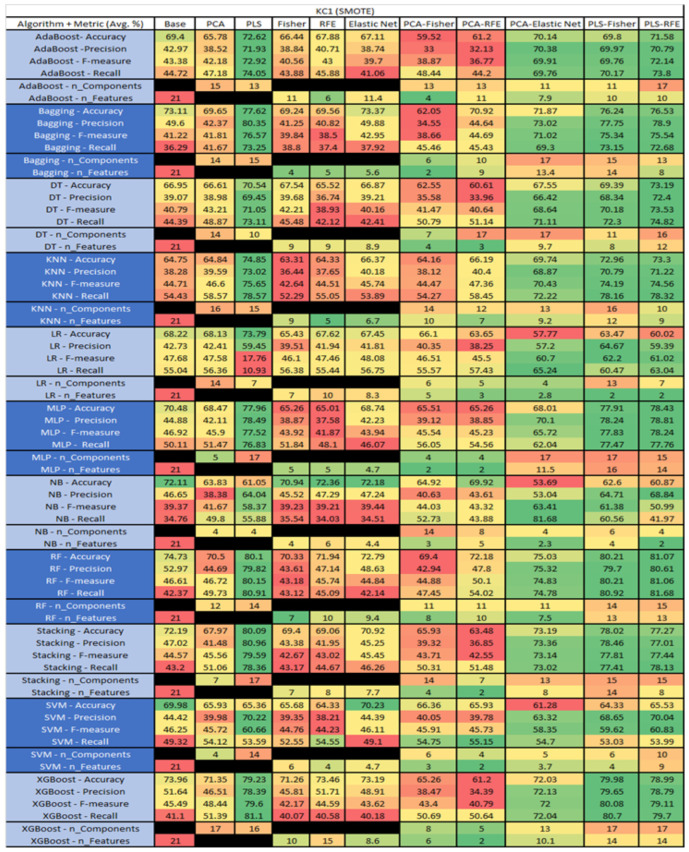
KC1 SMOTE NASA MDP Results Data Matrix.

**Figure 28:**
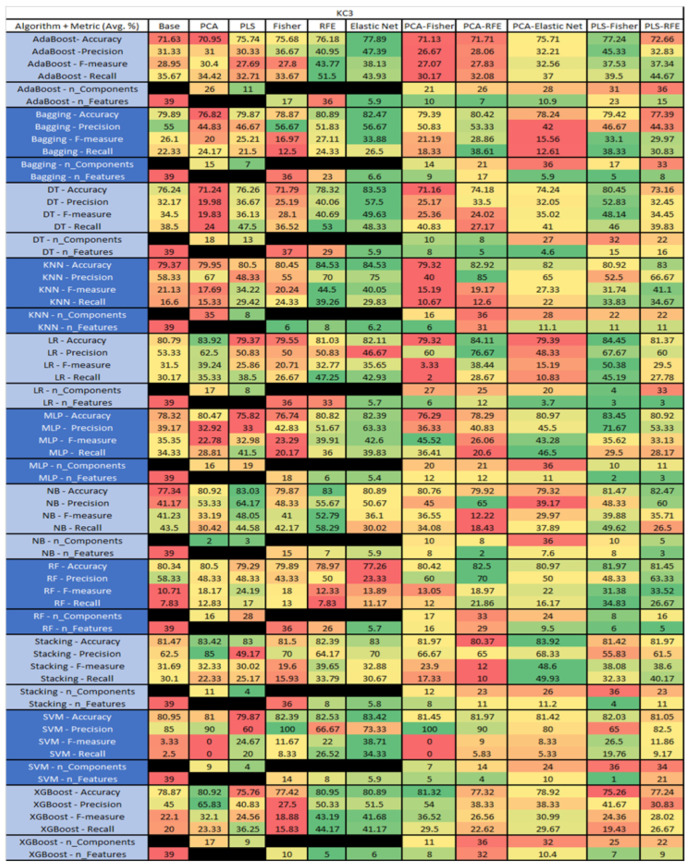
KC3 NASA MDP Results Data Matrix.

**Figure 29:**
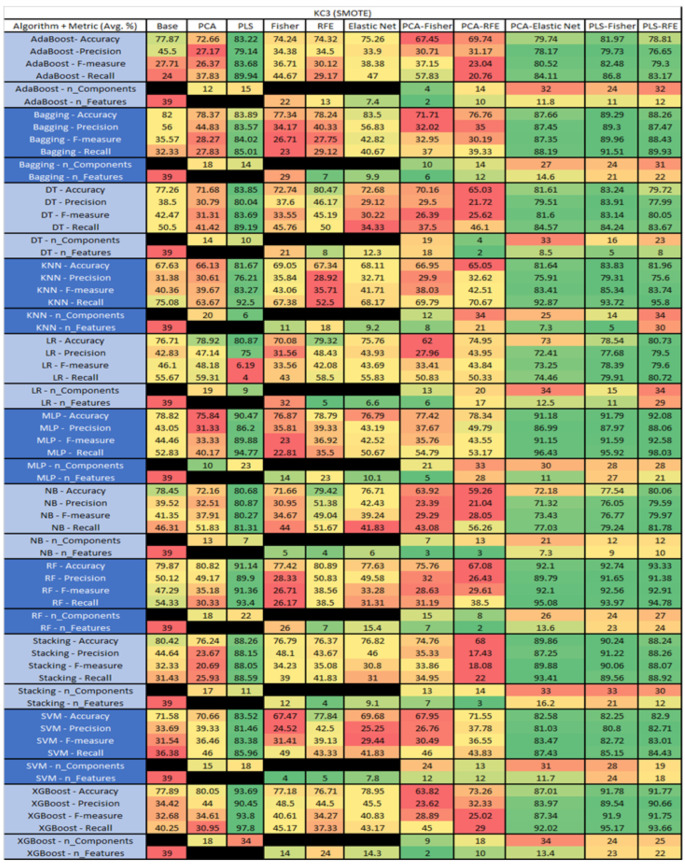
KC3 SMOTE NASA MDP Results Data Matrix.

**Figure 30:**
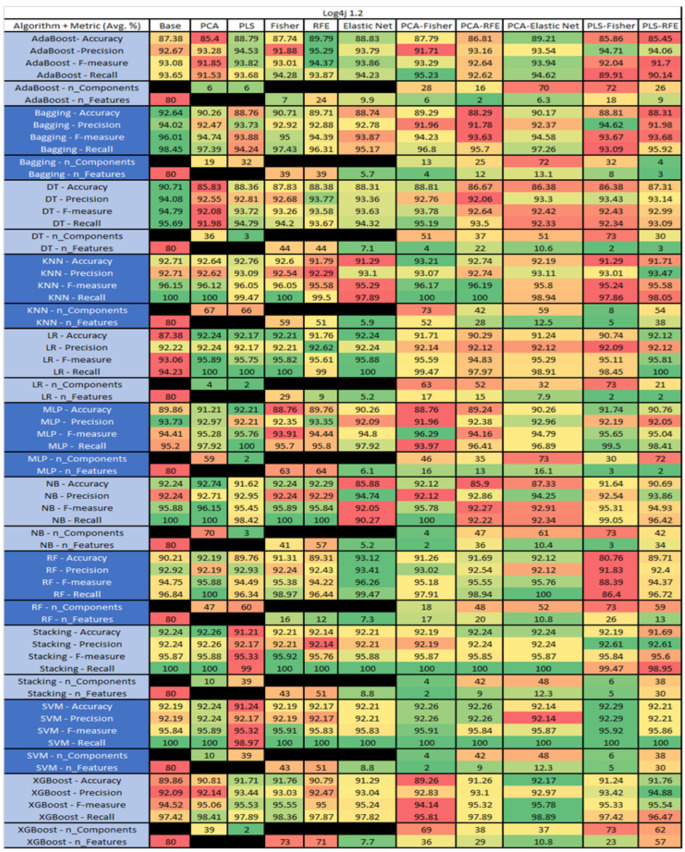
Log4j 1.2 PROMISE Results Data Matrix.

**Figure 31:**
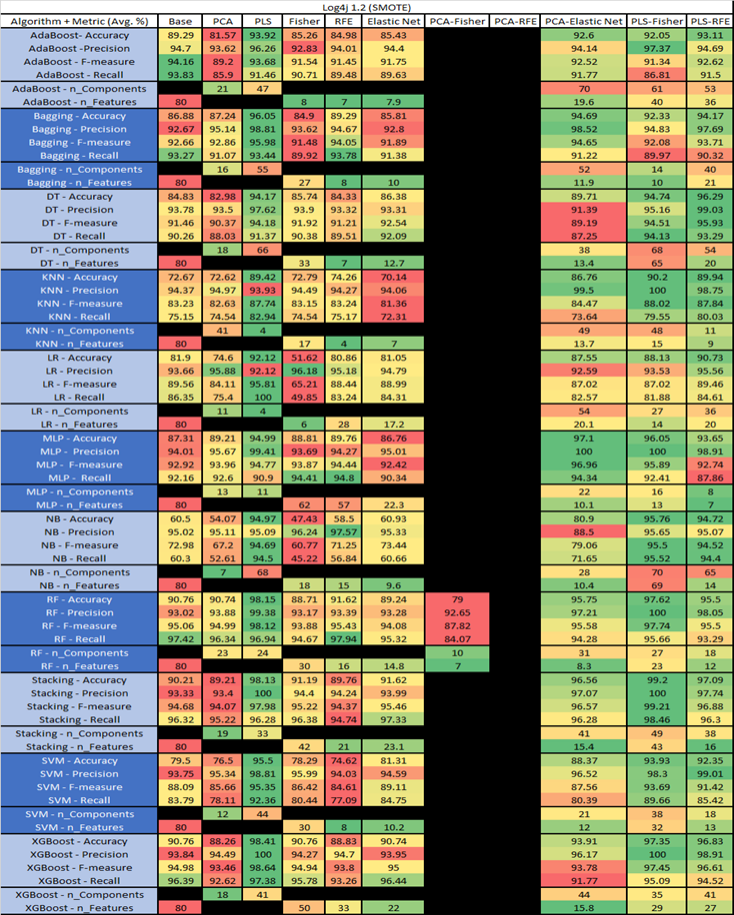
Log4j 1.2 SMOTE PROMISE Results Data Matrix.

**Figure 32:**
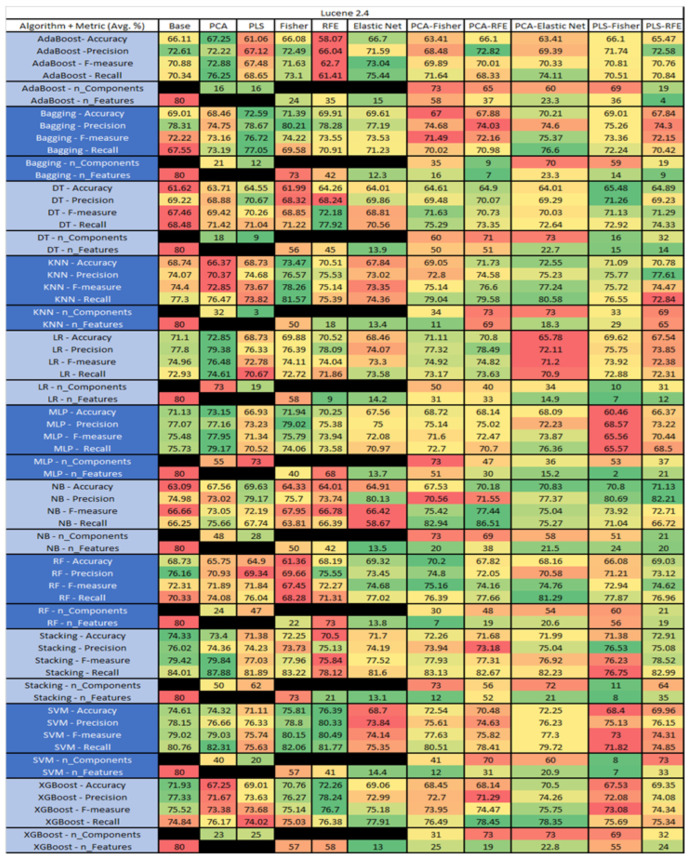
Lucene 2.4 PROMISE Results Data Matrix.

**Figure 33:**
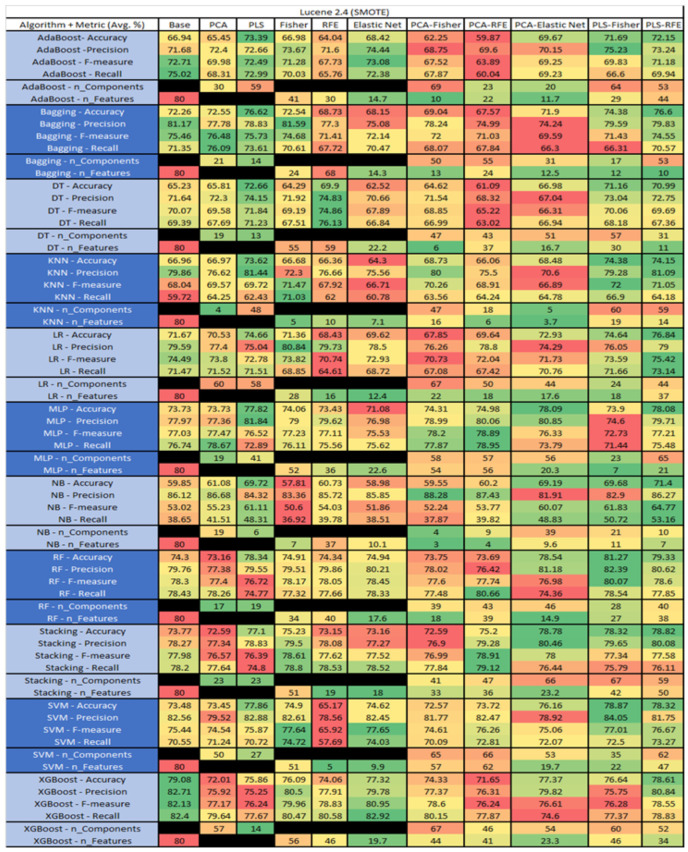
Lucene 2.4 SMOTE PROMISE Results Data Matrix.

**Figure 34:**
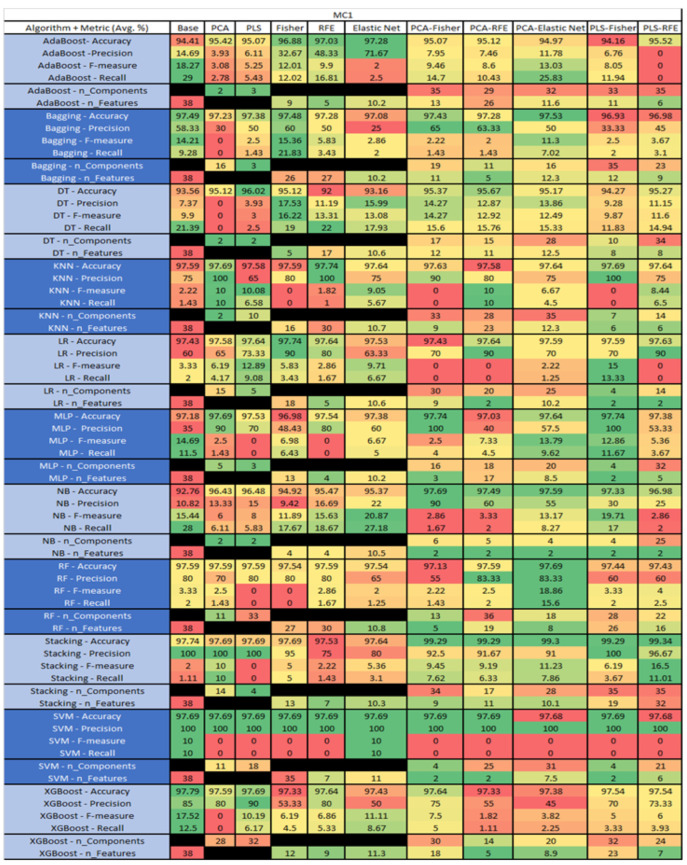
MC1 NASA MDP Results Data Matrix.

**Figure 35:**
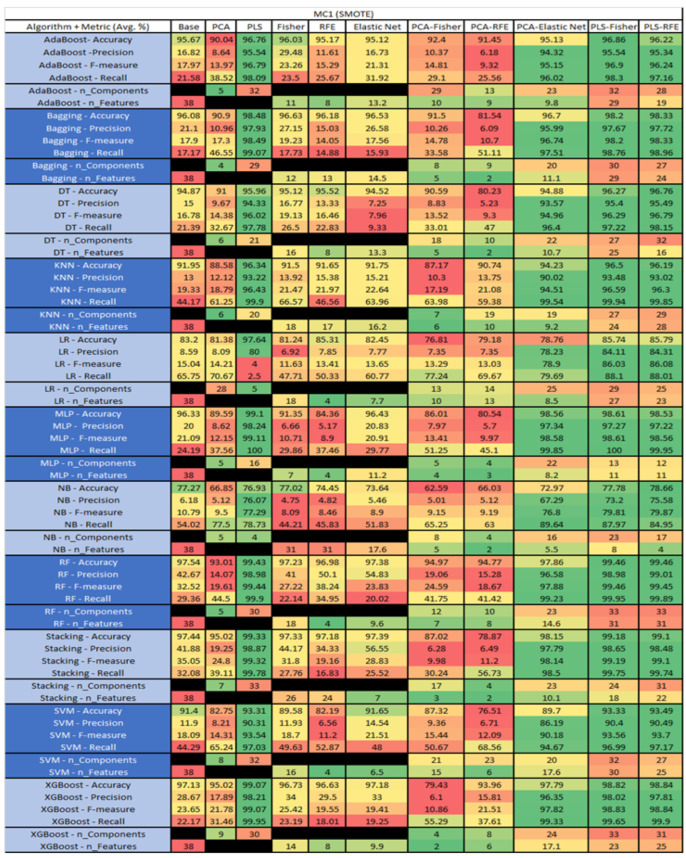
MC1 SMOTE NASA MDP Results Data Matrix.

**Figure 36:**
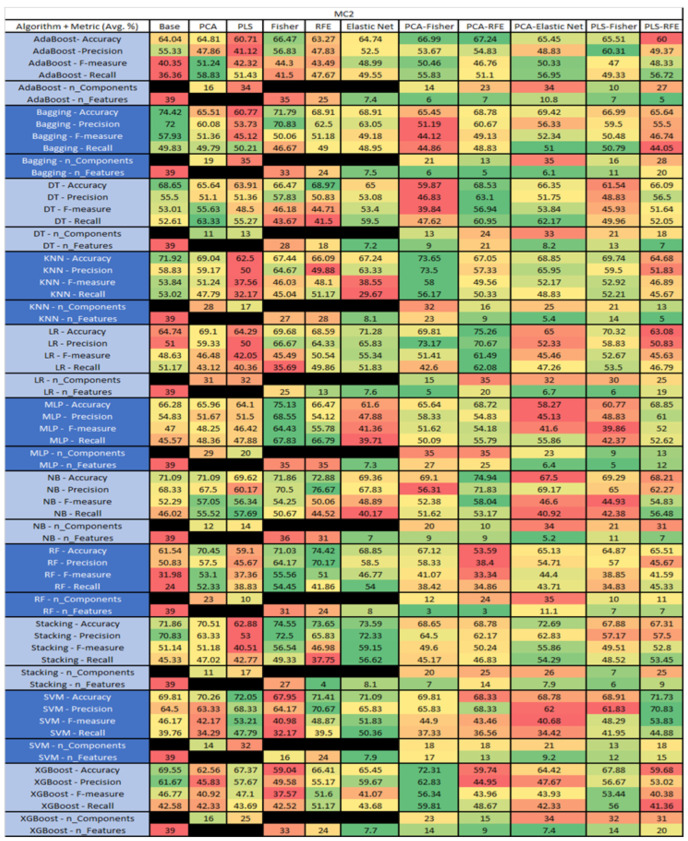
MC2 NASA MDP Results Data Matrix.

**Figure 37:**
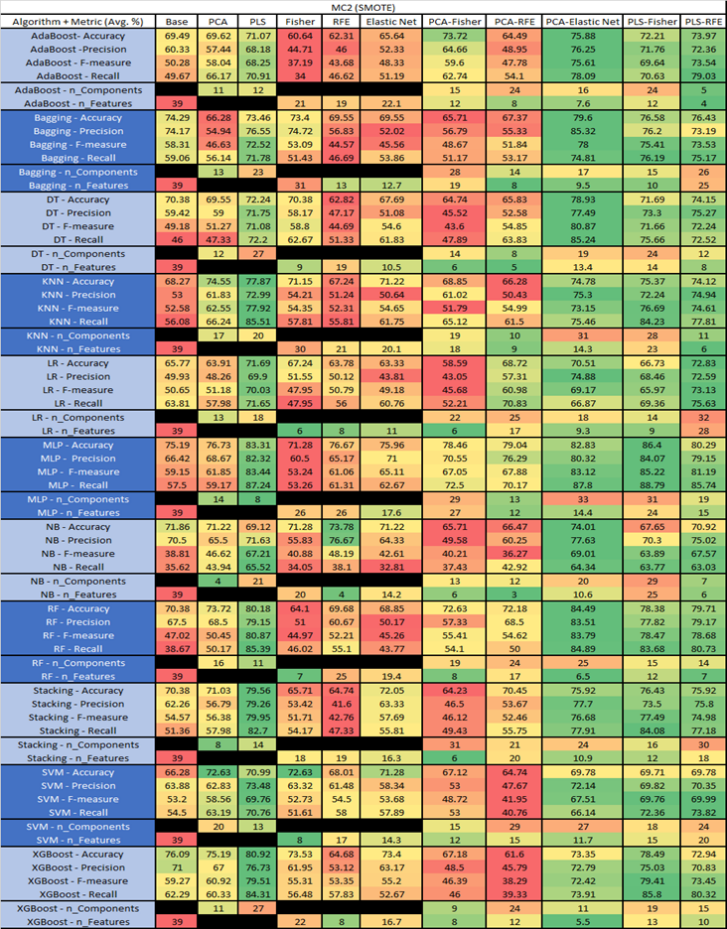
MC2 SMOTE NASA MDP Results Data Matrix.

**Figure 38:**
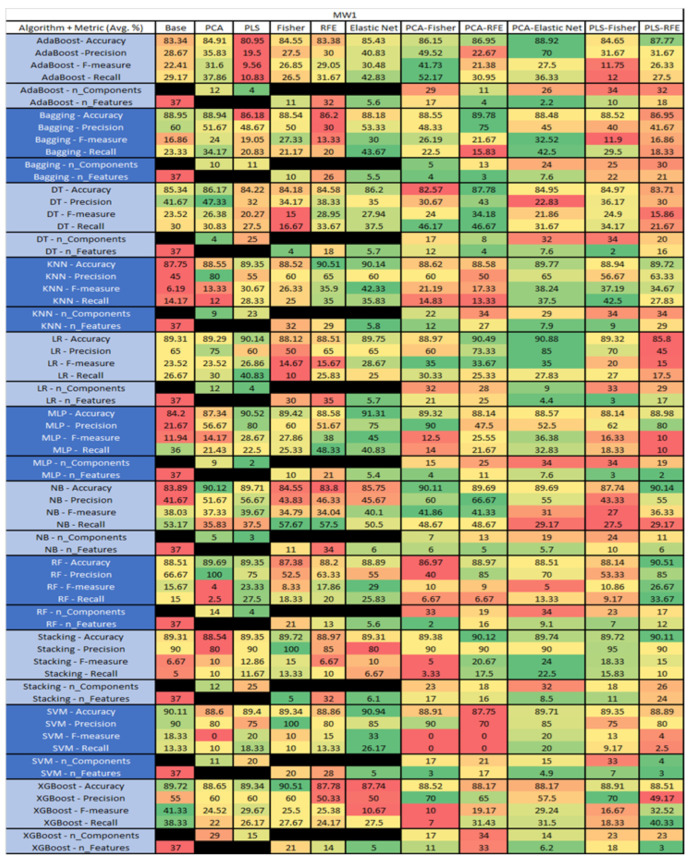
MW1 NASA MDP Results Data Matrix.

**Figure 39:**
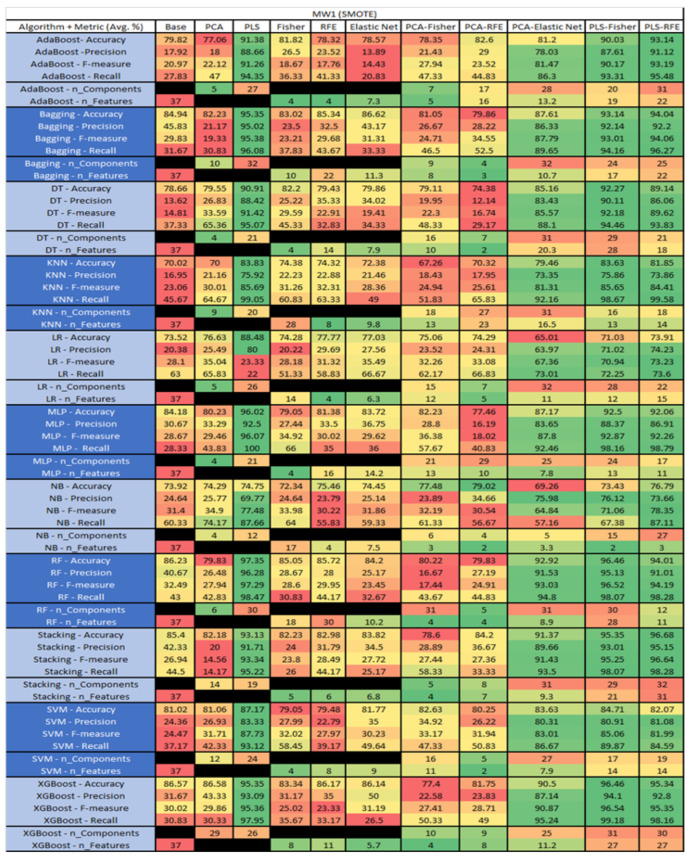
MW1 SMOTE NASA MDP Results Data Matrix.

**Figure 40:**
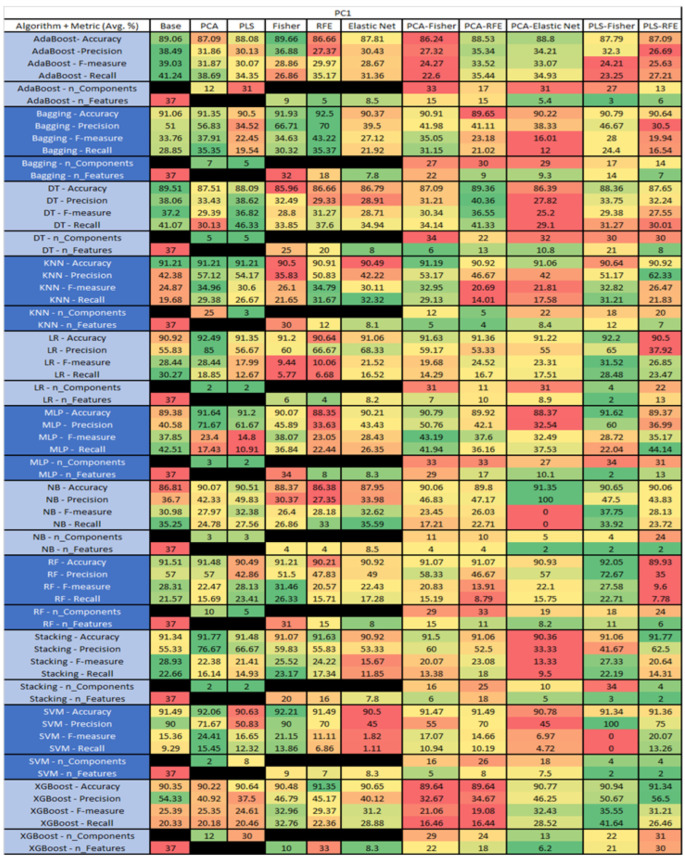
PC1 NASA MDP Results Data Matrix.

**Figure 41:**
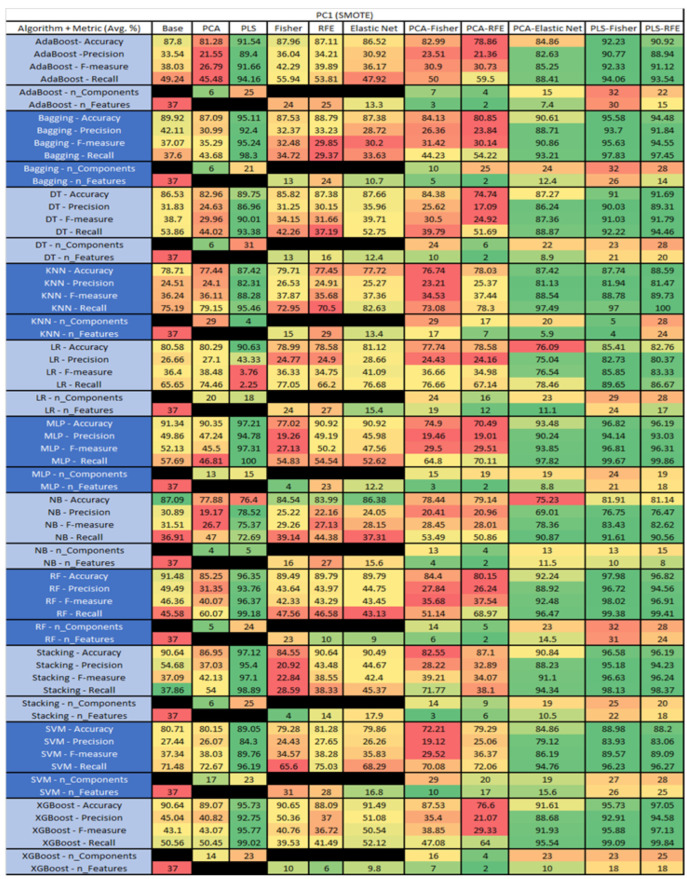
PC1 SMOTE NASA MDP Results Data Matrix.

**Figure 42:**
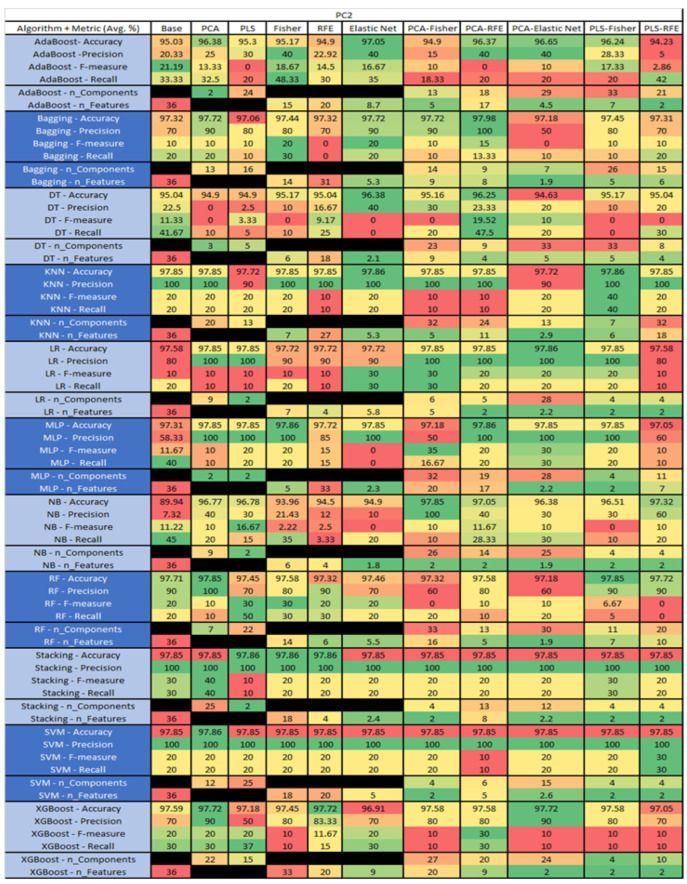
PC2 NASA MDP Results Data Matrix.

**Figure 43:**
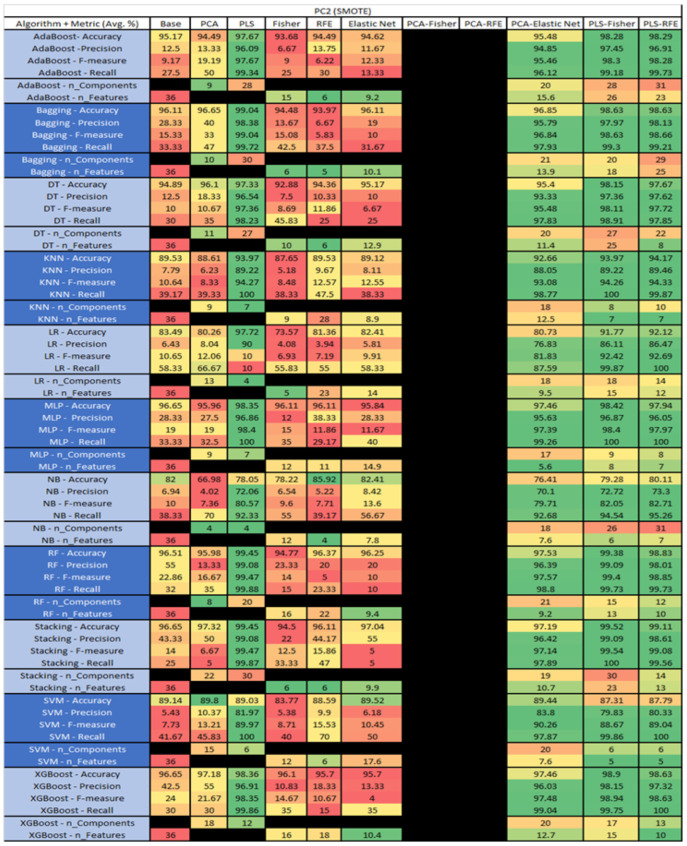
PC2 SMOTE NASA MDP Results Data Matrix.

**Figure 44:**
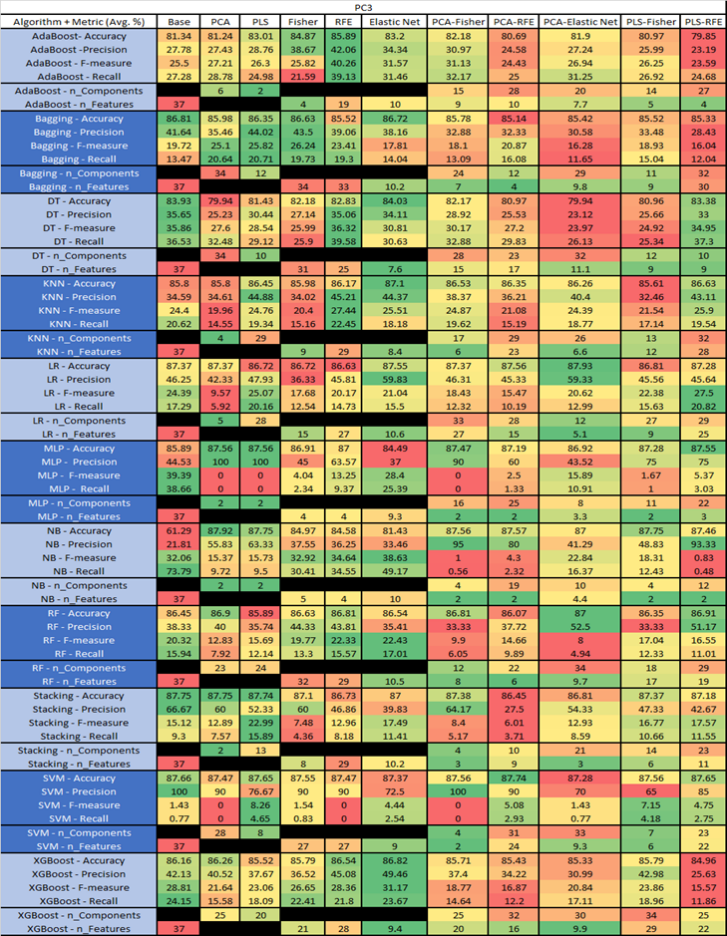
PC3 NASA MDP Results Data Matrix.

**Figure 45:**
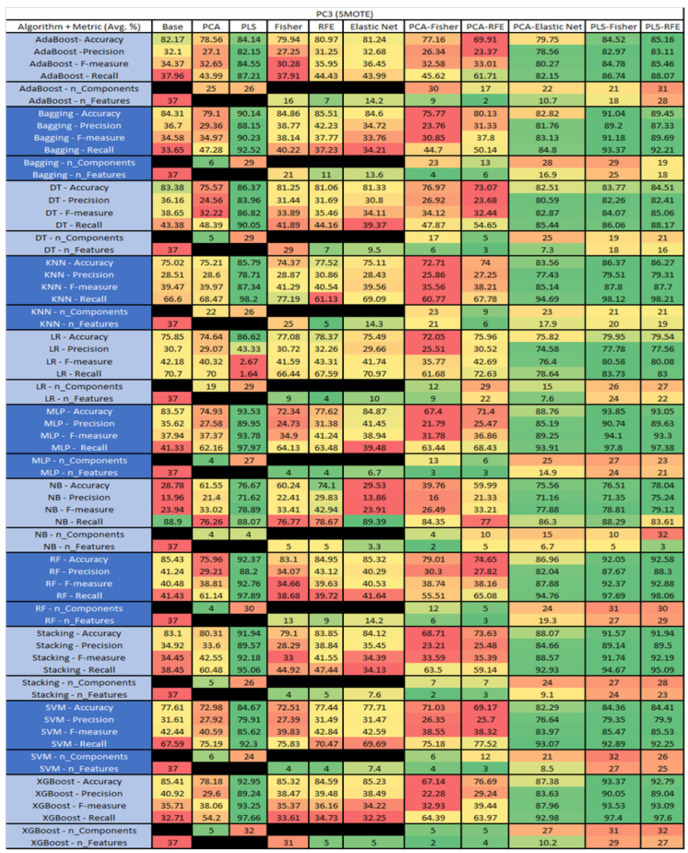
PC3 SMOTE NASA MDP Results Data Matrix.

**Figure 46:**
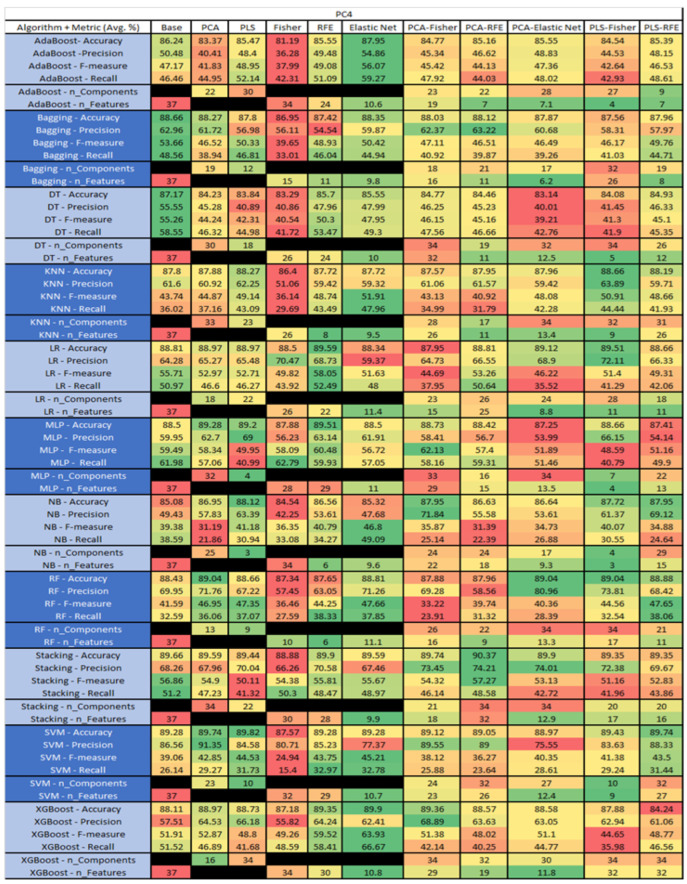
PC4 NASA MDP Results Data Matrix.

**Figure 47:**
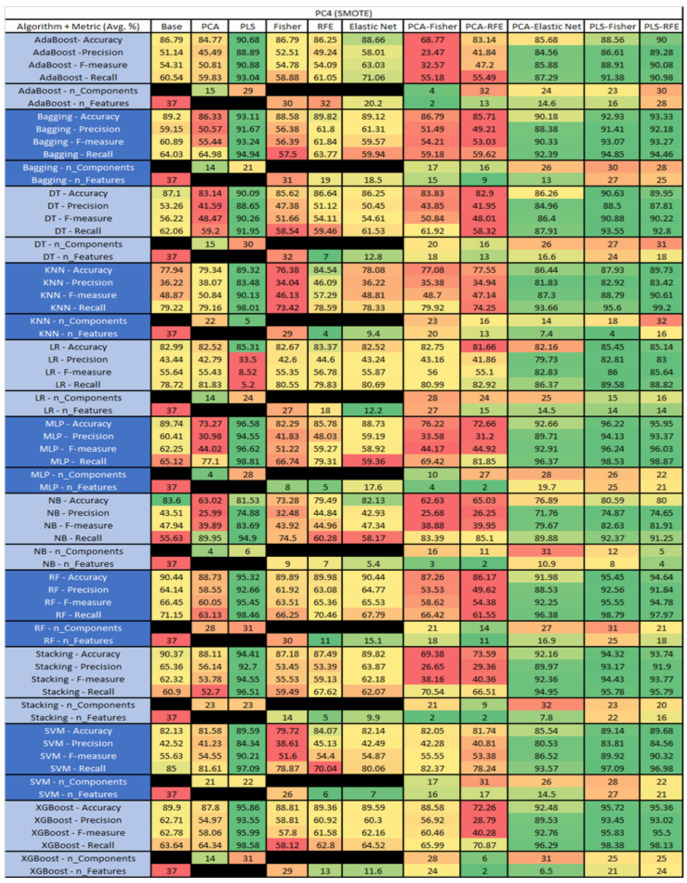
PC4 SMOTE NASA MDP Results Data Matrix.

**Figure 48:**
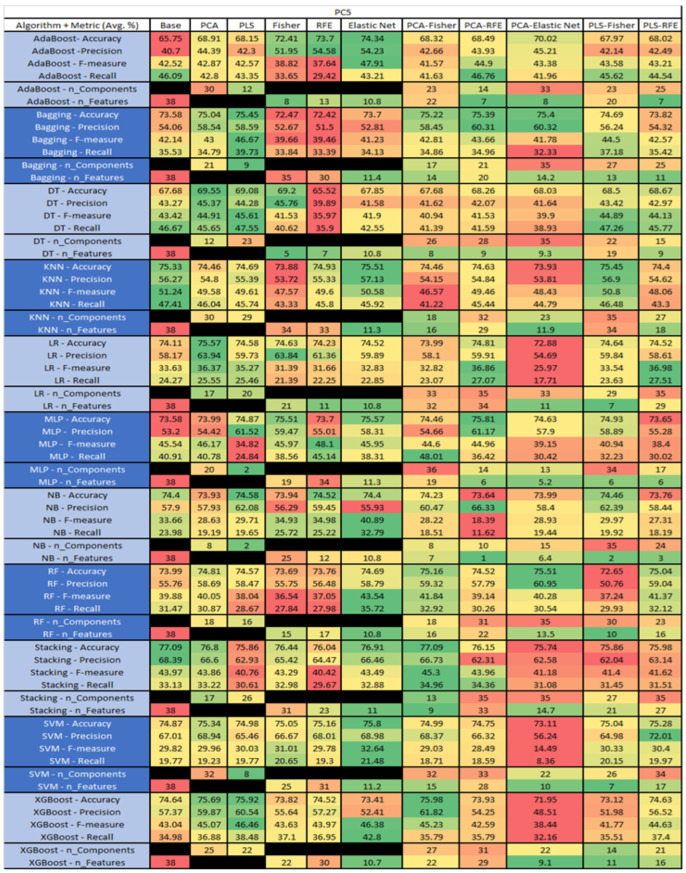
PC5 NASA MDP Results Data Matrix.

**Figure 49:**
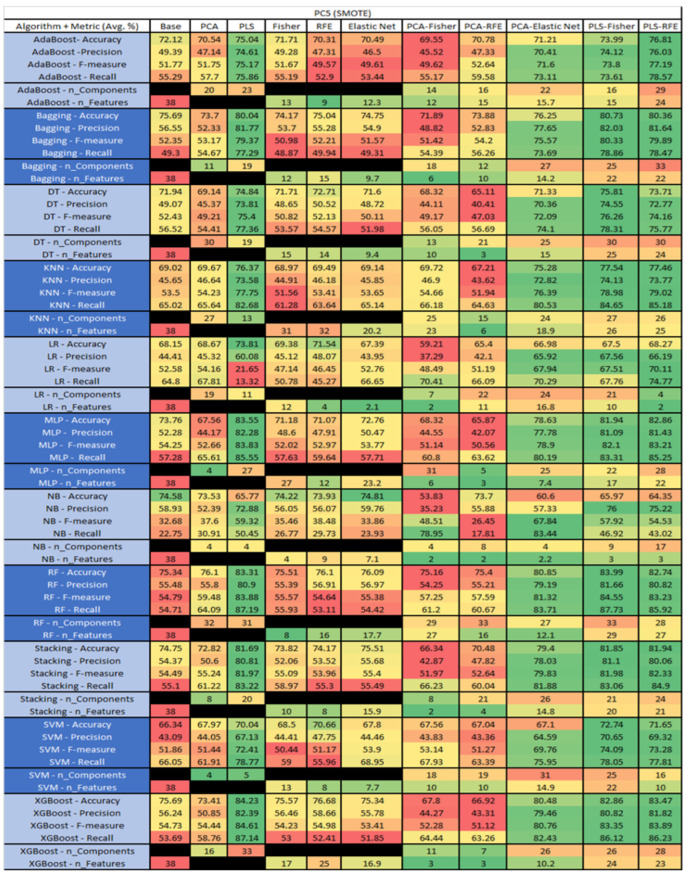
PC5 SMOTE NASA MDP Results Data Matrix.

**Figure 50:**
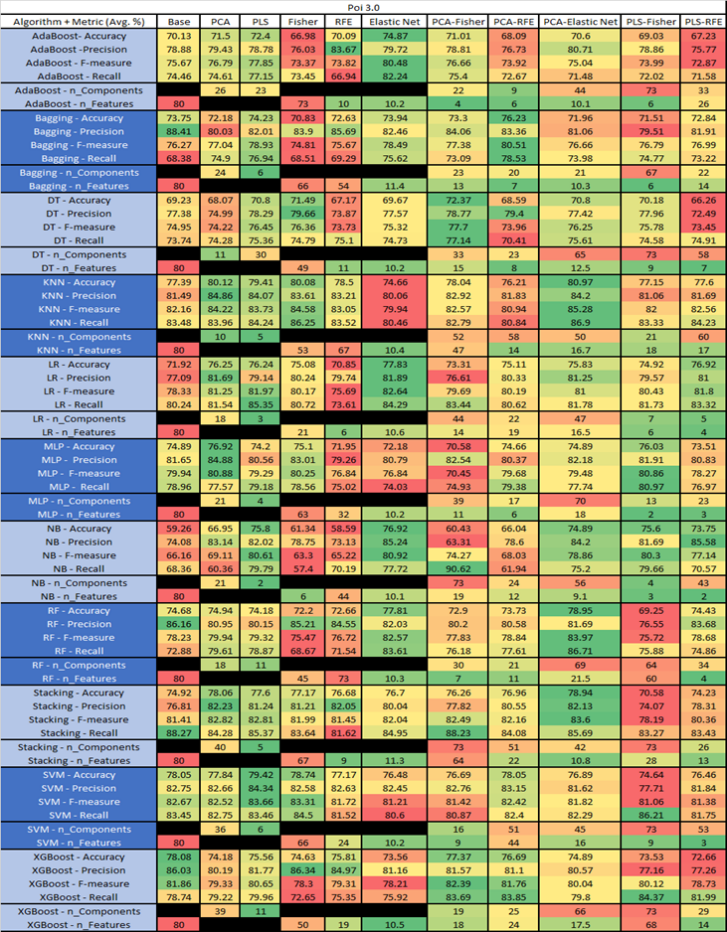
Poi 3.0 PROMISE Results Data Matrix.

**Figure 51:**
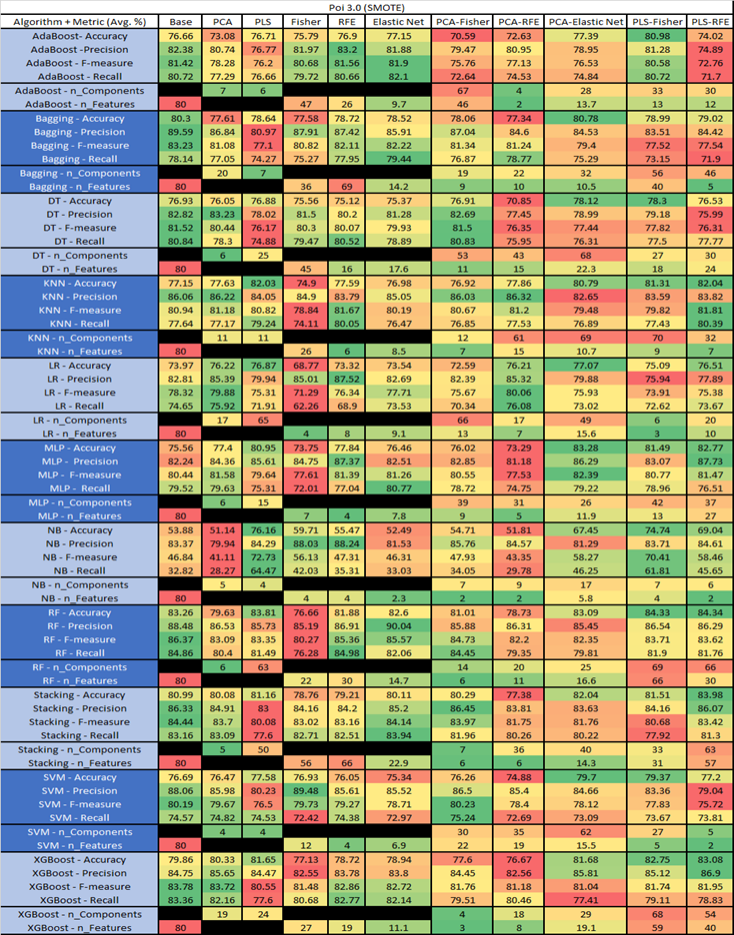
Poi 3.0 SMOTE PROMISE Results Data Matrix.

**Figure 52:**
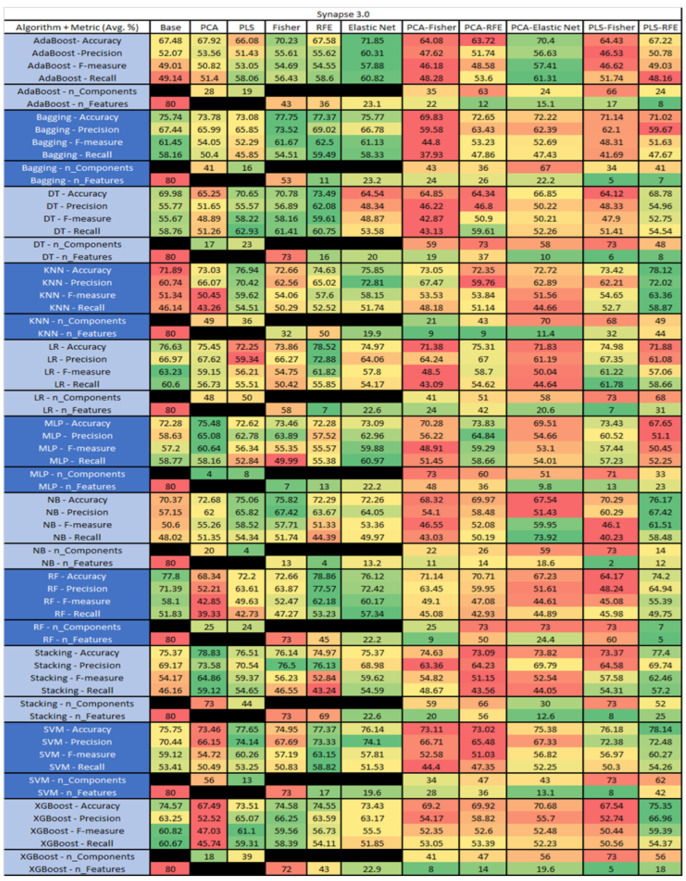
Synapse 3.0 PROMISE Results Data Matrix.

**Figure 53:**
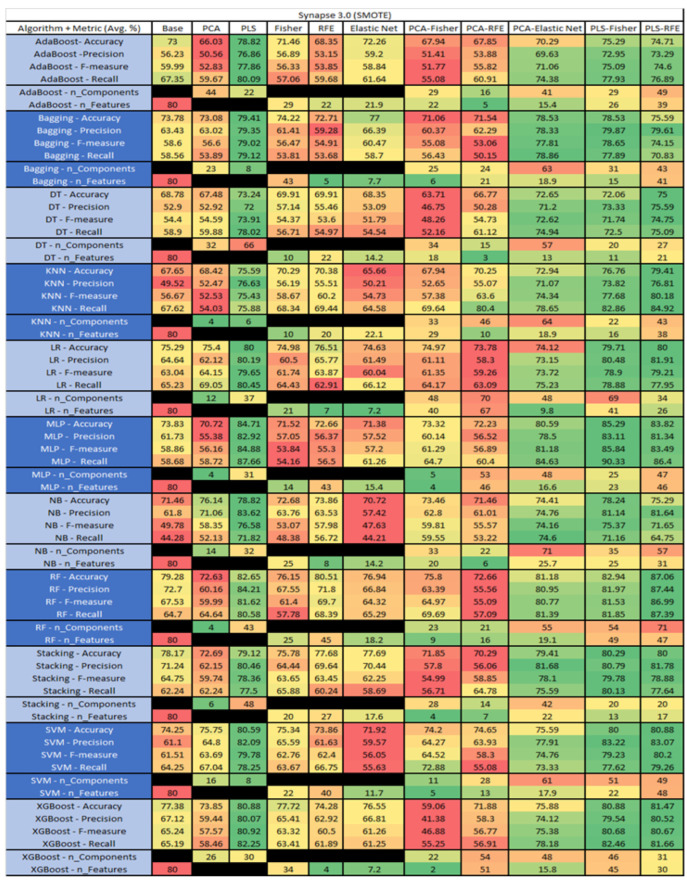
Synapse 3.0 SMOTE PROMISE Results Data Matrix.

**Figure 54:**
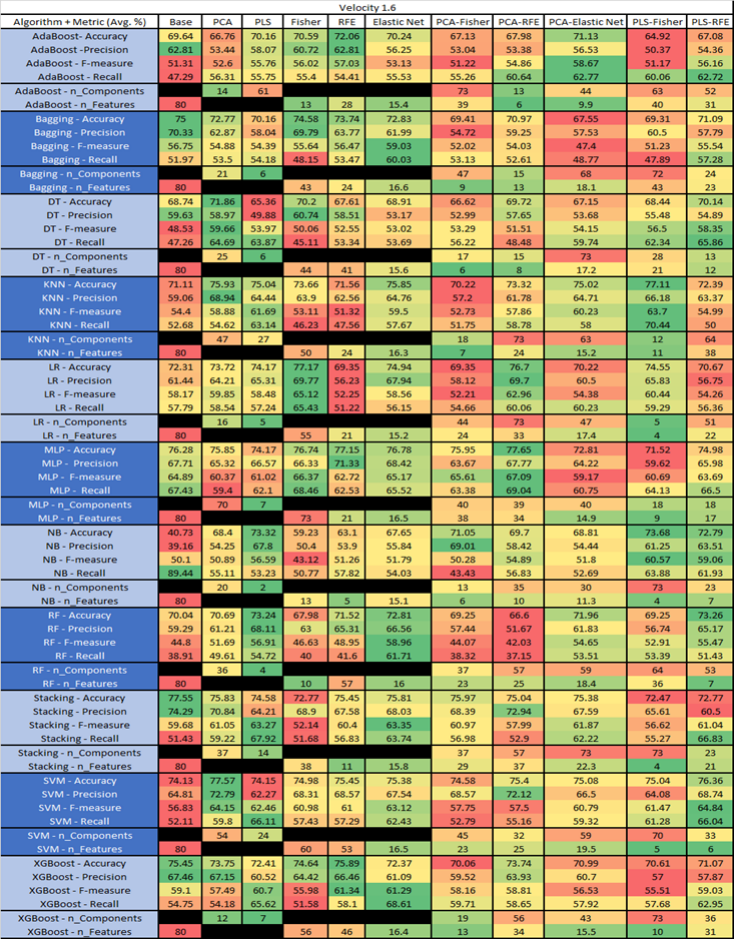
Velocity 1.6 PROMISE Results Data Matrix.

**Figure 55:**
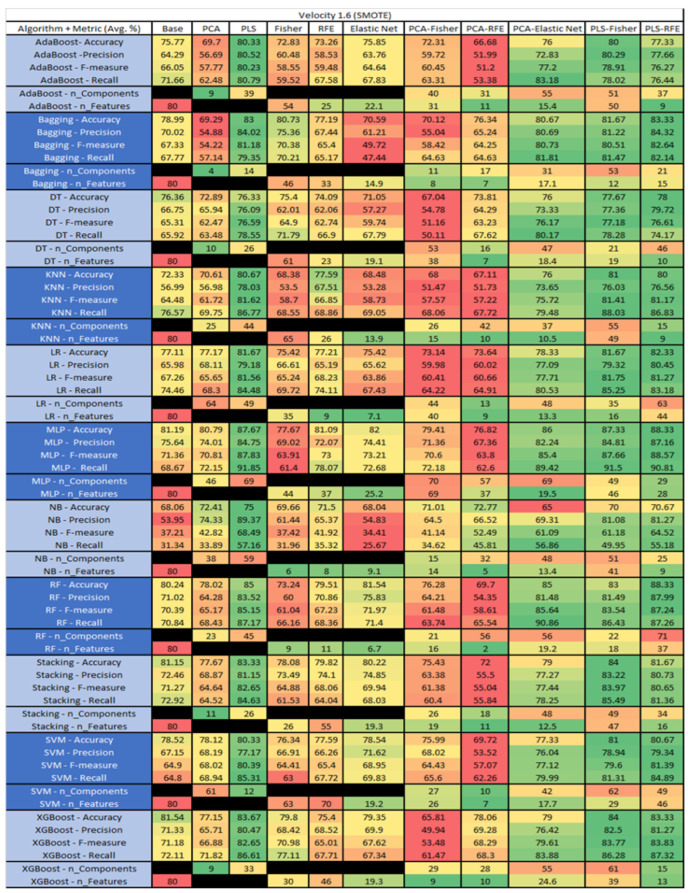
Velocity 1.6 SMOTE PROMISE Results Data Matrix.

**Figure 56:**
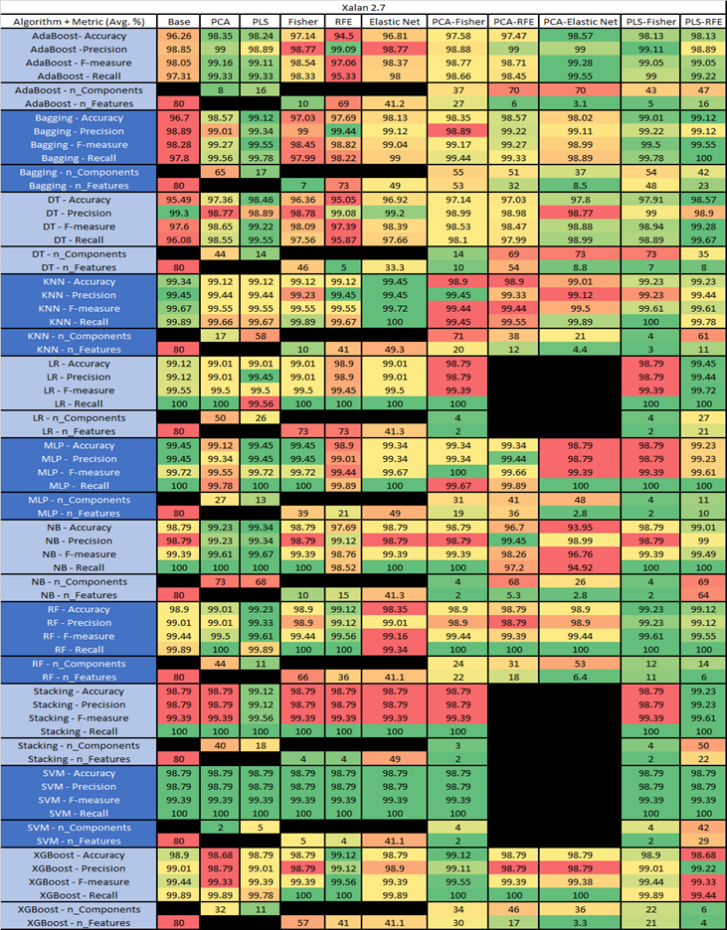
Xalan 2.7 PROMISE Results Data Matrix.

**Figure 57:**
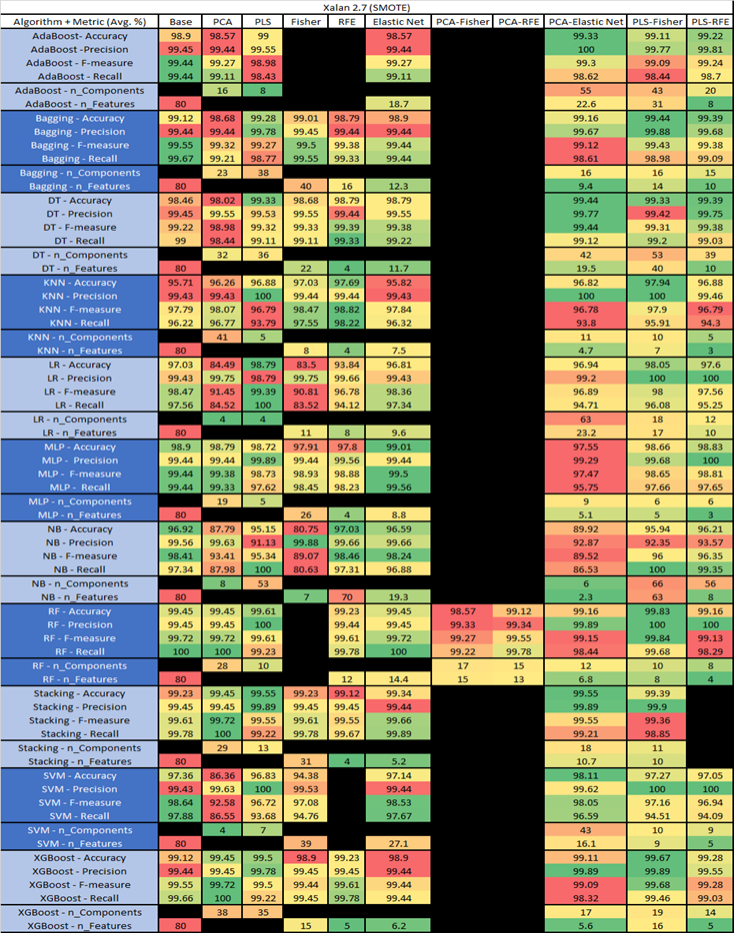
Xalan 2.7 SMOTE PROMISE Results Data Matrix.

**Figure 58:**
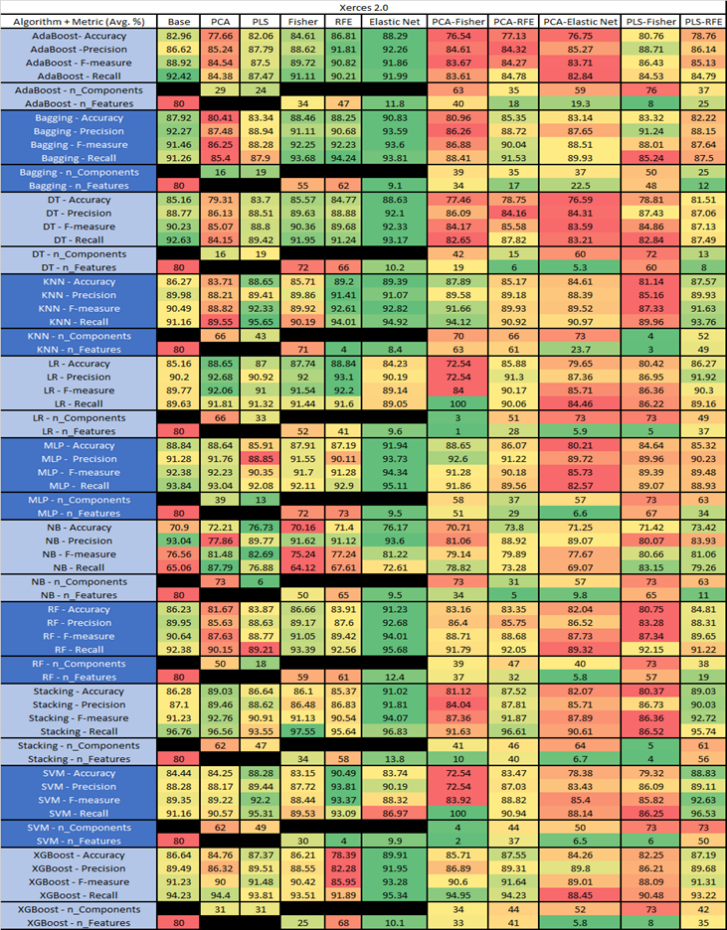
Xerces 2.0 PROMISE Results Data Matrix.

**Figure 59:**
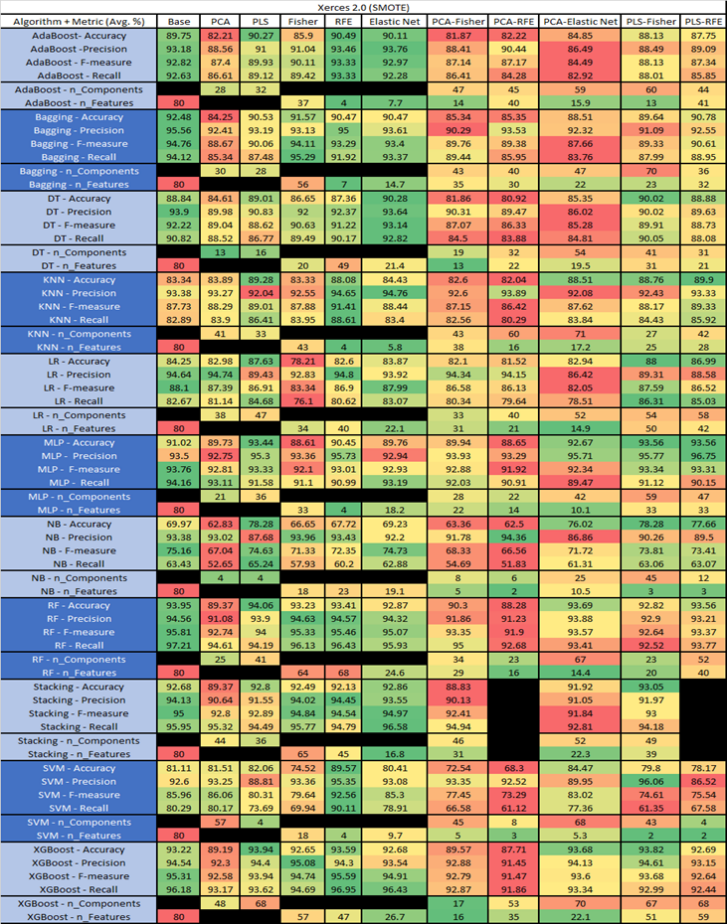
Xerces 2.0 SMOTE PROMISE Results Data Matrix.

The results are presented in Figure 14, Figure 15, Figure 16, Figure 17, Figure 18, Figure 19, Figure 20, Figure 21, Figure 22, Figure 23, Figure 24, Figure 25, Figure 26, Figure 27, Figure 28, Figure 29, Figure 30, Figure 31, Figure 32, Figure 33, Figure 34, Figure 35, Figure 36, Figure 37, Figure 38, Figure 39, Figure 40, Figure 41, Figure 42, Figure 43, Figure 44, Figure 45, Figure 46, Figure 47, Figure 48, Figure 49, Figure 50, Figure 51, Figure 52, Figure 53, Figure 54, Figure 55, Figure 56, Figure 57, Figure 58 and Figure 59, wherein each data matrix corresponds to one of the data sets tested, and each column of the data matrices represents a single technique. The first column represents the name of the algorithm as well as the metric being measured in an average of the quantification metrics, as follows: Accuracy, Precision, F1-Measure or F-measure, and Recall measured in (average %) for the first section, followed by the number of components, and the number of features. The remaining columnsare organized as follows: the BASE is used as the baseline for the model, PCA, Partial Least Square Regression (PLS), Fisher Score, RFE, Elastic Net, PCA–Fisher, PCA–RFE, PCA–ElasticNet, PLS–Fisher, and PLS–RFE. One of the challenges, when dealing with the number of metrics being evaluated, algorithms, and techniques, is presenting the results data in an easily readable and comparable way. The best way to represent and compare the data is a table, using the built-in excel function called conditional formatting with color scales. The scale was set to the lowest value in the case of the quantification metrics, the worst being a dark red color and the best or highest value being dark green in color. The number of features had the better value being lower, and the higher being worse. The original idea of this color scaling came from existing research used for coefficient/covariant matrix features. This showed that even large feature sets could display relevant information within a minimal area and, thus, we used it as our inspiration to present the results in tables with different colors.

The results include a brief description of the top performing algorithms, based on the all four performance metrics. The description of the results also includes the number of instances that were used in the construction of the models, as well as the number of base features as the total number of features in the data set. The results were collected over several weeks, with some models taking several hours to train on a data set. PLS-Elastic Net was, unfortunately. not tested, as the Elastic Net FS method was unable to use the components produced from the PLS technique.

The Ant 1.7, depicted in Figure 14, data set consists of 80 features, a sample size of 745 instances, of which 166 are true containing defects and 579 false without defects, and the true percentage accounting for 22.28%. The top three algorithms, MLP–PLS (average accuracy of 84.05%, average precision of 70.05%, average F-measure of 57.1% and an average recall of 49.3%), brought a reduction of the components down to just 2.

The performance of both Stacking–PCA–Elastic Net (average accuracy of 84.04%, average precision of 73.45%, average F-measure of 54.91%, and an average recall of 44.26% with a reduction in components to 61 and an average selection of 15.9), and LR–Elastic Net (average accuracy of 83.9%, average precision of 73.72%, average F-measure of 54.64% and an average recall of 45.32% with the average selected features being 12.5) were compared.

For the Ant 1.7 data set, Figure 15, where SMOTE was implemented, the top three algorithms were MLP–PLS–Fisher (average accuracy of 92.06%, average precision of 89.24%, average F-measure of 92.29% and an average recall of 95.69%, reduction of the components down to 63 with a selection of 51 among those). Comparative analysis of both MLP–PLS–RFE (average accuracy of 91.8%, average precision of 88.41%, average F-measure of 92.09%, and an average recall of 96.17%, with a reduction in components to 42 among those 38 were selected), and MLP–PLS (average accuracy of 91.71%, average precision of 88.53%, average F-measure of 92.01% and an average recall of 95.84%, seeing a reduction to 41 components) was conducted.

The results for Camel 1.6, depicted in Figure 16, show that the data set consisted of 80 features, a sample size of 927 instances, of which 188 were true (percentage accounting for 20.28%), containing defects, and 739 were false, without defect. The top algorithms were RF–Elastic Net (average accuracy of 83.5%, average precision of 73.58%, average F-measure of 45.13%, and an average recall of 34.78%, with reduction of features to an average of 21.7). Comparative analysis of Stacking–RFE was conducted (average accuracy of 82.85%, average precision of 72.38%, average F-measure of 39.94%, and an average recall of 39.38%, with reduction to 67 features). LR–PLS (average accuracy of 82.85%, average precision of 65.47%, average F-measure of 44.57%, and an average recall of 34.99%, with a reduction to 8 components), and MLP–Base (average accuracy of 82.63%, average precision of 59.47%, average F-measure of 53.56%, and an average recall of 49.62%) used all 80 features.

The results for the Camel 1.6 data set, seen in Figure 17, utilizing SMOTE, showed the top three algorithms as MLP–PLS–Fisher (average accuracy of 92.22%, average precision of 88.88%, average F-measure of 92.58%, and an average recall of 96.7%, with reduction to 55 components). MLP–PLS performed comparably (average accuracy of 91.54%, average precision of 87.51%, average F-measure of 91.83%, and an average recall of 96.79%, showing a reduction to 51 components). RF–PLS–Fisher (average accuracy of 91.41%, average precision of 88.98%, average F-measure of 91.71%, and an average recall of 94.71%, with reduction to 51 components ). In addition, in our analysis we observed that the algorithms with the best performances were MLP–PLS–RFE, XGBoost–PLS, and XGBoost–PLS–Fisher.

The results for the CM1 data set, in Figure 18, consisted of 37 features, a sample size of 327 instances, of which 42 were true, containing defects, and 285 false, without defect, the true percentage accounting for 12.84%. The top three algorithms were KNN–LS–RFE (average accuracy of 87.8%, average precision of 75.83%, average F-measure of 23.88%, and an average recall of 17.83%, with a reduction to 31 components, of which 22 were selected). The other two were KNN–PLS–Fisher (average accuracy of 86.86%, average precision of 58.33%, average F-measure of 20.79%, and an average recall of 17.62%, with reduction to 15 components of which 5 were selected), and KNN–PLS (average accuracy of 86.54%, average precision of 65%, average F-measure of 20.19%, and an average recall of 19.17%, with reduction to 19 components). Within this data set, a class imbalance and its effects on each of the models was witnessed, in particular, with the SVM, Stacking, LR, and RF models, with overall reductions in the F-measure and Recall metrics.

The results for the CM1 data set that utilized SMOTE can be found in Figure 19. The top three algorithms were RF–PLS (average accuracy of 95.79%, an average precision of 92.77%, an average F-measure of 95.8%, and a recall of 99.25%, with a reduction to 29 components). Followed by RF–PLS–Fisher (average accuracy of 95.79%, an average precision of 94.1%, an average F-measure of 95.75%, and a recall of 97.89%, with a reduction to 32 components of those 30 were selected) and XGBoost–PLS–Fisher (average accuracy of 95.26%, an average precision of 92.98%, an average F-measure of 95.37%, and a recall of 98.01%, with a reduction to 32 components, of which 31 were selected).

The results for Ivy 2.0, depicted in Figure 20, showed that this data set consisted of 80 features, a sample size of 352 instances, of which 40 were true, containing defects, and 312 false, without defect, and the true percentage accounting for 11.36%. The top four algorithms were KNN–PLS and MLP–PLS, both with (average accuracy of 90.35%, average precision of 71.76%, average F-measure of 37.05%, and an average recall of 27.17%, with a reduction to 2 components ). The remaining two were Stacking–PCA–RFE (average accuracy of 90.08%, average precision of 85%, average F-measure of 23.21%, and an average recall of 25.1%, with a component reduction to 71 of which 23 were selected), and Stacking PCA–Fisher(average accuracy of 90.06%, average precision of 90%, average F-measure of 28.57%, an average recall of 21.67%, with a reduction to 53 components, of which 2 were selected). Notably, the other models with acceptable performance were SVM, NB and RF, with some class imbalance issues witnessed in a few of the F-measure and Recall metrics.

The results for the Ivy 2.0 data set that utilized SMOTE are found in Figure 21, The top three algorithms were RF–PLS (average accuracy of 99.52%, an average precision of 99.37%, an average F-measure of 99.53%, and a recall of 99.7%, with a reduction to 58 components), followed by RF–PLS–RFE (average accuracy of 98.72%, an average precision of 98.4%, an average F-measure of 98.72%, and a recall of 99.07%, with a reduction to 62 components of those 56 were selected) and RF–PLS–Fisher (average accuracy of 98.56%, an average precision of 98.11%, an average F-measure of 98.57%, and a recall of 99.06%, with a reduction to 65 components, of which 56 were selected).

The results for Jedit 4.3 data set are found in Figure 22. It consisted of 80 features, a sample size of 492 instances, of which 11 were true, containing defects, and 481 false, without defect, and the true percentage accounting for 2.23%. The top three algorithms were RF–PCA–Elastic Net (average accuracy of 98.38%, average precision of 100%, average F-measure of 56.67%, and an average recall of 55%, with a reduction to 63 components of which an average of 6.6 were selected). The remaining two were Bagging–PCA–Fisher (average accuracy of 98.37%, average precision of 90%, average F-measure of 60%, and an average recall of 55%, with a reduction to 12 components, of which 10 were selected), and MLP–PLS (average accuracy of 98.18%, average precision of 100%, average F-measure of 60%, and an average recall of 60%, with a reduction to 5 components). LR, SVM, and Stacking PCA-Elastic Net were unable to converge, possibly due to the class imbalance of the output class of the bugs in the data set, which, in turn, affected Stacking and SVM–PCA–RFE.

The results for the Jedit 4.3 data set that utilized SMOTE are in Figure 23. The top three algorithms were RF–PLS (average accuracy of 99.58%, an average precision of 99.21%, an average F-measure of 99.53%, and a recall of 100%, with a reduction to 14 components), followed by Bagging–PLS (average accuracy of 99.58%, an average precision of 99.38%, an average F-measure of 99.58%, and a recall of 99.77%, with a reduction to 28 components) and RF–PCA–Elastic Net (average accuracy of 99.38%, an average precision of 98.9%, an average F-measure of 99.32%, and a recall of 99.76%, with a reduction to 37 components, of which an average of 13.3 were selected). Additionally, the combination of PCA–Fisher and PCA–RFE algorithms was unable to converge on all algorithms as well as the selected algorithms i.e., SVM–0FE and Bagging–Elastic Net.

The results of the JM1 data set, found in Figure 24, consist of 21 features, a sample size of 7782 instances, of which 1672 were true, containing defects, and 6110 false, without defect, and the true percentage accounting for 21.48%. The top three algorithms were Stacking–PCA–Fisher (average accuracy of 79.57%, average precision of 61.76%, average F-measure of 23.52%, and an average recall of 14.9%, with a reduction to 15 components of which 12 were selected). The remaining two were SVM–Base (average accuracy of 79.36%, average precision of 62.61%, average F-measure of 18.21%, and an average recall of 10.82%, using the total number 80 features), and LR–PCA–RFE (average accuracy of 79.26%, average precision of 59.1%, average F-measure of 20.6%, and an average recall of 12.74%, with a reduction to 17 components, of which 16 were selected).

The results for the JM1 data set that utilized SMOTE, found in Figure 25, showed the top three algorithms were RF–PLS (average accuracy of 82%, an average precision of 80.43%, an average F-measure of 82.45%, and a recall of 84.6%, with a reduction to 17 components), followed by RF–PLS–Fisher (average accuracy of 81.75%, an average precision of 80.39%, an average F-measure of 82.14%, and a recall of 83.99%) and RF–CA–Elastic Net (average accuracy of 81.64%, an average precision of 80.17%, an average F-measure of 82.06%, and a recall of 84.07%) both of which showed a reduction to 17 components, of which 16 were selected.

The results for KC1 data set, shown in Figure 26, consist of 21 features, a sample size of 1183 instances, of which 314 were true, containing defects, and 869 false without defect, and the true percentage accounting for 26.54%. The top three performing algorithms were Stacking–PLS (average accuracy of 77.01%, average precision of 72.98%, average F-measure of 35.38%, and average recall of 24.13%, with a reduction to 18 components). The remaining two were MLP–PCA–Fisher (average accuracy of 76.84%, average precision of 64.95%, average F-measure of 30.14%, and average recall of 40.37%, with a reduction to 11 components, of which 6 were selected), and Stacking–PCA, (average accuracy of 76.67%, average precision of 65.03%, average F-measure of 36.12%, and average recall of 25.58%, with a reduction to 13 components).

The results for the KC1 data set that utilized SMOTE, found in Figure 27, reveal the top three algorithms were RF-PLS-RFE, (average accuracy of 81.07%, an average precision of 80.61%, an average F-measure of 81.06%, and a recall of 81.68%, with a reduction to 15 components, 13 of which were selected). This was followed by RF–PLS–Fisher (average accuracy of 80.21%, an average precision of 79.7%, an average F-measure of 80.21%, and a recall of 81.68%, with a reduction to 14 components, 13 being selected). RF–PLS (average accuracy of 80.1%, an average precision of 79.82%, an average F-measure of 80.15%, and a recall of 80.91%, with a reduction to 17 components).

The results for the KC3 data set, shown in Figure 28, comprise 39 features, a sample size of 194 instances, of which 36 were true, containing defects, and 158 false without defect, and the true percentage accounting for 18.55%. The top four results were KNN–RFE (average accuracy of 84.53%, average precision of 70%, average F-measure of 44.5%, and an average recall of 39.26%, with a selection of 8 features), followed by KNN–RFE (average accuracy of 84.53%, average precision of 70%, average F-measure of 44.5%, and an average recall of 39.26%, with a selection of 8 features), and KNN–ELastic Net (average accuracy of 84.53%, average precision of 75%, average F-measure of 40.05%, and an average recall of 29.83%, with an average feature selection of 6.2). The fourth was LR–PLS–Fisher (average accuracy of 84.45%, average precision of 67.67%, average F-measure of 50.38%, an average recall of 45.19%, with a reduction to 4 components, of which 3 were selected).

The results for the KC3 data set that utilized SMOTE are found in Figure 29. The top three algorithms were XGBoost-PLS (average accuracy of 93.69%, an average precision of 90.45%, an average F-measure of 93.8%, and a recall of 97.8%, with a reduction to 34 components), followed by RF–PLS–RFE (average accuracy of 93.33%, an average precision of 91.38%, an average F-measure of 92.91%, and a recall of 94.78%, with a reduction to 27 components, of which 24 were selected), and RF–PLS–Fisher (average accuracy of 92.74%, an average precision of 91.65%, an average F-measure of 90.06%, and a recall of 89.56%, with a reduction to 24 components, from which 23 were selected).

The results of Log4j data set are presented in Figure 30 and consist of 80 features, a sample size of 205 instances, of which 189 were true, containing defects, and 16 false without defect, and the true percentage accounting for 92.19%. The three top performing models were KNN–PCA–Fisher (average accuracy of 93.21%, average precision of 93.07%, average F-measure of 96.17% and an average recall of 100%, with a reduction to 73 components, of which 52 were selected), followed by RF–Elastic Net (average accuracy of 93.12%, average precision of 93.41%, average F-measure of 96.26% and an average recall of 99.47%, with an average selection of 7.3 features), and NB–PCA (average accuracy of 92.74%, average precision of 92.71%, average F-measure of 96.15% and an average recall of 100%, with a reduction to 70 components).

The results for the Log4j 1.2 data set that utilized SMOTE are found in Figure 31, showing the top three algorithms were Stacking-PLS-Fisher (average accuracy of 99.2%, an average precision of 100%, an average F-measure of 99.21%, and a recall of 98.46%, with a reduction to 49 components, of which 43 were ultimately selected), followed by XGBoost-PLS (average accuracy of 98.41%, an average precision of 100%, an average F-measure of 98.64%, and a recall of 97.38%, with a reduction to 41 components), and RF–PLS (average accuracy of 98.15%, an average precision of 99.38%, an average F-measure of 98.12%, and a recall of 96.94%, with a reduction of 24 components). In addition, with the exception of RF–PCA–Fisher, the other PCA–Fisher and PCA–RFE algorithms were unable to converge.

The results of the Lucene 2.4 data set are shown in Figure 32. This data set consisted of 80 features, a sample size of 339 instances, of which 203 were true, containing defects, and 136 false without defect, and the true percentage accounting for 59.88%. The three top performing models were SVM–RFE *average accuracy of 76.39%, average precision of 80.33%, average F-measure of 80.49% and an average recall of 81.77%, with a selection of 41 features). SVM had the best initial Base results, and, additionally, revealed the best results as compared to Fisher, PCA, and RFE. Among the other algorithms were Stacking–Base (average accuracy of 74.33%, average precision of 76.02%, average F-measure of 79.42% and an average recall of 84.01%, using all 80 features), and KNN–Fisher (average accuracy of 73.47%, average precision of 76.57%, average F-measure of 78.26% and an average recall of 81.57%, with a selection of 50 features).

The results for the Lucene 2.4 data set that utilized SMOTE are found in Figure 33 and present the top three algorithms as RF–PLS–Fisher (average accuracy of 81.27%, an average precision of 82.39%, an average F-measure of 80.07%, and a recall of 78.54%, with a reduction to 28 components, of which 27 were ultimately selected), followed by RF–PLS–RFE (average accuracy of 79.33%, an average precision of 80.62%, an average F-measure of 78.6%, and a recall of 77.85%, with a reduction to 40 components, of which 38 were selected), and XGBoost–Base (average accuracy of 79.08%, an average precision of 82.71%, an average F-measure of 82.13%, and a recall of 82.4%).

The results of the MC1 data set are presented in Figure 34, and consist of 38 features, a sample size of 1988 instances, of which 46 were true, containing defects, and 1942 false without defect, and the true percentage accounting for 2.31%. The three top performing models were Stacking–PLS–RFE (average accuracy of 99.34%, average precision of 96.67%, average F-measure of 16.5% and an average recall of 11.01%, with a reduction to 35 features, of which 32 were selected). Other than the other stacking models PCA–Fisher, PCA–Elastic Net, PCA–RFE, and PLS–Fisher, XGBoost–Base showed average accuracy of 97.79%, average precision of 85%, average F-measure of 17.52% and an average recall of 12.5%, using all 38 features. The other was MLP–PLS–Fisher (average accuracy of 97.74%, average precision of 100%, average F-measure of 12.86% and an average recall of 11.67%, with a reduction to 4 components, of which 2 were selected).

The results for the MC1 data set that utilized SMOTE are found in Figure 35 and reveal the top three algorithms as RF-PLS-Fisher (average accuracy of 99.46%, an average precision of 98.98%, an average F-measure of 99.46%, and a recall of 99.95%, with a reduction to 33 components, of which 31 were selected). This was followed by RF-PLS-RFE (average accuracy of 99.46%, an average precision of 99.01%, an average F-measure of 99.45%, and a recall of 99.89%, with a reduction to 33 components of which 31 were selected) and RF-PLS (average accuracy of 99.43%, an average precision of 98.98%, an average F-measure of 99.44%, and a recall of 99.78%. Additionally, Stacking, MLP and XGBoost show the comparable performance).

The results of the MC2 data set are presented in Figure 36 with 39 features, a sample size of 125 instances, of which 44 were true, containing defects and 81 false without defect, and the true percentage accounting for 35.2%. The three top performing models were LR–PCA–RFE (average accuracy of 75.26%, average precision of 70.67%, average F-measure of 61.49% and an average recall of 62.08%, with a reduction to 35 components, of which 20 were selected), followed by MLP–Fisher (average accuracy of 75.13%, average precision of 68.55%, average F-measure of 64.43% and an average recall of 67.83%, with 35 features selected), and NB–PCA–RFE (average accuracy of 74.94%, average precision of 71.83%, average F-measure of 58.04% and an average recall of 53.17%, with a reduction to 10 components, of which 9 were selected).

The results for the MC2 data set that utilized SMOTE are found in Figure 37 and present the top three algorithms as MLP–PLS–Fisher (average accuracy of 86.4%, an average precision of 84.07%, an average F-measure of 85.22%, and a recall of 88.79%, with a reduction to 31 components, of which 24 were selected), followed by RF–PCA–Elastic Net (average accuracy of 84.49%, an average precision of 83.51%, an average F-measure of 83.79%, and a recall of 84.89%, with a reduction to 20 components, of which an average of 10.6 were selected). MLP–PLS had average accuracy of 83.31%, an average precision of 82.32%, an average F-measure of 83.44%, and a recall of 87.24%, with a reduction to 8 components.

The results of the MW1 data set are presented in Figure 38 with 39 features, a sample size of 253 instances, of which 27 were true, containing defects, and 226 false without defect, and the true percentage accounting for 10.67%. The three top performing models were MLP–Elastic Net (average accuracy of 91.31%, average precision of 75%, average F-measure of 45% and an average recall of 40.83%, with an average selection of 5.4 features), followed by SVM–Elastic Net (average accuracy of 90.94%, average precision of 85%, average F-measure of 33% and an average recall of 26.17%, with an average selection of 5 features), and LR–PCA–Elastic Net (average accuracy of 90.88%, average precision of 85%, average F-measure of 26.67% and an average recall of 27.83%, with a reduction to 9 components, of which an average of 4.4 were selected).

The results for the MW1 data set that utilized SMOTE are found in Figure 39 and reveal the top four algorithms as RF–PLS (average accuracy of 97.35%, an average precision of 96.28%, an average F-measure of 97.29%, and a recall of 98.47%, with a reduction to 30 components), followed by Stacking–PLS–RFE (average accuracy of 96.68%, an average precision of 95.15%, an average F-measure of 96.64%, and a recall of 98.28%, with a reduction to 32 components, of which 31 were selected), and GBoost–PLS–Fisher (average accuracy of 96.46%, an average precision of 94.1%, an average F-measure of 96.54%, and a recall of 99.18%, with a reduction to 31 components of those 27 were selected). RF–PLS–Fisher had average accuracy of 96.46%, an average precision of 95.13%, an average F-measure of 96.52%, and a recall of 98.07%, with a reduction to 30 components of which 28 were selected.

The results of the PC1 data set are presented in Figure 40 with 37 features, a sample size of 705 instances, of which 61 were true, containing defects, and 644 false without defect, and the true percentage accounting for 8.65%. The three top performing models were Bagging–RFE (average accuracy of 92.5%, average precision of 70%, average F-measure of 43.22% and an average recall of 35.37%, with 18 features selected), followed by LR–PCA (average accuracy of 92.49%, average precision of 85%, average F-measure of 28.44% and an average recall of 18.85%, with a reduction to 2 components), and LR–PLS–Fisher (average accuracy of 92.2%, average precision of 65%, average F-measure of 31.52% and an average recall of 28.48%, with a reduction to 4 components, of which 2 were selected).

The results for the PC1 data set that utilized SMOTE are found in Figure 41 and present the top three algorithms as RF–PLS–Fisher (average accuracy of 97.98%, an average precision of 96.72%, an average F-measure of 98.02%, and a recall of 99.38%, with a reduction to 32 components, of which 31 were selected), followed by MLP–PLS (average accuracy of 97.21%, an average precision of 94.78%, an average F-measure of 97.31%, and a recall of 100%, with a reduction to 15 components), and Stacking–PLS (average accuracy of 97.12%, an average precision of 95.4%, an average F-measure of 97.1%, and a recall of 98.89%, with a reduction to 25 components).

The results of the PC2 data set are presented in Figure 42, with 36 features, a sample size of 745 instances, of which 16 were true, containing defects, and 729 false without defect, and the true percentage accounting for 2.31%. The three top performing models were KNN–PLS–Fisher (average accuracy of 97.86%, average precision of 100%, average F-measure of 40% and an average recall of 40%, with a reduction to 7 components, of which 6 were selected), followed by Stacking–PCA (average accuracy of 97.85%, average precision of 100%, average F-measure of 40% and an average recall of 40%, with a reduction to 25 components), and SVM–PLS–RFE (average accuracy of 97.85%, average precision of 100%, average F-measure of 30% and an average recall of 30%, with a reduction to 4 components, of which 2 were selected). Although other models performed better in terms of accuracy, due to class imbalance, both the F-measure and Recall were less accurate, and, thus, both were less desirable.

The results for the PC2 data set that utilized SMOTE are ound in Figure 43 and reveal the top three algorithms as Stacking–PLS–Fisher *average accuracy of 99.52%, an average precision of 99.09%, an average F-measure of 99.54%, and a recall of 100%, with a reduction to 30 components, of which 23 were selected), followed by Stacking–PLS *average accuracy of 99.45%, an average precision of 99.08%, an average F-measure of 99.47%, and a recall of 99.87%, with a reduction to 30 components), and RF–PLS (average accuracy of 99.45%, an average precision of 99.08%, an average F-measure of 99.47%, and a recall of 99.88%, with a reduction to 20 components). Additionally, the PCA–Fisher and PCA–RFE algorithms were difficult converge.

The results of the PC3 data set are presented in Figure 44 with 37 features, a sample size of 1077 instances, of which 134 were true, containing defects, and 943 false without defect, and the true percentage accounting for 12.44%. The three top performing models were LR–PCA–Elastic Net (average accuracy of 87.93%, average precision of 59.33%, average F-measure of 20.62% and an average recall of 12.99%, with a reduction to 12 components, of with an average of 5.1 features were selected), followed by NB–PCA (average accuracy of 87.93%, average precision of 59.33%, average F-measure of 20.62% and an average recall of 12.99%, with a reduction to 2 components). It was observed that NB–PLS–Fisher, and NB–PLS performed better than other models. The class imbalance affected the results of many models with low F-Measure and Recall. Another suitable model would be XGBoost–Elastic Net (average accuracy of 86.82%, average precision of 49.46%, average F-measure of 31.17% and an average recall of 23.67%, with an average selection of 9.4 features).

The results for the PC3 data set that utilized SMOTE are found in Figure 45 and present the top three algorithms as MLP–PLS–Fisher (average accuracy of 93.85%, an average precision of 90.74%, an average F-measure of 94.1%, and a recall of 97.8%, with a reduction to 27 components, of which 24 were selected), followed by MLP–PLS (average accuracy of 93.53%, an average precision of 89.95%, an average F-measure of 93.78%, and a recall of 97.97%, with a reduction to 27 components), and XGBoost–PLS–Fisher (average accuracy of 93.37%, an average precision of 90.05%, an average F-measure of 93.53%, and a recall of 97.4%, with a reduction to 31 components, of which 29 were selected).

The results of the PC4 data set are presented in Figure 46 with 37 features, a sample size of 1287 instances, of which 177 were true, containing defects, and 1110 false without defect, and the true percentage accounting for 13.75%. The three top performing models were Stacking–PCA–RFE (average accuracy of 90.37%, average precision of 74.21%, average F-measure of 57.27% and an average recall of 48.58%, with a reduction to 34 components, of which 32 were selected), XGBoost–Elastic Net (average accuracy of 89.9%, average precision of 62.41%, average F-measure of 63.93% and an average recall of 66.67%, with an average selection of 10.8 features), and SVM–PLS (average accuracy of 89.82%, average precision of 84.58%, average F-measure of 44.53% and an average recall of 31.73%, with a reduction to 10 components).

The results for the PC4 data set that utilized SMOTE are found in Figure 47 and present the top three algorithms as MLP–PLS (average accuracy of 96.58%, an average precision of 94.55%, an average F-measure of 96.62%, and a recall of 98.81%, with a reduction to 28 components), followed by MLP–PLS–Fisher (average accuracy of 96.22%, an average precision of 94.13%, an average F-measure of 96.24%, and a recall of 98.53%, with a reduction to 26 components, of which 25 were selected), and MLP–PLS–RFE (average accuracy of 95.95%, an average precision of 93.37%, an average F-measure of 96.03%, and a recall of 98.87%, with a reduction to 22 components, of which 21 were selected). Additionally, XGBoost and RF showed comparable performance among the other algorithms.

The results of the PC5 data set are presented in Figure 48 with 38 features, a sample size of 1711 instances, of which 471 were true, containing defects, and 1240 false without defect, and the true percentage accounting for 27.52%. The three top performing models were Stacking–PCA–Fisher (average accuracy of 77.09%, average precision of 66.73%, average F-measure of 45.3% and an average recall of 34.96%, with a reduction to 13 components, of which 9 were selected), followed by Stacking–Base (average accuracy of 77.09%, average precision of 68.39%, average F-measure of 43.97% and an average recall of 33.13%, with all 38 features), and Stacking–Elastic Net (average accuracy of 76.91%, average precision of 66.46%, average F-measure of 43.49% and an average recall of 32.88%, with an average of 11 features selected).

The results for the PC5 data set that utilized SMOTE are found in Figure 49, and consist of the top three algorithms as XGBoost–PLS (average accuracy of 84.23%, an average precision of 82.39%, an average F-measure of 84.61%, and a recall of 87.14%, with a reduction to 33 components), followed by RF–PLS–Fisher (average accuracy of 83.99%, an average precision of 81.66%, an average F-measure of 84.55%, and a recall of 87.73%, with a reduction to 33 components, of which 29 were selected), and MLP–PLS (average accuracy of 83.55%, an average precision of 82.28%, an average F-measure of 83.83%, and a recall of 85.55%, with a reduction to 27 components).

The results of the Poi 3.0 data set are shown in Figure 50 with 80 features, a sample size of 442 instances, of which 281 were true, containing defects, and 161 false without defect, and the true percentage accounting for 63.57%. The three top performing models were KNN–PCA–Elastic Net (average accuracy of 80.97%, average precision of 84.2%, average F-measure of 85.28% and an average recall of 86.9%, with a reduction to 50 components, of which an average of 16.7 features were selected). Although the KNN–PLS, KNN–PCA, and KNN–Fisher also performed very well, compared to other learning algorithms, the remaining two were SVM–PLS (average accuracy of 79.42%, average precision of 84.34%, average F-measure of 83.66% and an average recall of 83.46%, with a reduction to 6 components), and RF–PCA–Elastic Net (average accuracy of 78.94%, average precision of 82.13%, average F-measure of 83.6% and an average recall of 85.69%, with a reduction to 69 components, of which an average of 10.8 features were selected).

The results for the Poi 3.0 data set that utilized SMOTE are found in Figure 51 and show the top three algorithms were RF-PLS-RFE (average accuracy of 84.34%, an average precision of 86.29%, an average F-measure of 84.62%, and a recall of 81.76%, with a reduction to 66 components, of which 30 were selected), followed by RF–PLS–Fisher (average accuracy of 84.33%, an average precision of 86.54%, an average F-measure of 83.71%, and a recall of 81.9%, with a reduction to 69 components, of which 66 were selected), and Stacking–PLS–RFE (average accuracy of 83.98%, an average precision of 86.07%, an average F-measure of 83.42%, and a recall of 81.3%, with a reduction to 63 components, of which 57 were selected).

The results of the Synapse 3.0 data set are depicted in Figure 52 with 80 features, a sample size of 256 instances, of which 86 were true, containing defects, and 170 false without defect, and the true percentage accounting for 32.59%. The three top performing models were RF–RFE (average accuracy of 78.86%, average precision of 77.57%, average F-measure of 62.18% and an average recall of 53.23%, with 45 features selected), followed by Stacking–PCA (average accuracy of 78.83%, average precision of 73.58%, average F-measure of 64.86% and an average recall of 59.12%, with a reduction to 73 components), and LR–RFE (average accuracy of 78.52%, average precision of 72.88%, average F-measure of 61.82% and an average recall of 55.85%, with 7 features selected).

The results for the Synapse 3.0 data set that utilized SMOTE are found in Figure 53, showing the top three algorithms were RF–PLS–RFE (average accuracy of 87.06%, an average precision of 87.44%, an average F-measure of 86.99%, and a recall of 87.39%, with a reduction to 71 components, of which 47 were selected), followed by MLP–PLS–Fisher (average accuracy of 85.29%, an average precision of 83.11%, an average F-measure of 85.84%, and a recall of 90.33%, with a reduction to 25 components, of which 23 were selected) and MLP–PLS (average accuracy of 84.71%, an average precision of 82.92%, an average F-measure of 84.88%, and a recall of 87.66%, with a reduction to 31 components).

The results of the Velocity 1.6 data set are shown in Figure 54 with 80 features, a sample size of 228 instances, of which 78 were true, containing defects, and 150 false without defect, and the true percentage accounting for 34.21%. The three top performers were MLP–PCA–RFE (average accuracy of 77.65%, average precision of 67.77%, average F-measure of 67.09% and an average recall of 69.04%, with a reduction to 39 components, of which 34 were selected), SVM–PCA (average accuracy of 77.57%, average precision of 72.79%, average F-measure of 64.15% and an average recall of 59.8%, with a reduction to 54 features), and Stacking–Base (average accuracy of 77.55%, average precision of 74.29%, average F-measure of 59.68% and an average recall of 51.43%, with all 80 features).

The results for the Velocity 1.6 data set that utilized SMOTE are found in Figure 55, presenting the top three algorithms as MLP–PLS–RFE (average accuracy of 88.33%, an average precision of 87.16%, an average F-measure of 88.57%, and a recall of 90.81%, with a reduction to 29 components of which 28 being selected), followed by RF–PLS–RFE (average accuracy of 88.33%, an average precision of 87.99%, an average F-measure of 87.24%, and a recall of 87.26%, with a reduction to 71 components of which 37 were selected) and MLP–PLS (average accuracy of 87.67%, an average precision of 84.75%, an average F-measure of 87.83%, and a recall of 91.85%, with a reduction to 69 components).

The results of the Xalan 2.7 data set, presented in Figure 56, had 80 features, a sample size of 909 instances, of which 898 were true, containing defects, and 11 false without defect, and the true percentage accounting for 98.78%. The top four performing models were KNN–Elastic Net, where, on average, there was a selection of 49.3 features, MLP–Base, using all the 80 features,, MLP–PLS seeing a reduction to 13 components, and MLP–Fisher where 39 features were selected each having (average accuracy of 99.45%, average precision of 99.45%, average F-measure of 99.72% and an average recall of 100%). In addition, other models that performed well included NB, RF, KNN, LR, Bagging, stacking and XGBoost. The LR, Stacking, and SVM learning algorithms could not converge on the data set, due to class imbalance in the PCA–RFE and PCA–Elastic Net combined algorithm models.

The results for the Xalan 2.7 data set that utilized SMOTE are found in Figure 57 and show the top three algorithms were RF–PLS–Fisher (average accuracy of 99.83%, an average precision of 100%, an average F-measure of 99.84%, and a recall of 99.68%, with a reduction to 10 components, of which 8 were selected), followed by XGBoost–PLS–Fisher (average accuracy of 99.67%, an average precision of 99.89%, an average F-measure of 99.68%, and a recall of 99.46%, with a reduction to 19 components, of which 16 were selected) and RF–PLS (average accuracy of 99.61%, an average precision of 100%, an average F-measure of 99.61%, and a recall of 99.23%, with a reduction to 10 components). Additionally, the combination algorithms within PCA–Fisher and PCA–RFE, excluding the RF learning algorithm, had difficulty in proper convergence, resulting in errors, and other instances, outside of these, were Stacking–PLS, SVM–RFE, RF–Fisher, and Adaboost for both Fisher and RFE.

The results of the Xerces 2.0 data set are shown in Figure 58, with 80 features, a sample size of 546 instances, of which 396 were true, containing defects, and 150 false without defect, and the true percentage accounting for 72.52%. The top three performing models were MLP–Elastic Net (average accuracy of 91.94%, average precision of 93.73%, average F-measure of 94.34% and an average recall of 95.11%, with the average selection of 9.5 features). The remaining two were RF–Elastic Net (average accuracy of 91.23%, average precision of 92.68%, average F-measure of 94.01% and an average recall of 95.68%, the average selection of features being 12.4). and then the Stacking–Elastic Net (average accuracy of 91.02%, average precision of 91.81%, average F-measure of 94.07% and an average recall of 96.83%, where the average number of features selected was 13.8). Additionally, it was observed that the Elastic Net–FS technique outperformed most of the models.

The results for the Xerces 2.0 data set that utilized SMOTE are found in Figure 59 and show the top three algorithms were RF–PLS (average accuracy of 94.05%, an average precision of 93.9%, an average F-measure of 94%, and a recall of 94.19%, with a reduction to 10 components), followed by RF–Base (average accuracy of 93.95%, an average precision of 94.56%, an average F-measure of 95.81%, and a recall of 97.21%, with the use of all 80 features) and XGBoost–PLS (average accuracy of 93.94%, an average precision of 94.4%, an average F-measure of 93.94%, and a recall of 93.62%, with a reduction to 68 components). Additionally, Stacking, in relation to the combined algorithms PCA–RFE and PLS–RFE, converged with difficulty.

## 4. Discussion

It was observed through extensive experimental results, that both FE and FS techniques, separately and in combination with each other, are valuable in binary classification to enhance the performance of a model. It was observed and analyzed that results without the use of SMOTE seemed ambiguous, due to the class imbalances that existed to a great degree in some of the data sets, and also, in less improved areas (1–2%), more significant changes are needed to reduce these ambiguities. Moreover, without SMOTE there were also many instances where a FE, FS, or combined algorithm performed worse than the Base model, which gave no guarantee about performance of a model from one data set to another. It was also observed that, in extreme situations, class imbalance accuracy could not be counted as a possible metric for evaluation. With the addition of SMOTE, performance of all metrics improved over all data sets, although whether data sets with more than 30% minority class representation should make use of SMOTE should be considered. Furthermore, by finding the best reduction value for the technique over each data set, many instances were found where improvements may have been overlooked, due to specifying a specific reduction value for all algorithms.

FE without SMOTE in the PLS algorithms does not show fast and better performance, as compared to overwhelming improvements with SMOTE. Due to class imbalances, PCA and PLS performance can vary, and, thus, it is hard to find patterns to maximize the variants. PCA did not consider output and performed to the same extent as PLS with each algorithm over the PC1 data set, as shown in Figure 40, where performance improved from (1–2%), favoring both PLS and PCA. Once SMOTE was applied to the data set, the PLS algorithm identified patterns and showed better performance over the PCA algorithm, as can be seen in the PC1 data set that utilized SMOTE in Figure 41.

The PLS algorithm also contained a few instances where the observed performance could have a negative impact on the algorithm with PC3 SMOTE data set, as shown in Figure 45. The LR–PLS results showed reduced performance in precision, F-measure, and recall, Although the vast majority of the data sets and algorithms showed improvement over the Base and PCA, this did not mean that PCA performed worse and was a bad choice to be a reduction method. Instead, it cannot be said when SMOTE was applied the PLS outperformed. It was observed that most of the time PCA was dependent on the data set, while the performance of the algorithm could have a positive or negative impact on the results.

Our analysis showed that FS had better performance in the same as FE, by depending on the algorithm and the data set. Although an example of an FS algorithm that performed consistently better on most of the algorithms could be seen in the Xerces 2.0 data set without SMOTE in Figure 58, with the Elastic Net FS algorithm. FS methods can have both a positive and negative impact on the performance of a model; for example, the MC1 data set that utilized SMOTE, found in Figure 35. In the joint algorithms (PCA and PLS) without SMOTE, it was observed that both the PCA and PLS could have a positive or negative effect on performance. Surprisingly, once SMOTE was applied, the performances of both PCA–Fisher and PCA–RFE at Velocity 1.6 data set were almost similar to the performance of the Base, as shown in the Figure 55.

Additionally, it can be seen in a majority of the data sets with SMOTE applied that PCA–Elastic Net performed similarly to the combined algorithms (PLS–Fisher and PLS–RFE). As stated by Pandey et al. [22], PCA is a linear transformation, so, to make a better combination of Elastic Net–FS, using the regression functions in the algorithm is necessary. It was observed that PLS combined algorithms performed better in most cases. These results were contrary to the theory behind certain models performing better with certain FS techniques, or that one technique is superior to another, as suggested by the results in Mehta et al. [13] and Wang et al. [5].

The results of LASSO–SVM may have provided an improvement over the other tested models, but the concept of combined models has been overlooked in previous research. It was observed that all the techniques were appropriate for consideration and testing. It is not only the model, or the technique, but the data set, the model, and the technique, i.e., FE, FS, or a combination of all, that play the key role in the entire performance measurement.

## 5. Challenges, Limitations, and Recommendations for Future Work

To answer RQ 3 it is vital to highlight the challenges and limitations of ML techniques during implementation and analysis of the results. The first challenge was associated with FE techniques. PLS uses a Regression algorithm for predictions, so the transformations were extracted from the algorithm.

It would be good to combine the PLS technique with the FS technique i.e., Elastic Net, which does not accept values from the PLS algorithm. This is something of an anomaly because the values used in the PCA algorithm were suitable in the implementation of the Elastic Net algorithm. This was either an error in the implementation or an indication that not all the FE techniques are suitable for all the FS algorithms, and even ML algorithms may have issues with these transformed values. Thus, many challenges may occur when implementing these techniques in custom or Deep Learning algorithms.

The second, challenge related to techniques found in Figure 22 Jedit data set and the Xalan data set, found in Figure 56, could not converge on the LR–PCA–Elastic Net, on both the SVM and the Stacking PCA–Elastic Net and PCA–RFE. Since the experiment used 10-fold cross-validation, the amount of bug-classified instances was reduced and the models were unable to converge. The Stacking algorithm uses both SVM and LR in its implementation, and both of which could not converge. Thus, it can be said that the problem is related to SVM and LR algorithms, and also, although equally as likely, may lay in the combined models. An additional challenge was associated with these three ML algorithms, along with a few instances of RF, as in Figure 18, where they under-fit the model and made one sided predictions, due to insufficient features within the data set. These problems were only found within these models so can be considered a part of these ML algorithms.

The third challenge was associated with the number of iterations used in the MLP algorithm. By default, there were 100, which then increased to 10,000 iterations, and LR with the same default required 100,000 iterations. This would have undoubtedly had a negative impact on the time complexity of the algorithms. This may be something to consider when implementing these algorithms in certain environments where instances need to be calculated in a time sensitive manner. When an entire algorithm is prolonged then it would be difficult to add additional operations, such as FE and FS techniques.

One recommendation for future work would be to investigate the added time complexity of combined presented techniques with algorithms by comparing the training times and the prediction times. This would give insight into whether these methods could be used in the monitoring of input and output devices for defects that require some level of time sensitivity. As stated by Dhaya Battina [4], one of the issues regarding ML in Continuous Integration and Continuous Development is how often the model must be retrained due to decay. Thus, if a model decays faster than it can be trained properly it would not be able to integrate as an automated DevOps tool.

Another recommendation, due to the results of the Stacking and MLP models, is to investigate the introduction/creation of a Stacking ANN. This could provide more flexibility, among other data sets, while also providing accurate results from the ANN models. Although this could be a promising and intriguing experiment 9t could be quite costly, due to the length of time spent during training.

The recommendation for future work would be to look at the stacking of models with different Static metrics i.e., size, complexity, documentation, inheritance, cohesion, used in the PROMISEAnt to Xerces data sets. The division of algorithms should reduce noise and provide better indicators to find defects, thereby mitigating them in source code at faster speed.

The final recommendation for future work is to implement a project with the SDP data collection and eventual predictions integrated into the SDLC. This could provide an excellent use case for an organization’s implementation and/or data collection for future SDP deployment. This could also allow the development of new or improved metrics to increase the accuracy of the models.

## 6. The Healthcare Use Case

For the healthcare industry, quality assurance is a crucial aspect in the deployment of Internet of Things (IoT)-based wearable devices and applications. The governmental requirements associated with the development of these devices and applications need SDLC to ensure a degree of traceability without any specifications for requirements and methodological type. Healthcare industries and applications are evolving and shifting towards DevOps, due to its faster delivery, improved collaboration, scalability, high level of security, and reliability. Furthermore, integration of DevOps and SDLC shows better compatibility during SDP project implementation.

SDP has the potential to reduce the defects within a project’s life-cycle, by improving the reliability of the product being delivered. To maintain traceability among other regulations (not withstanding ML regulations) the best practice is to implement SDP in an MLOps environment. Industries use the SDLC/DevOps, based on operational requirements, to collect required static features of SDP models for easy implementation into the SDLC process during data collection. In addition, due to governmental regulations, the classification of known defects can easily be conducted when product reviews are conducted.

**Figure 60:**
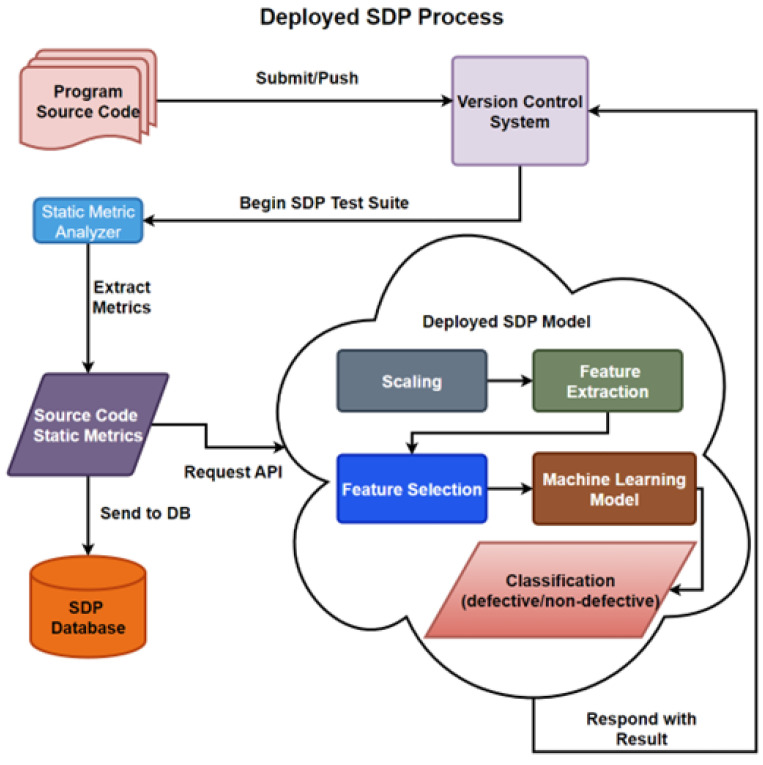
The Deployed SDP Process.

Figure 60 is a simple example of how a program could be implemented when new changes are made into a source code to send to the control system. Then, a program is executed in which the static metric analyzer extracts the static metrics to send an instance to the SDP database. Then, an API request is sent to the deployed model for prediction. Industries generate and handle large amounts of data, and usage of SDLC/DevOps methodologies provides level 2 MLOps maturity when following the practices for incorporating traceability.

The first step in developing MLOps pipelines is to understand the data pipeline. SDP is an aspect of continuous testing in DevOps to acquire data within the project pipeline. This data can include the product metrics (found in the tested data sets extracted from the source code) and other metrics (determined by the data scientist, clients, and data stewards), as discussed by Ruf et al. [11].

The organization could then manually train and validate the models until a threshold level is reached for the deployment model to allocate the required resources. Once this junction is reached, it is important that level 2 maturity has been archived for some tools within the SDLC to monitor and retrain the model with logs and other features during deployment. Then, data set can be revised to a DB to retrain the model to register, and create a pipeline for automated training during deployment to achieve level 3 maturity of MLOps.

Level 4 maturity, with automated model deployment and, eventually, a fully-automated MLOps process for an updated model with the latest data sets of IoT software would improve the reliability of released products.

## 7. Conclusions

SDP is an underdeveloped and underutilized testing tool that is potentially an integral aspect of SDLC. Through the expansion of concepts into real-world applications of DevOps, the continuous testing suite of processes and tools can provide additional traceability, and aid in defect reduction. One of the limiting factors in the research into SDP is the lack of contextual data from real-world applications. In a project with an existing data set in use, contextual metrics can be adapted, and applicable models can be further refined with the conducted tests.

The healthcare industry requires traceability of medical devices in the SDLC, particularly wearable devices, in addition to having other requirements based on the context of security. Additionally, the adoption of modern DevOps within the industry, as well as requirements for the adoption of SDP into development, could improve the performance of the models by increasing traceability.

This could be done through the use of metrics within the logs of the existing DevOps tools and process. The continuous development of the SDP tool in the MLOps would help in investigating new metrics in SDLC for traceability. This is not only a pre-requisite for the development of the healthcare industry, and medical wearable devices, but also for MLOps pipeline implementation.

Instead of having a limited sub-optimal sample size to aging data sets, their static metrics can be considered good starting points for advanced level expansion. The models still perform well, due to less disruptive traits of class imbalances. The modification of data metrics (i.e., to increase the sample size) improves the results significantly. ML algorithms in a DevOps environment (i.e., Agile) greatly impact model evolution because of rapid releases. Due to faster releases, more samples can be obtained, which leads to more insight into the patterns for the models. This could increase research into SDP, due to rapid changes and faster evaluation of the data sets, to explore more new metrics or theories.

This research focused on the high dimensionality problem containing noise within data that has a negative impact on a model. Reducing dimensions of the data noise can improve performance of a model. This article also focused on data reduction, transformation, and selection of methods, such as, PCA, and PLS for FE. In addition, Fisher Score, RFE, and Elastic Net methods were adopted. The proposed FE and FS methods were tested separately, and in combination with the Base models (i.e., AdaBoost, Bagging, DT, KNN, LR, MLP, NB, RF, Stacking, SVM, and XGBoost).

Conclusions were obtained for the binary classification of defects in the top performance models over the data sets, as well as the performance of other models. With regard to quantification metrics, when taking into account extreme minority classes, the accuracy metric is less suitable in evaluating results. To answer RQ2 FE techniques were examined, i.e., PCA, and most cases saw either negligible improvement over the Base model or negative impact on the performance of the model. PLS revealed similar performance to PCA without SMOTE, but showed consistent improvement with SMOTE. Both PCA and PLS are appropriate for reduction of high dimensional data sets, although performance cannot be guaranteed from one data set to the next. The performance of FS techniques, i.e., Fisher Score, RFE, and Elastic Net, were somewhat similar to PCA but varied for different algorithms with different data sets.

The results were somewhat contradictory as to when FE or FS techniques perform better with certain algorithms, and the combination of both can be considered during building a model to possibly produce better results. The addition of SMOTE meant most of the data sets presented consistent improvements in performance by combining with PLS algorithms. In addition, the PCA–Elastic Net model showed comparable improvements in consistency and performance over most of the data sets. The algorithms that outperformed in combination with a number of techniques and data sets were LR, KNN, MLP, RF, SVM, and Stacking.

## Figures and Tables

**Table 1:** Static Size Metrics that were extracted using the Open Static Analyzer.

	Size Metrics	
Lines of Code (LOC)	Logical Lines of Code (LLOC)	Number of Attributes (NA)
Number of Getters (NG)	Total Number of Local Public Methods (TNLPM)	Total Number of Local Setters (TNLS)
Number of Interfaces (NIN)	Total Number of Packages (TNPKG)	Total Number of Methods (TNM)
Number of Local Getters (NLG)	Total Number of Public Interfaces (TNPIN)	Number of Local Methods (NLM)
Number of Local Public Attributes (NLPA)	Total Number of Setters (TNS)	Total Number of Statements (TNOS)
Number of Packages (NPKG)	Number of Parameters (NUMPAR)	Number of Public Attributes (NPA)
Number of Statements (NOS)	Total Lines of Code (TLOC)	Total Number of Classes (TNCL)
Total Number of Enums (TNEN)	Total Number of Files (TNFI)	Total Number of Getters (TNG)
Number of Local Public Methods (NLPM)	Total Number of Directories (TNDI)	Number of Setters (NS)
Total Number of Public Enums (TNPEN)	Total Number of Public Attributes (TNPA)	Number of Enums (NEN)
Total Number of Public Classes (TNPCL)	Number of Classes (NCL)	Number of Local Attributes (NLA)
Total Number of Public Methods (TNPM)	Number of Local Setters (NLS)	Number of Public Methods (NPM)
Total Logical Lines of Code (TLLOC)	Total Number of Local Methods (TNLM)	Number of Methods (NM)

**Table 2:** Additional Static Metrics that were extracted using the Open Static Analyzer.

Complexity Metrics	Code Duplication Metrics	Coupling Metrics	Documentation Metrics
Halstead Calculated Program Length (HCPL)	Clone Age (CA)	Coupling Between Object Classes (CBO)	API Documentation (AD)
Halstead Difficulty (HDIF)	Clone Classes (CCL)	Coupling Between Object Classes Inverse (CBOI)	Comment Density(CD)
Halstead Effort (HEFF)	Clone Complexity (CCO)	Number of Incoming Invocations (NII)	Comment Lines of Code (CLOC)
Halstead Number of Delivered Bugs (HNDB)	Clone Coverage(CC)	Number of Outgoing Invocations (NOI)	Documentation Lines of Code (DLOC)
Halstead Program Length (HPL)	Clone Embeddedness (CE)	Response set For Class (RFC)	Public Documentation API (PDA)
Halstead Program Vocabulary (HPV)	Clone Instances (CI)	**Inheritance Metrics**	Public Undocumented API (PUA)
Halstead Time Required to Program (HTRP)	Clone Line Coverage (CLC)	Depth of Inheritance Tree (DIT)	Total API Documentation (TAD)
Halstead Volume (HVOL)	Clone Lines of Code (CLLOC)	Number of Ancestors (NOA)	Total Comment Density (TCD)
Maintainability Index Microsoft Version (MMS)	Clone Logical Line Coverage (CLLC)	Number of Children (NOC)	Total Comment Lines of Code (TCLOC)
Maintainability Index SEI Version (MSEI)	Clone Variability (CV)	Number of Descendants (NOD)	Total Public Documented API (TPDA)
Maintainability Index Orginal Version (MI)	Lines of Duplicated Code (LDC)	Number of Parents (NOP)	Total Public Undocumented API (TPUA)
Maintainability Index Open Static Analyzer Version (MIOS)	Logical Lines of Duplicate Code (LLDC)	**Cohesion Metrics**	
McCabe’s Cyclomatic Complexity (MCC)	Normalized Clone Radius (NCR)	Lack of Cohesion in Methods 5 (LCOM5)	
Nesting Level (NL)			
Nesting Level Else-If (NLE)			
Weighted Methods per Class (WMC)			

**Table 3:** Traditional Product Metrics found in the NASA Metrics Data Program repository.

Metric	Type	Definition
loc	Numeric	McCabe’s line count of code
v(g)	Numeric	McCabe “cyclomatic complexity”
ev(g)	Numeric	McCabe “essential complexity”
iv(g)	Numeric	McCabe “design complexity”
n	Numeric	Halstead total operators + operands
v	Numeric	Halstead “volume”
l	Numeric	Halstead “program length”
d	Numeric	Halstead “difficulty”
i	Numeric	Halstead “intelligence”
e	Numeric	Halstead “effort”
b	Numeric	Halstead
t	Numeric	Halstead’s time estimator
lOCode	Numeric	Halstead’s line count
lOComment	Numeric	Halstead’s count of lines of comments
lOBlank	Numeric	Halstead’s count of blank lines
lOCodeAndComment	Numeric	count of lines + comments
uniqOp	Numeric	unique operators
uniqOpnd	Numeric	unique operands
totalOp	Numeric	total operators
totalOpnd	Numeric	total operands
branchCount	Numeric	branch count of flow graph
defects	Boolean	Module has or has not a reported defect

**Table 4:** Data Sets.

Data Set	True	False	True(%)	Total	Features
Ant	166	579	22.28	745	80
Camel	188	739	20.28	927	80
CM1	42	285	12.84	327	37
Ivy	40	312	11.36	352	80
Jedit	11	481	2.23	492	80
JM1	1672	6110	21.48	7782	21
KC1	314	869	26.54	1183	21
KC3	36	158	18.55	194	39
Log4j	189	16	92.19	205	80
Lucene	203	136	59.88	339	80
MC1	46	1942	2.31	1988	38
MC2	44	81	35.2	125	39
MW1	27	226	10.67	253	37
PC1	61	644	8.65	705	37
PC2	16	729	2.14	745	36
PC3	134	943	12.44	1077	37
PC4	177	1110	13.75	1287	37
PC5	471	1240	27.52	1711	38
Poi	281	161	63.57	442	80
Synapse	86	170	33.59	256	80
Velocity	78	150	34.21	228	80
Xalan	898	11	98.78	909	80
Xerces	396	150	72.52	546	80

**Table 5:** Confusion Matrix.

Confusion Matrix		
	**Actual**	
**Reported**	**True**	**False**
True	True Positive	False Positive
False	False Negative	True Negative

## Data Availability

The cleaned version of NASA Metrics Data Program data used within the experiment can be found at https://figshare.com/collections/NASA_MDP_Software_Defects_Data_Sets/4054940 [NASA MDP]Shepperd, Martin; SOng, Qinbao; Sun, Zhongbin; Mair, Carolyn (2018): NASA MDP Software Defects Data Sets. figshare. Collection. https://doi.org/10.6084/m9.figshare.c.4054940.v1 and is the result of Shepperd et al. [23] research into quality of the NASA MDP data sets. The PROMISE data sets used within the experiment can be found at https://www.inf.u-szeged.hu/~ferenc/papers/UnifiedBugDataSet/ [PROMISE]Rudolf Ferenc; Zoltán Tóth; Gergely Ladányi; István Siket; and Tibor Gyimóthy; (2019)Unified Bug Dataset; University of Szeged Repository Version 1.2. The results and figures can be found at https://figshare.com/projects/A_Study_on_AI-based_Software_Defect_Detection_for_Security_Traceability_in_IoT_Applications/156449 in addition the code for the experiment is located in the public github project at https://github.com/sam-mcmurray/ML_SDP.

## Abbreviations

The following abbreviations are used in this manuscript:

SDP	Software Defect Prediction
SDLC	Software Development Life-Cycle
ML	Machine Learning
DevOps	Development Operations
MLOps	Machine Learning Operations
IOT	Internet of Things
FE	Feature Extraction
PCA	Principal Component Analysis
PLS	Partial Least Squares Regression
FS	Feature Selection
RFE	Recursive Feature Elimination
AdaBoost	Adaptive Boosting
SVM	Support Vector Machine
LR	Logistic Regression
NB	Naïve Bayes
KNN	K-Nearest Neighbor
MLP	Multilayer Perceptron
DT	Decision Tree
Bagging	Bootstrap Aggregation
XGBoost	Extreme Gradient Boosting
RF	Random Forest
Stacking	Generalized Stacking
MDP	Nasa Metrics Data Program
PROMISE	PredictOr Models In Software Engineering
UL	Unsupervised Learning
SL	Supervised Learning
AUC	Area Under Curve
ROC	Receiver Operating Characteristic
ANN	Artificial Neural Network
MFO	Moth Flame Optimization
IsBMFO	Island Binary Moth Flame Optimization
LDA	Fisher Linear Discriminant Analysis
CA	Cluster Analysis
BPNN	Back Propagation Neural Network
ENN	Elman Neural Network
LASSO	Least Absolute Shrinkage and Selection Operator
RBF	Radial Basis Function
SMOTE	Synthetic Minority Over-Sampling Technique
ELM	Extreme Learning Machine
KPCA	Kernel Principal Component Analysis
LSE	Least Squares Error
ET	Extra Trees
GB	Gradient Boosting
TP	True Positive
FP	False Positive
FN	False Negative
TN	True Negative
